# 2D Undulated Metal Hydrogen-Bonded Organic Frameworks with Self-Adaption Interlayered Sites for Highly Efficient C–C Coupling in the Electrocatalytic CO_2_ Reduction

**DOI:** 10.1007/s40820-025-01679-3

**Published:** 2025-02-24

**Authors:** Jianning Lv, Wenrui Li, Shuai Li, Shuo Xu, Zunhang Lv, Zhejiaji Zhu, Lu Dai, Bo Wang, Pengfei Li

**Affiliations:** 1 Key Laboratory of Cluster Science Ministry of Education, Beijing Key Laboratory of Photoelectronic/Electrophotonic, School of Chemistry and Chemical Engineering, Beijing Institute of Technology, Beijing, 100081 People’s Republic of China; 2 Advanced Technology Research Institute (Ji’nan), Beijing Institute of Technology, Ji’nan, 250300 People’s Republic of China; 3 Advanced Research Institute of Multidisciplinary Science, Beijing Institute of Technology (Zhuhai), Zhuhai, 519088 People’s Republic of China; 4 Petrochina Petrochemical Research Institute, Beijing, 102206 People’s Republic of China

**Keywords:** Hydrogen-bonded organic frameworks, Flexible structure, Self-adaption interlayered sites, Electrocatalytic CO_2_ reduction

## Abstract

The hydrogen-bonded organic frameworks (HOFs) as a new type of porous framework materials have been widely studied in various areas. However, the lack of appropriate active sites, low intrinsic conductivity, and poor stability limited their performance in the field of electrocatalysis. Herein, we designed two 2D metal hydrogen-bonded organic frameworks (2D–M–HOF, M = Cu^2+^ or Ni^2+^) with coordination compounds based on 2,3,6,7,14,15-hexahydroxyl cyclotricatechylene and transition metal ions (Cu^2+^ and Ni^2+^), respectively. The crystal structure of 2D–Cu–HOF is determined by continuous rotation electron diffraction, indicating an undulated 2D hydrogen-bond network with interlayered π-π stacking. The flexible structure of 2D–M–HOF leads to the formation of self-adaption interlayered sites, resulting in superior activity and selectivity in the electrocatalytic conversion of CO_2_ to C_2_ products, achieving a total Faradaic efficiency exceeding 80% due to the high-efficiency C–C coupling. The experimental results and density functional calculations verify that the undulated 2D–M–HOF enables the energetically favorable formation of *OCCHO intermediate. This work provides a promising strategy for designing HOF catalysts in electrocatalysis and related processes.

**Supplementary Information:**

The online version contains supplementary material available at 10.1007/s40820-025-01679-3.

## Highlights

- Highly crystalline 2D metal hydrogen-bonded organic frameworks (2D-M-HOFs) including 2D-Cu-HOF and 2D-Ni-HOF were designed and synthesized.
- The 2D-M-HOF with flexible ligands leads to the formation of the self-adaption interlayered sites, which facilitate the C–C couple and overcome the limitations of the coadsorption of multiple intermediates in the electrocatalytic CO_2_ reduction reaction.
- The undulated 2D-Cu-HOF exhibits outstanding activity and selectivity for electrocatalytic reduction of CO_2_ to C_2_ products with a total Faradaic efficiency of 82.1% (48.2% for C_2_H_5_OH and 33.9% for C_2_H_4_) at −1.2 V vs. RHE.

**Supplementary Information:**

The online version contains supplementary material available at 10.1007/s40820-025-01679-3.

## Graphical abstract

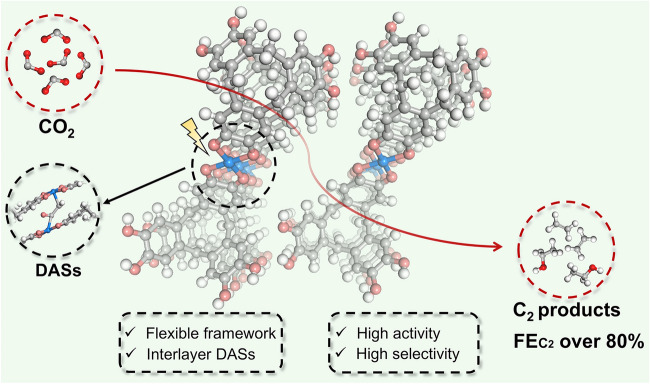

## Introduction

Hydrogen-bonded organic frameworks (HOFs) are a type of molecular-based porous crystalline materials that are self-assembled via intermolecular hydrogen-bonding and/or van der Waals interaction [1–7]. Unlike the well-established metal–organic framework and covalent-organic framework that are connected by strong coordinated or covalent bonds, HOFs constructed with hydrogen-bonding networks possess high flexibility, which endows them with a great potential to build unique and efficient active sites [8–12]. However, the number of reported HOFs with permanent porosity is limited and most of the structural units are rigid fused aromatic rings, representative examples are porphyrin [13, 14], pyrene [15, 16], and triptycene [2, 17]. Meanwhile, the low electrical conductivity and stability severely encumber their applications in electrochemistry [18–20].

The introduction of building blocks containing metal coordinated centers to form 2D metal HOFs not only provides monodisperse metal active sites but also promotes electrical conductivity within the framework via the d-π conjugation and π-π stacking [21–23]. Moreover, the geometric interlocking π–π stacking of organic ligands effectively strengthens the chemical and thermal stability of HOFs with permanent porosity [24, 25]. These unique properties make metal HOFs promising candidates for applications in electrocatalysis, such as oxygen evolution reaction [26], oxygen reduction reaction [22], and CO_2_ reduction reaction (CO_2_RR) [27]. During these electrocatalytic processes, the adsorption and activation of multiple intermediates are quite important [28–31]. Adsorption and activation of reactants or intermediates on the catalyst require dedicated designed active sites with suitable distance and favored low energy barrier pathways during the reaction [32–34]. Therefore, the flexibility of HOFs is essential for ultrahigh catalytic performance but has been scarcely explored, which mainly lacks suitable ligands.

Hexahydroxyl cyclotricatechylene (HHCC) has a unique flexible structure with a bowl-shaped shallow cavity, which has found important applications in host–guest chemistry, liquid crystal, and others [35–37]. HHCC units form a 2D plane with different linkages that exhibit an undulated flexible feature, which has not been observed in other rigid polyaromatic organic ligands [38, 39]. More importantly, the HHCC would be integrated into metal HOFs to construct the desired flexible frameworks, exposing the self-adaption interlayered active sites to facilitate C–C couple of electrocatalytic CO_2_RR. The flexible metal HOFs will overcome the limitations of the coadsorption of multiple intermediates and low stability. Even with these merits, the development of undulated metal HOFs for electrocatalysis is rare and remains a grand challenge.

Herein, we synthesized two HHCC-based 2D–M–HOF (2D–Cu–HOF and 2D–Ni–HOF) with open channels, undulated framework structures, and more importantly self-adaption interlayered sites. The crystal structure of 2D–Cu–HOF was thoroughly investigated with high-resolution transmission electron microscopy (HR-TEM) and continuous rotation electron diffraction (cRED). The coordination monomer consists of two HHCC units with ortho-hydroxyl groups and Cu^2+^, which interact to create an extended undulated 2D plane. The 2D–Cu–HOF reveals excellent electrocatalytic CO_2_RR activity and selectivity toward C_2_ product with Faradaic efficiency (FE) reaching 82.1% (48.2% for C_2_H_5_OH and 33.9% for C_2_H_4_) at − 1.2 V vs. reversible hydrogen electrode (RHE). Notably, the 2D–Ni–HOF also exhibits high selectivity (up to 35.6%) toward C_2_H_5_OH at − 1.3 V vs. RHE, in contrast to the classical Ni catalysts with a preference for CO. The excellent activity and selectivity of 2D–Cu–HOF and 2D–Ni–HOF toward electrocatalytic reduction of CO_2_ into C_2_ products can be attributed to the formation of self-adaption interlayer sites and the selection of the preferred reaction pathways enabled by the unique structural design of HOF.

## Experimental Section

### Materials

Copper acetate monohydrate (Cu(OAc)_2_·H_2_O), tetrabutylammonium hydroxide (TBAH), 37% HCl, 25% NH_3_·H_2_O, NaOH, KHCO_3_, triethylamine (TEA), ethylenediamine (EDA) were purchased from Energy Chemical Co., Ltd. Acetone, methanol (MeOH), *N*,*N*-dimethylformamide (DMF), *N*,*N*-dimethylacetamide (DMAc), dimethyl sulfoxide (DMSO), 1,4-dioxane, tetrahydrofuran (THF), dichloromethane (DCM), and isopropanol were purchased from Beijing Tongguang Fine Chemical Company. Veratrole, 38% aqueous formaldehyde, and 1 M BBr_3_ were purchased from J&K Scientific.

### Synthetical Procedure for 2D–M–HOF, Cu_3_(HHTP)_2_ and CuO_4_@PPy

#### Optimal Synthetical Procedure for 2D–Cu–HOF

Cu(OAc)_2_·H_2_O (0.0324 mmol, 6.488 mg) and HHCC (0.0217 mmol, 7.66 mg) were dissolved in 2 mL of H_2_O/MeOH (v/v = 9:1). The vial was sonicated for 30 min. Then 50 µL of 25% NH_3_·H_2_O was added to the above solution. The resulting solution was heated at 60 °C for 5 days in an isothermal oven. When the vial was cooled to room temperature, the mixture was centrifugated, and the deposit was washed with H_2_O (10 mL × 5) and acetone (10 mL × 3), successively. Finally, the obtained solid was dried at 60 °C under vacuum for 12 h, yielding a brown-black powder.

#### Optimal Synthetical Procedure for 2D–Ni–HOF

Ni(OAc)_2_·4H_2_O (0.0324 mmol, 8.08 mg) and HHCC (0.0217 mmol, 7.66 mg) were dissolved in 2.2 mL of H_2_O/DMF (v/v = 9:2). The vial was sonicated for 30 min. Then 100 µL of 25% NH_3_·H_2_O was added to the above solution. The resulting solution was heated at 80 °C for 3 days in an isothermal oven. When the vial was cooled to room temperature, the mixture was centrifugated, and the deposit was washed with H_2_O (10 mL × 5) and acetone (10 mL × 3), successively. Finally, the obtained solid was dried at 60 °C under vacuum for 12 h, yielding a brown powder.

#### Synthetical Procedure for Cu_3_(HHTP)_2_

Cu(OAc)_2_·H_2_O (17.58 mg) and 2,3,6,7,10,11-hexahydroxytriphenylene (13 mg) were dissolved in a mixed solution of 0.5 mL DMF and 1.5 mL water. The vial was sonicated for 5 min and heated at 80 °C for 24 h in an isothermal oven. After the vial was cooled to room temperature, the mixture was filtered and washed with H_2_O (10 mL × 5) and acetone (10 mL × 3), respectively. Finally, the resulting product was dried at 60 °C under vacuum for 12 h.

#### Synthetical and Loading Procedure for CuO_4_@PPy

Pyrocatechol (2.2 g, 20 mmol) in 50 mL of water was combined with Cu(OAc)_2_·H_2_O (16 g, 80 mmol) in 50 mL of water. After that, the mixture was added by a solution of potassium persulfate (5.4 g, 20 mmol) in 100 mL of water. After 20 min of stirring, the CuO_4_ sample was successfully synthesized. The mixture was filtered, and the black precipitate was washed with 200 mL water. The resulting product was dried at 60 °C for 12 h. Subsequently, 10 mg CuO_4_ and 10 mg polypyrrole were added to 4 mL ethanol. The mixture was stirred and heated for 24 h. Finally, CuO_4_@PPy was obtained by centrifuging and drying at 60 °C for 12 h.

## Results and Discussion

### Synthesis and Characterization of 2D–M–HOF

Firstly, HHCC was obtained by the cyclotrimerization of readily available veratrole and high-yield BBr_3_ demethylation (Scheme S1 and Figs. S1-S4). The synthesis condition of 2D–Cu–HOF was optimized by the one-pot solvothermal reaction of HHCC and Cu(OAc)_2_·H_2_O under different base catalysts, reaction temperature, time, and solvent combinations according to their powder X-ray diffraction patterns (PXRD, Figs. S5-S9). The optimized reaction condition is in a mixture of H_2_O and MeOH (v/v = 9:1) with 25% NH_3_·H_2_O as the base at 60 °C for 5 days. High crystallinity 2D–Cu–HOF was obtained as a brown–black solid in 78% yield. Simultaneously, the synthesis of 2D–Ni–HOF was carried out in a solution comprising a mixture of H_2_O and DMF in a volumetric ratio of 9:2 with 25% NH_3_·H_2_O at a temperature of 80 °C for 3 days.

**Fig. 1:**
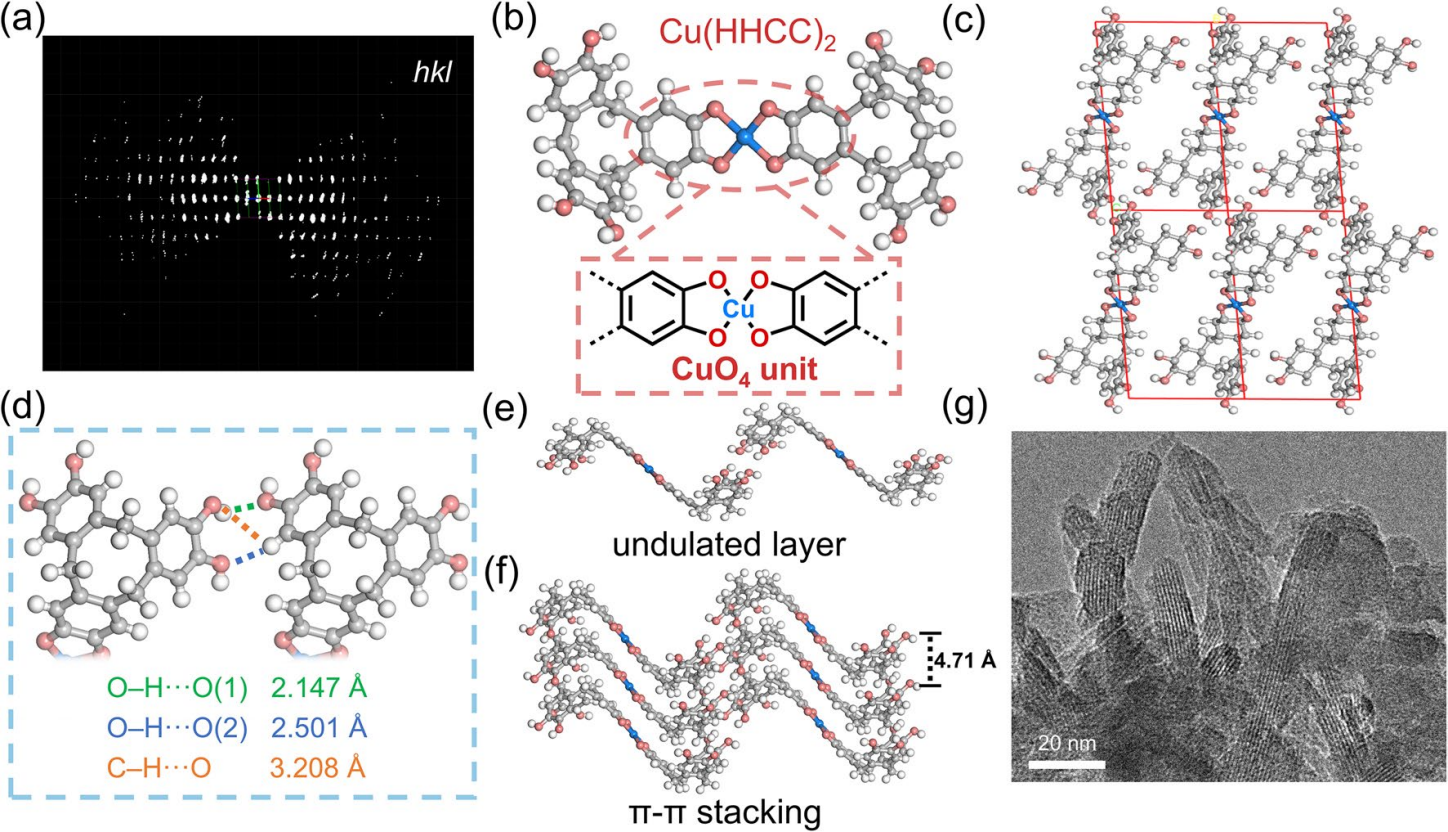
**a** Reconstructed 3D reciprocal lattice based on cRED of a 2D–Cu–HOF single crystal. **b** The structural model of Cu(HHCC)_2_. **c** The 2D–Cu–HOF crystal structural model along the *bc* plane. **d** The intermolecular hydrogen bond in the 2D–Cu–HOF. **e** The single layer structure and **f** the π-π stacking structure of 2D–Cu–HOF, white, gray, red, and blue spheres correspond to H, C, O, and Cu, respectively. **g** HR-TEM image of 2D–Cu–HOF

The Fourier-transform infrared (FTIR) spectra of 2D–Cu–HOF and 2D–Ni–HOF exhibit the significant disappearance of the O−H stretching band at 3334 cm^−1^, indicating the coordination between the metal ion (Cu^2+^ or Ni^2+^) and HHCC ligand (Fig. S10). Additionally, the C−O stretching vibration of 2D–Cu–HOF and 2D–Ni–HOF is shifted from 1337 cm^−1^ to a lower wavenumber of 1273 cm^−1^. 2D–Cu–HOF and 2D–Ni–HOF show a nano-rod morphology revealed by scanning electron microscopy (SEM) and TEM (Figs. S11 and S12). Furthermore, the crystal structure of 2D–Cu–HOF was investigated by cRED, HR-TEM, and PXRD (Fig. 1a). The cRED data provides unit cell parameters of *a* = 4.71 Å, *b* = 10.64 Å, *c* = 18.23 Å, *α* = 84.90°, *β* = 86.50°, and *γ* = 80.90°, corresponding to the ortho triclinic crystal system (Figs. S13, S14 and Table S1). Firstly, the coordination dimer Cu(HHCC)_2_ is formed by the coordination of Cu^2+^ with HHCC through the square-planar CuO_4_ unit (Fig. 1b and Table S2). Then, the Cu(HHCC)_2_ extends in the *bc* plane through hydrogen-bond interactions of the peripheral HO − of the HHCC ligand. The multiple hydrogen bonding is the dominant force between Cu(HHCC)_2_, which connects four adjacent Cu(HHCC)_2_ units and establishes a hydrogen bonding network in the 2D plane (Fig. 1c). Among these intermolecular interactions, there are two types of hydrogen bonds including O−H···O and C−H···O in the 2D–Cu–HOF with a distance of 2.147 ~ 2.501 and 3.208 Å, respectively (Fig. 1d). In the *a*-axis direction, the hydrogen-bonded 2D plane of 2D–Cu–HOF exhibits an undulated structural feature, which was further stacked with π–π stacking in an interlayer distance of 4.71 Å (Fig. 1e, f). The HR-TEM analysis of 2D–Cu–HOF reveals a distinct lattice fringe measuring 1.65 nm, aligning closely with the (001) crystallographic plane (Fig. 1g). For 2D–Cu–HOF, the (001), (011), and (012) diffractions can be observed at 5.6°, 11.1°, and 14.7°, respectively (Fig. S15). Moreover, the experimental PXRD of 2D–Ni–HOF is similar to that of 2D–Cu–HOF. The structural model of 2D–Ni–HOF was obtained by replacing the Cu atoms in the 2D–Cu–HOF with Ni atoms. The simulated PXRD of the optimized structure of 2D–Ni–HOF fitted well with the experimental PXRD with *R*_wp_ of 3.33% and *R*_p_ of 2.59% (Fig. S16). The HR-TEM image of 2D–Ni–HOF identifies a crystal plane spacing of 1.8 nm for the (001) plane (Fig. S17). Furthermore, the N_2_ sorption isotherm at 77 K was performed to evaluate the porosity of 2D–Cu–HOF and 2D–Ni–HOF (Figs. S18 and S19). The Brunauer–Emmett–Teller (BET) surface area of 2D–Cu–HOF is 265 m^2^ g^−1^ and the total pore volume is 0.254 cm^3^ g^−1^ at *P*/*P*_0_ = 0.99. The pore size distribution of 2D–Cu–HOF was analyzed by the non-local density functional theory (NL-DFT), which showcased consistent micropores with the theoretical model in a diameter of 7.6 Å. 2D–Ni–HOF shows a lower BET surface area of 65 m^2^ g^−1^, which may be attributed to its low crystallinity.

**Fig. 2:**
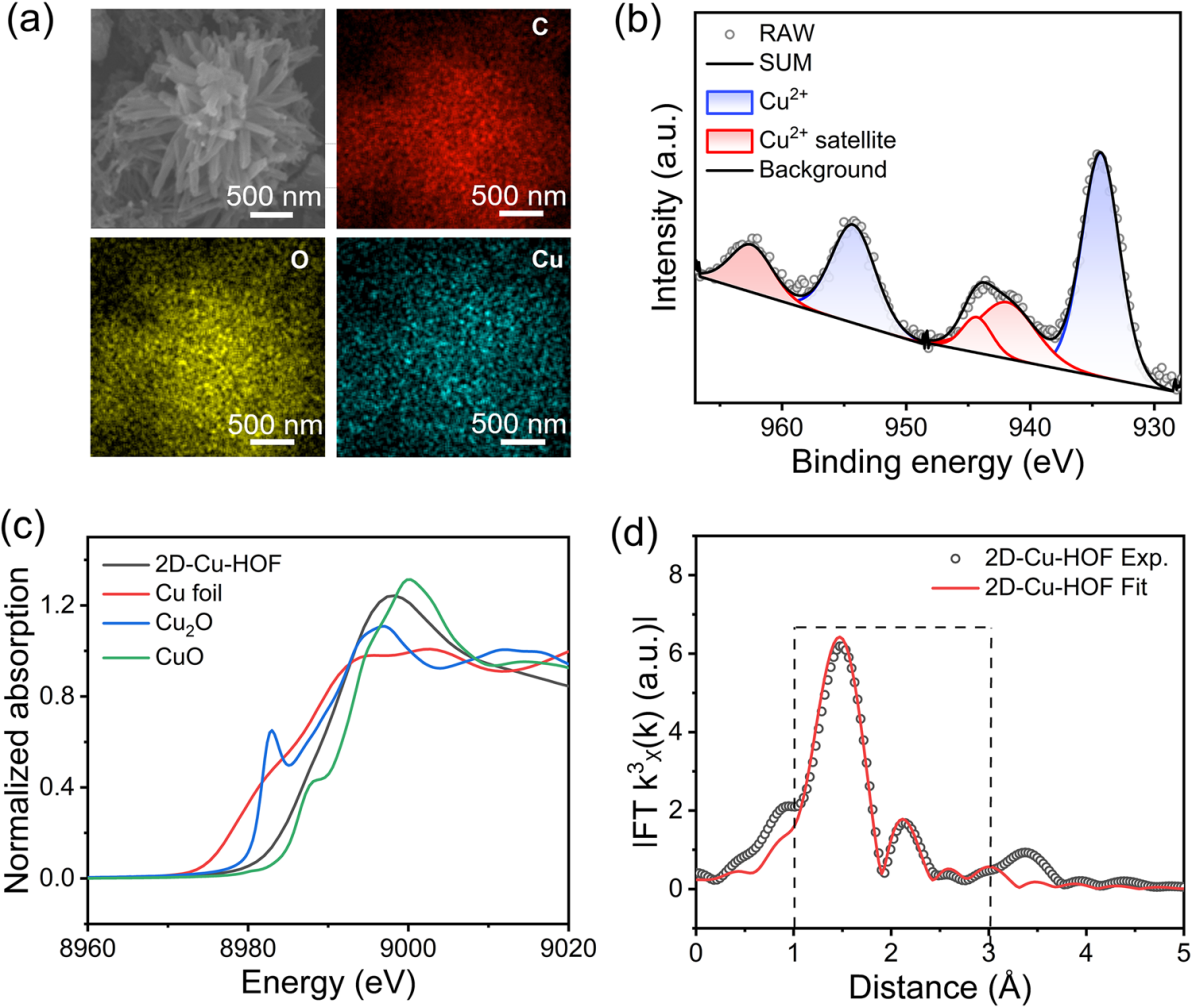
**a** EDS elemental mapping images and **b** Cu 2*p* XPS spectrum of 2D–Cu–HOF. **c** Cu *K*-edge XANES spectra of 2D–Cu–HOF, Cu foil, Cu_2_O, and CuO. **d** EXAFS fitting of Cu *K*-edge for 2D–Cu–HOF

The elemental composition of the 2D–Cu–HOF and 2D–Ni–HOF was surveyed by energy-dispersive X-ray spectrum (EDS) elemental mapping and X-ray photoelectron spectroscopy (XPS). The EDS elemental mapping analysis manifests a homogeneous distribution of C, O, and Cu/Ni elements on the nano-rod of 2D–Cu–HOF and 2D–Ni–HOF, respectively (Figs. 2a and S20, S21). Furthermore, the XPS spectra of 2D–Cu–HOF and 2D–Ni–HOF indicate the presence of C, O, and Cu/Ni elements in the material, respectively (Fig. S22). The high-resolution Cu 2*p* XPS spectrum of 2D–Cu–HOF reveals signal peaks at 934.3 and 954.3 eV, corresponding to Cu^2+^ 2*p*_3/2_ and Cu^2+^ 2*p*_1/2_, respectively. Additionally, peaks at 941.8, 944.3, and 962.5 eV are attributed to the satellite features of Cu^2+^ (Fig. 2b and Table S3). The high-resolution Ni 2*p* XPS spectrum of 2D–Ni–HOF displays two dominated peaks corresponding to Ni 2*p*_3/2_ at 855.2 eV and Ni 2*p*_1/2_ at 872.8 eV, respectively, which indicates the Ni element mainly exists as Ni^2+^ (Fig. S23, Table S4). Moreover, the high-resolution C 1*s* and O 1*s* XPS spectra of the 2D–Cu–HOF and 2D–Ni–HOF reveal the presence of two distinct oxygen species. Specifically, the C 1*s* peak at 285.5 eV and the O 1*s* peak at 532.8 eV corresponds to the phenolic C–O bond, while the C 1*s* peak at 288.2 eV and the O 1*s* peak at 531.3 eV are attributed to the quinoid C = O bond (Figs. S23 and S24). The electron paramagnetic resonance (EPR) spectra of 2D–Cu–HOF and 2D–Ni–HOF indicate a significant signal at g = 2.006 and 2.002, respectively, suggesting a semiquinone ligand structure (Fig. S25).

The valency state of coordinated metal centers in 2D–M–HOF was investigated by X-ray absorption near-edge structure spectroscopy (XANES). The XANES of 2D–Cu–HOF and 2D–Ni–HOF indicate an edge energy of 8986 eV for Cu and 8342 eV for Ni, which suggests that the valency state of M in 2D–M–HOF is close to the + 2 (Figs. 2c and S26). The extended X-ray absorption fine-structure (EXAFS) spectrum reveals the coordination environment between M^2+^ and HHCC in the 2D–M–HOF (Figs. 2d, S27, S28 and Tables S5, S6). Through fitting the EXAFS spectrum, the M–O path matches well with the experimental data, which exhibits the Cu–O and Ni–O bond with distances of 1.90 and 2.04 Å, respectively. The above characterization analysis confirms the existence of a MO_4_ structure.

The thermal stability of 2D–Cu–HOF and 2D–Ni–HOF were examined by thermogravimetric analysis (TGA) under N_2_, which is stable over 200 and 280 °C, respectively (Fig. S29). Next, the chemical stability of 2D–Cu–HOF and 2D–Ni–HOF was explored by immersing in different solvents, such as DMF, H_2_O, THF, acetone, and DCM for 3 days (Fig. S30). After the solvent treatment, the PXRD patterns of 2D–Cu–HOF and 2D–Ni–HOF display ignorable differences, which reveals their chemical robustness.

### CO_2_RR Performance of 2D–M–HOF

Electrocatalytic CO_2_RR with renewable energies has attracted considerable attention due to the possibility of converting CO_2_ into multi-carbon products, such as ethylene and ethanol [40–42]. 2D–M–HOF with active sites in a flexible framework is considered one of the ideal catalysts for enhancing the efficiency and selectivity of CO_2_RR. Firstly, the affinity between CO_2_ and 2D–M–HOF was evaluated by the CO_2_ adsorption at different temperatures. The CO_2_ uptake of 2D–Cu–HOF was found to be 46 cm^3^ g^−1^ at 273 K and 31 cm^3^ g^−1^ at 298 K, respectively, while 2D–Ni–HOF exhibited a lower CO_2_ uptake of 18 cm^3^ g^−1^ at 273 K and 13 cm^3^ g^−1^ at 298 K, respectively (Fig. S31). Meanwhile, the average adsorption heat (*Q*_st_) of 2D–Cu–HOF and 2D–Ni–HOF were calculated to be 29 and 21 kJ mol^−1^, respectively (Fig. S32). Moreover, the four-contact probe method was conducted to evaluate the electrical conductivity of 2D–Cu–HOF and 2D–Ni–HOF (Fig. S33). Due to the influence of grain boundary resistance, the electrical conductivity of 2D–Cu–HOF and 2D–Ni–HOF gradually increased when the applied pressing pressure grew from 1 to 3 MPa. The electrical conductivity of 2D–Cu–HOF and 2D–Ni–HOF is 4.39 × 10^−7^ and 1.89 × 10^−7^ S m^−1^ at 3 MPa, respectively. Doping with I_2_ is an effective method to further improve the charge transport ability of 2D–M–HOF [43–46]. As a result, the iodine-doped 2D–Cu–HOF and 2D–Ni–HOF exhibit outstanding conductivity with 1.51 × 10^−3^ and 1.86 × 10^−4^ S m^−1^ at 3 MPa, respectively (Fig. S34).

**Fig. 3:**
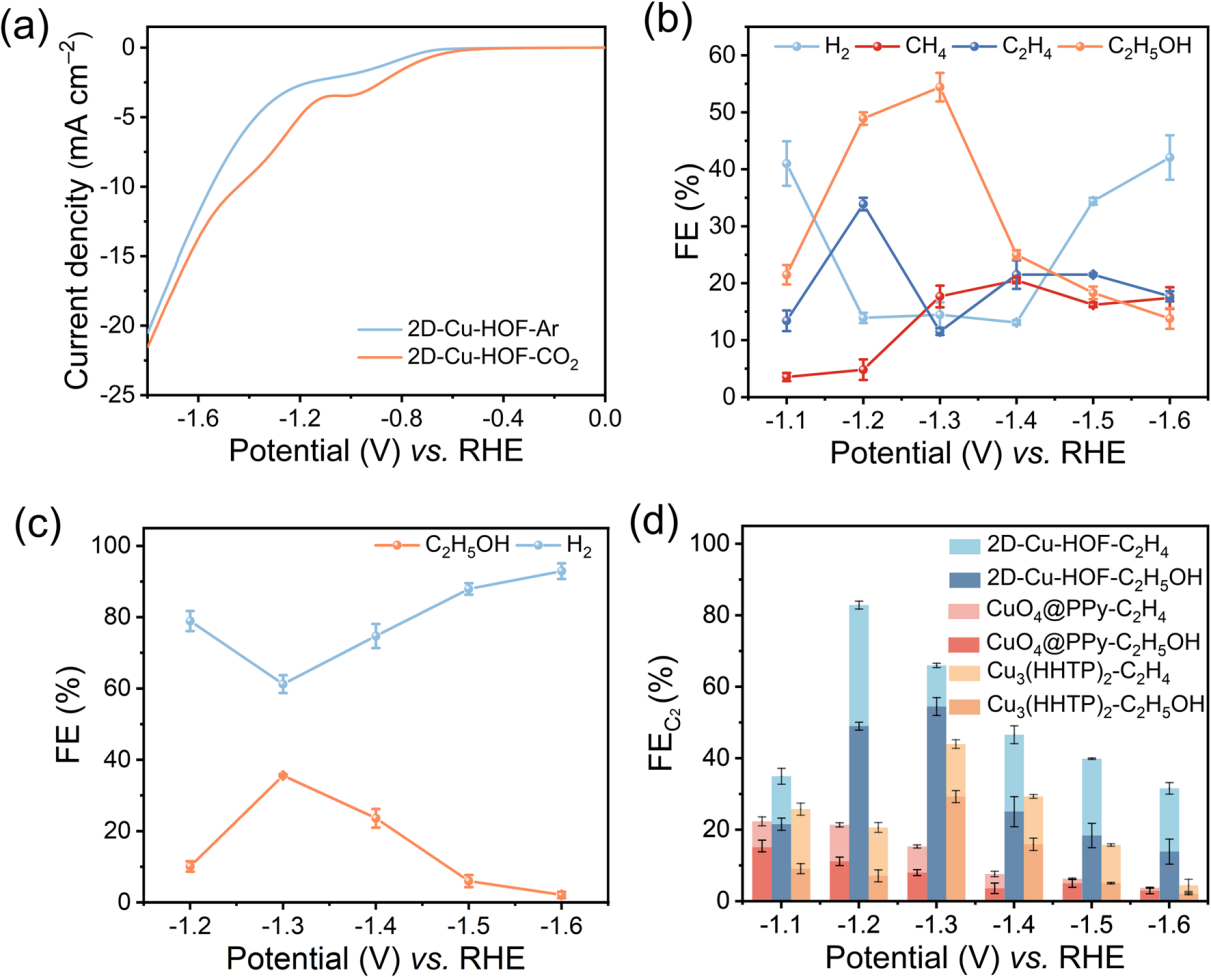
**a** LSV curves of 2D–Cu–HOF in Ar or CO_2_-saturated 0.1 M KHCO_3_ electrolytes. FEs of **b** 2D–Cu–HOF and **c** 2D–Ni–HOF at different potentials. **d** FE(C_2_) of 2D–Cu–HOF, Cu_3_(HHTP)_2_, and CuO_4_@PPy at different potentials

The electrocatalytic CO_2_RR performance of 2D–M–HOF was investigated using a typical three-electrode system with a gas-tight H-type cell in 0.1 M KHCO_3_ electrolyte at room temperature. The ink of 2D–M–HOF was loaded on a glassy carbon electrode as the working electrode. The linear sweep voltammograms (LSV) were conducted on 2D–Cu–HOF electrode in Ar or CO_2_-saturated 0.1 M KHCO_3_ aqueous electrolyte (Fig. 3a). The LSV of 2D–Cu–HOF in CO_2_-saturated electrolyte clearly demonstrates an enhanced current intensity and a more positive onset potential than those in Ar-saturated electrolyte, which can be attributed to the CO_2_ reduction process. Furthermore, the FEs of the 2D–Cu–HOF were determined at various potentials ranging from − 1.1 to − 1.6 V vs. RHE. As the potential moved to more negative values, the current density of 2D–Cu–HOF gradually increased (Fig. S35). Gas chromatography (GC) analysis revealed that H_2_, CH_4_, and C_2_H_4_ were the predominant gaseous products, while ^1^H nuclear magnetic resonance (NMR) spectroscopy detected C_2_H_5_OH as the primary liquid product (Figs. 3b and S36). The H_2_ is the dominant product for the 2D–Cu–HOF when the applied potential is lower than − 1.4 V vs. RHE or higher than − 1.2 V vs. RHE. The major C_2_ products of CO_2_RR were C_2_H_5_OH and C_2_H_4_, and the FE of C_1_ products was limited to 20% at all applied potentials. The signals of ^13^C_2_H_4_ and ^13^C_2_H_5_OH were discovered in the ^13^C isotopic labeling experiments, which confirmed that the generated C_2_H_4_ and C_2_H_5_OH during the CO_2_RR originated from the feeding CO_2_ (Fig. S37). A gradually increased FE of C_2_H_5_OH from 21.5% to 54.4% was observed when the applied potential shifted from − 1.1 to − 1.3 V vs. RHE in 2D–Cu–HOF. In the meantime, the FE of C_2_H_4_ increased from 13.4% to 33.9% when the applied potential shifted from − 1.1 to − 1.2 V vs. RHE. A maximal FE(C_2_) of 82.1% at − 1.2 V vs. RHE was obtained with 2D–Cu–HOF, which is among the highest ones in Cu-based frameworks (Table S7). Compared to 2D–Cu–HOF, 2D–Ni–HOF also exhibits a moderate FE of C_2_H_5_OH up to 35.6% at the potential of − 1.3 V vs. RHE (Figs. 3c and S38). The self-adaption interlayered sites of 2D–Ni–HOF showcase excellent catalytic activity for C_2_ products as the generation of C_2_ products is unconventional in Ni-based frameworks (Table S8). Therefore, designing the flexible 2D metal HOF is a promising strategy for obtaining C_2_ products with high selectivity during the electrocatalytic CO_2_RR process.

To investigate the influence of the flexible structure of 2D–M–HOF for electrocatalytic CO_2_RR performance, the Cu_3_(HHTP)_2_ and copper catecholate loaded on polypyrrole (CuO_4_@PPy, PPy: polypyrrole) were synthesized (Figs. S39-S42, Tables S9, S10). The Cu_3_(HHTP)_2_ has a typically rigid 2D structure with a CuO_4_ active site. On the other hand, CuO_4_@PPy represents the CuO_4_ active site arranged along a one-dimensional extended and flexible chain. As shown in Fig. 3d, 2D-Cu-HOF catalyst indicates the highest FE toward C_2_ products at − 1.2 V vs. RHE, which is approximately twice of Cu_3_(HHTP)_2_ (43.2%) and four times of CuO_4_@PPy (22.3%), respectively. Distinctly, Cu_3_(HHTP)_2_ and CuO_4_@PPy primarily generate C_1_ product (CH_4_) with FE(CH_4_) values of 35.1% at − 1.5 V vs. RHE and 39.8% at − 1.3 V vs. RHE, respectively (Figs. S43-S46). The electrochemical surface areas (ECSA) were performed to characterize the electrochemically accessible active sites of 2D–Cu–HOF, 2D–Ni–HOF, Cu_3_(HHTP)_2_, and CuO_4_@PPy. As a result, 2D–Cu–HOF possesses the largest ECSA (0.122 mF cm^−2^) among others (Fig. S47). Furthermore, 2D–Cu–HOF demonstrates the lowest Tafel slope of 227.6 mV dec^−1^ at the onset potential compared to other materials, suggesting faster electrochemical reaction kinetics (Fig. S48). Additionally, the turnover frequency of C_2_ products for 2D–Cu–HOF, 2D–Ni–HOF, Cu_3_(HHTP)_2_, and CuO_4_@PPy are calculated as 1.08 × 10^−2^, 5.83 × 10^−3^, 3.05 × 10^−3^, and 2.68 × 10^−3^ s^−1^, respectively, highlighting the remarkable intrinsic activity of 2D–Cu–HOF (Fig. S49). The durability of 2D–Cu–HOF was treated with continuous electroreduction of CO_2_ at − 1.2 V vs. RHE for at least 3 h (Fig. S50). Besides, PXRD patterns, SEM and TEM elemental mapping of 2D–Cu–HOF after electrocatalytic CO_2_RR indicate that no Cu or Cu_2_O clusters were generated during electrocatalysis, and the C, O, and Cu elements were homogeneously distributed (Figs. S51 and S52). In addition, FTIR and XPS spectra of 2D–Cu–HOF before and after electrocatalysis revealed negligible changes (Figs. S53 and S54, Table S11).

### CO_2_RR Mechanism of 2D–M–HOF

**Fig. 4:**
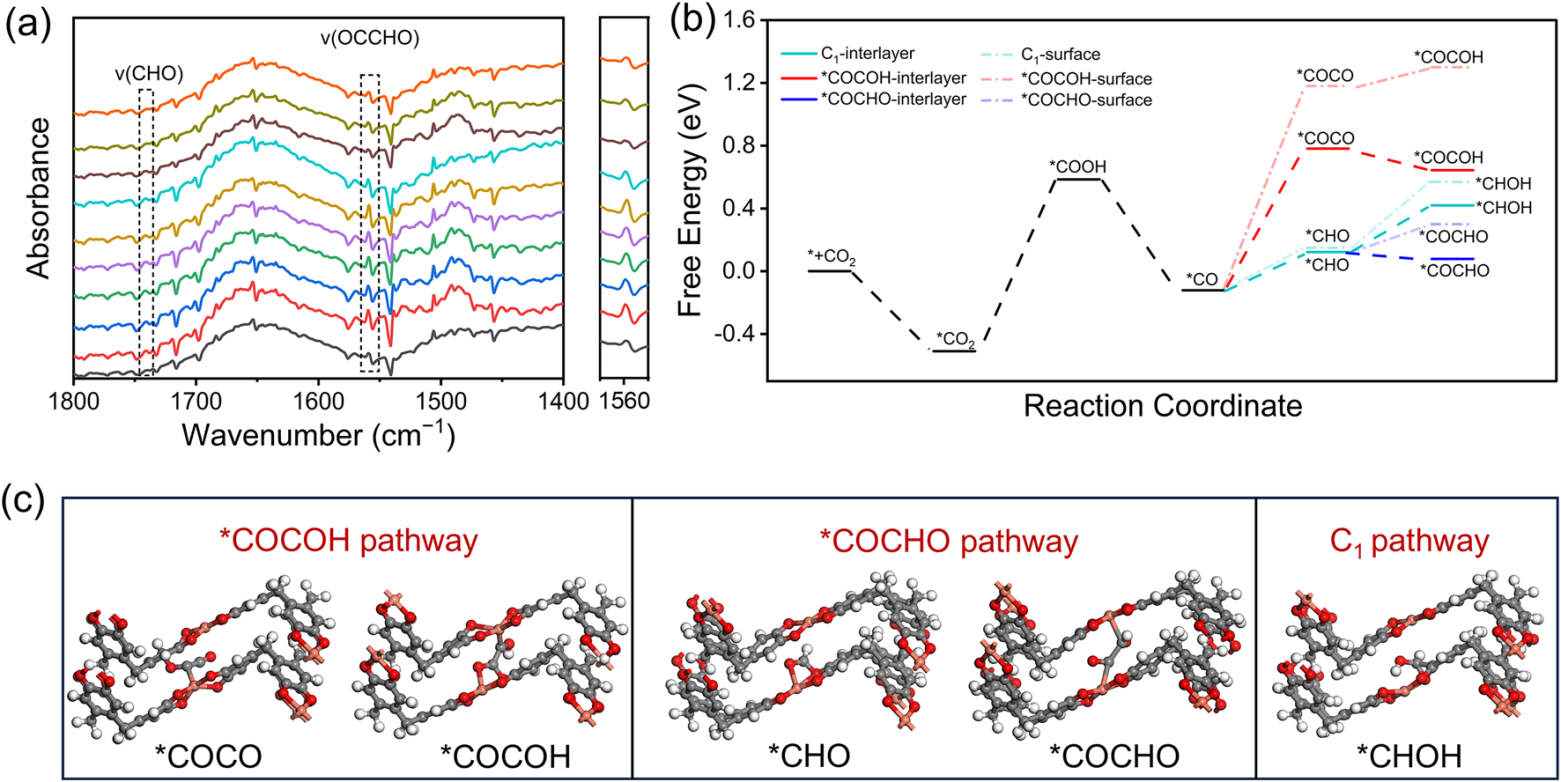
**a** Enlarged operando ATR-FTIR spectra of 2D–Cu–HOF collected at the potential from 0 to − 1.8 V vs. RHE (from bottom to top) with wavenumber from 1400 to 1800 cm^−1^. **b** Free-energy diagrams for the electrocatalytic CO_2_RR of 2D–Cu–HOF in the interlayer and surface. **c** Self-adaption interlayered sites with the intermediate structures involved in the *COCOH pathway, *COCHO pathway, and C_1_ pathway as optimized by DFT. The asterisk (*) represents chemisorbed species

The reaction intermediates during the CO_2_RR process were ascertained by the operando electrochemical attenuated total reflection Fourier transform infrared spectroscopy (ATR-FTIR). The ATR-FTIR spectrum was recorded at different potentials from 0 to − 1.8 V vs. RHE in the 0.1 M KHCO_3_ solution. As shown in Fig. S55, the stretching vibration signal at 2330 cm^−1^ attributed to adsorbed *CO_2_ was observed. The peaks observed at 1402, 1653, and 1934 cm^−1^ could be assigned to the *COO^−^ intermediate, the adsorbed H_2_O, and *CO intermediate, respectively [47]. It is worth noting that the operando ATR-FTIR of 2D–Cu–HOF revealed a band located at 1560 cm^−1^ that can be attributed to the C−O stretching of the chemisorbed *OCCHO intermediate. This intermediate is widely recognized as the key intermediate in the electrocatalytic reduction of CO_2_ to C_2_ products (Fig. 4a) [48, 49]. Furthermore, the intensity of the *OCCHO band at 1560 cm^−1^ shows an initial increase and subsequently decrease trend as the applied potential shift from 0 to − 1.8 V, aligning with the electrocatalytic results (Fig. S56). Simultaneously, the discernible presence of the characteristic *CHO band at 1740 cm^−1^ serves as compelling evidence for the formation of the *OCCHO intermediate [50].

Next, we conducted DFT calculations to reveal the reaction mechanism of CO_2_ to C_2_ products in 2D–M–HOF. A two-layer lattice of 2D–Cu–HOF was applied as a model to calculate the Gibbs free energies for the process from *CO_2_ to C−C dimerization. Figure 4b shows the Gibbs free energies diagrams for optimized configurations of intermediates along the C_1_ path or C_2_ path (*OCCO or *OCCHO). In the first step, the CO_2_ molecules are chemisorbed to the Cu active sites of 2D–Cu–HOF with an energy release of 0.51 eV. Afterward, an H^+^/e^−^ attacks the oxygen atom to form *COOH intermediate involving free energy increasing with a value of 1.10 eV. Subsequently, *CO intermediate is formed owing to eliminating an H_2_O molecule at the oxygen atom with a decreased energy of 0.71 eV. In the next step, the three possible reaction pathways are considered for the transformation of the reaction intermediate in the electrocatalytic CO_2_RR process. Compared to the direct *CO−CO dimerization (0.94 eV), the formation of *CHO intermediate (0.24 eV) suggests a relatively lower energy increase. Furthermore, it’s worth noting that the formation of *OCCHO through *CHO with *CO dimerization is more energetically favorable compared with the C_1_ reaction path from *CHO to *CHOH (− 0.04 vs. 0.30 eV). Moreover, the Gibbs free energy of the key intermediates on the surface of 2D–Cu–HOF is higher than that of interlayered sites between adjacent layers. This suggests that the undulated structural characteristic of 2D–Cu–HOF enables the self-adaption interlayered sites to possess certain degrees of freedom in adjusting their distance during the process of C−C coupling (Figs. 4c and S57). This feature provides sufficient space between adjacent layers of 2D–Cu–HOF can accommodate the intermediate of the electrocatalytic CO_2_RR reaction process and adjust to a suitable distance for the coadsorption of the C−C coupled intermediate.

## Conclusions

In conclusion, we developed two undulated 2D–M–HOF (2D–Cu–HOF and 2D–Ni–HOF) with self-adaption interlayered sites based on the HHCC ligand. The single-crystal structure analysis of 2D–Cu–HOF utilizing cRED demonstrated CuO_4_ nodes and an undulated 2D hydrogen-bonding network with the π-π interaction. Due to the flexible framework structure of 2D–M–HOF, the self-adaption interlayered sites are formed, facilitating the coadsorption and activating of C–C coupling intermediates. The 2D–Cu–HOF catalyst exhibits high selectivity toward C_2_ product with an FE up to 82.1% at − 1.2 V vs. RHE. Notably, the 2D–Ni–HOF also showcases an outstanding selectivity to C_2_H_5_OH with an FE of 35.6%. The reaction mechanism during the CO_2_RR process was investigated both experimentally and theoretically, in which the CO_2_-to-*COCHO path shows the lowest reaction energy barrier. The *COCHO intermediates were detected in the ATR-FTIR spectrum. This work opens up a new avenue for the structure design of HOFs with excellent performance in electrocatalysis.

## Supplementary Information

Below is the link to the electronic supplementary material.

Supplementary file1 (DOCX 27964 KB)
