# Supplementary material for: 2D Undulated Metal Hydrogen-Bonded Organic Frameworks with Self-Adaption Interlayered Sites for Highly Efficient C–C Coupling in the Electrocatalytic CO2 Reduction
Source: Nanomicro Lett. 2025 Feb 24;17:162. doi: 10.1007/s40820-025-01679-3 (PMC11850663; doi:10.1007/s40820-025-01679-3)
Supplement: Supplementary file 1 — Supplementary file1 (DOCX 27964 KB) [file 40820_2025_1679_MOESM1_ESM.docx]

# Supporting Information for

**2D Undulated Metal** **Hydrogen-Bonded Organic Frameworks with Self-Adaption Interlayered Sites for Highly Efficient C–C Coupling** **in the Electrocatalytic CO_2_ Reduction**

Jianning Lv^1^, Wenrui Li^1^, Shuai Li^1, 4^, Shuo Xu^1^, Zunhang Lv^1^, Zhejiaji Zhu^1^, Lu Dai^1^, Bo Wang^1, 2^ and Pengfei Li^1, 3,^ *

^1^ Key Laboratory of Cluster Science Ministry of Education, Beijing Key Laboratory of Photoelectronic/Electrophotonic, School of Chemistry and Chemical Engineering, Beijing Institute of Technology, No. 5, South Street, Zhongguancun, Haidian District, Beijing 100081, P. R. China

^2^ Advanced Technology Research Institute (Ji’nan), Beijing Institute of Technology, Ji’nan, Shandong 250300, P. R. China

^3^ Advanced Research Institute of Multidisciplinary Science, Beijing Institute of Technology (Zhuhai), No.6, Jinfeng Road, Tangjiawan, Zhuhai 519088, P. R. China

^4^ Petrochina Petrochemical Research Institute, No.7, Kunlun Street, Changping District, Beijing 102206, P. R. China

*Corresponding author. E-mail: [lipengfei@bit.edu.cn](mailto:lipengfei@bit.edu.cn) (Pengfei Li)

**S1 Experimental Details**

**S1.1 Materials synthesis**


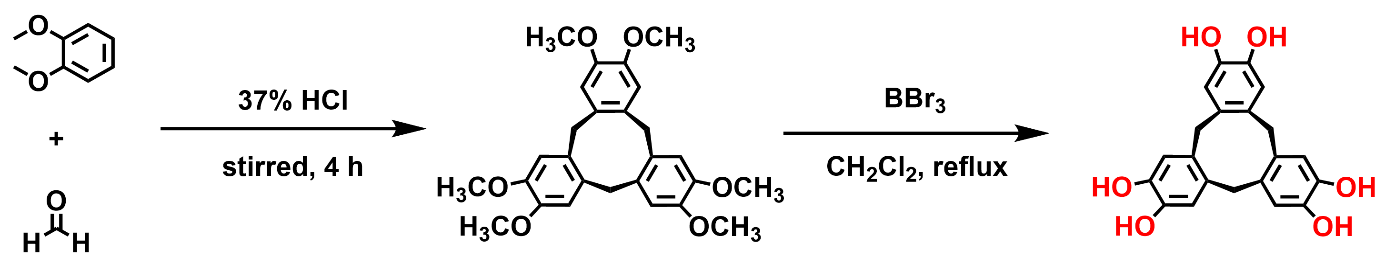


**Scheme S1** The synthetic route toward hexahydroxyl cyclotricatechylene

***S1.1.1 The synthesis of hexamethoxy cyclotriveratrylene (HMCC)***

According to the previous literature [S1], aqueous HCl (37%, 54 mL) was placed in a 250 mL three-neck flask and cooled to 0 ^o^C by using an ice water bath. The aqueous formaldehyde (38%, 40 mL) was added carefully into the above solvent under stirring. Then, veratrole (9.2 mL) was added portion-wise to a stirred mixture, and the resultant mixture was stirred for 30 min at 0 ^o^C. Next, the reaction temperature was adjusted from 0 ^o^C to room temperature and stirred for 4 h. The white product was filtered, and washed with deionized water several times until the filtrate was neutral. The resulting powder was purified by recrystallization in toluene, then dried at 100 ^o^C under a vacuum for 12 h to obtain white crystals of HMCC in 92% yield. ^1^H NMR (400 MHz, CDCl_3_) *δ* (ppm): 3.84 (18H, s), 3.58 (3H, d, *J* = 13.8 Hz), 4.80 (3H, d, *J* = 13.73 Hz), 6.83 (6H, s). ^13^C NMR (100 MHz, CDCl_3_) *δ* (ppm): 147.75, 131.79, 113.15, 56.05, 36.54.

***S1.1.2 The synthesis of*** ***hexahydroxyl cyclotricatechylene (HHCC)***

According to the previous literature [S2], in a three-neck 100 mL round bottom flask, a solution of HMCC (2.52 g, 5 mmol) in dry DCM was cooled to 0 °C under an Ar atmosphere. To this stirred solution, BBr_3_ (3.3 mL, 35 mmol) was added. The solution was removed from the cooling bath and the purple-colored reaction mixture was brought to room temperature and stirred for a further 15 min. Then it was refluxed for 12 h. After this time, the reaction mixture was slowly cooled to 0 °C by an ice water bath. It was then quenched by the slow addition of ice-cold water (50 mL). The resulting slurry was filtered and the residue was washed with water (200 mL) and acetonitrile (10 mL). The crude wet solid was recrystallized from ethanol to obtain brown crystals of HHCC in 87% yield. ^1^H NMR (400 MHz, DMSO-*d*_6_) *δ* (ppm): 8.52 (6H, s), 6.65 (6H, s), 4.49 (3H, d, *J* = 13.4 Hz), 3.21 (3H, d, *J* = 13.6 Hz). ^13^C NMR (100 MHz, DMSO-*d*_6_) *δ* (ppm): 143.91, 131.23, 117.15, 35.49.

***S1.1.3 The general synthetical procedure for 2D-Cu-HOF and 2D-Ni-HOF***

Metal salt (0.0324 mmol) and HHCC (0.0217 mmol) were dissolved in a mixed solvent in a 4 mL glass vial. The vial was sonicated for 30 minutes. Then a base was added to the above solution. The vial was capped and heated in an isothermal oven. When the vial was cooled to room temperature, the mixture was centrifugated, and the deposit was washed with H_2_O (10 mL × 5) and acetone (10 mL × 3), successively. Finally, the obtained solid was dried at 60 ^o^C under a vacuum for 12 h.

***S1.1.4 The optimization of bases***

All the synthetical conditions are the same as the general synthetical procedure, except that 1.5 mL of H_2_O/MeOH (v/v = 2:1) was added as the mixed solvent and 500 µL TEA, 500 µL NH_3_·H_2_O (25 wt%), 200 µL TBAH (1 M), 200 µL EDA, or 200 µL of 6 M NaOH was added as the base, respectively.

***S1.1.5 The optimization of solvent combinations***

All the synthetical conditions are the same as the general synthetical procedure, except that 1.5 mL of H_2_O/MeOH, H_2_O/1,4-dioxane, H_2_O/DMSO, H_2_O/DMAc, or H_2_O/DMF (v/v = 2:1) was added as the mixed solvent and 50 µL of NH_3_·H_2_O (25 wt%) was added as the base.

***S1.1.6 The optimization of the amount of base***

All the synthetical conditions are the same as the general synthetical procedure, except that 1.5 mL of H_2_O/MeOH (v/v = 2:1) was added as the mixed solvent and 50, 200, or 500 µL of NH_3_·H_2_O (25 wt%) was added as the base, respectively.

***S1.1.7 The optimization of solvent ratios***

All the synthetical conditions are the same as the general synthetical procedure, except that 1.5 mL of H_2_O/MeOH (v/v = 1.5:0.5, 1.6:0.4, 1.7:0.3, 1.8:0.2, or 1.9:0.1) was added as the mixed solvent and 50 µL of NH_3_·H_2_O (25 wt%) was added as the base.

***S1.1.8 The optimization of temperature and reaction time***

All the synthetical conditions are the same as the general synthetical procedure, except that 60, 80, or 100 ^o^C as the reaction temperature and 3, 5, 7, or 10 days as the reaction time.

**S1.2 Characterization**

^1^H NMR and ^13^C NMR spectra were measured on a Bruker ARX-400 NMR spectrometer with tetramethylsilane (TMS) as the internal standard. Powder X-ray diffraction (PXRD) was recorded on a Rigaku MiniFlex 600 diffractometer using a Cu Kα X-ray source with 40 kV voltage and 15 mA current. Fourier transform infrared (FT-IR) spectra were performed on Bruker ALPHA spectrometer in the range of 400-4000 cm^-1^. The content of Cu was measured by inductively coupled plasma-optical emission spectroscopy (ICP-OES). The elemental analysis (EA) was performed on an Elementar UNICUBE. Thermogravimetric analysis (TGA) was conducted on a NETZSCH Proteus STA 449F5 analyzer at a heating rate of 10 ^o^C/min in the temperature range of 35-800 ^o^C under an N_2_ atmosphere. Scanning electron microscopy (SEM) images were obtained from a JEOL model JSM-7500F scanning electron microscope. Transmission electron microscopy (TEM) images were collected on JEOL model JM-2100 microscope. X-ray photoelectron spectroscopy (XPS) was performed by using Thermo scientific ESCALAB 250Xi with Al Kα radiation. Electron paramagnetic resonance spectroscopy (EPR) was conducted on a Bruker EMX plus with a sweep width of 1500 G and a center field of 3500 G. The X-ray absorption spectra (XAS) were collected at the Singapore Synchrotron Light Source (SSLS) center, where a pair of channel-cut Si (111) crystals was used in the monochromator. The electronic beam energy of 700 MeV with an average electron current of below 200 mA. The continuous rotation electron diffraction was collected using the JEM-2100 Plus transmission electron microscope produced by NEC Corporation. The microscope operated at an acceleration voltage of 200 kV, with a wavelength of 0.0025079 nm, and was outfitted with a MerelinEM high-speed direct electron camera. The experiments were conducted utilizing a Fischione 2550 refrigerated transmission rod at an ambient temperature of 77 K.

***S1.2.1 The measurement of N_2_ and CO_2_ adsorption isotherms***

The gas adsorption measurements were performed on a Quantachrome Instrument Autosorb-iQ with N_2_ at 77 K and CO_2_ at 273 K and 298 K, respectively. The sample was immersed in anhydrous acetone (10 mL) for 2 days, during which the solvent was decanted and replaced with fresh solvent 3 times per day. Then the product was separated by centrifugation and dried at 60 ^o^C under vacuum for 12 h. Before gas adsorption measurement, the product was evacuated at 120 °C for 12 h under a vacuum. The Brunauer–Emmett–Teller (BET) surface area was evaluated in the relative pressure range from 0.05 to 0.40. The pore size distribution was determined by non-local density functional theory.

***S1.2.2 The measurement of electrical conductivity***

40 mg of sample was placed into a standard die with a diameter of 10 mm and was pressed at pressures of 1, 2, and 3 MPa for 30 s to form the pellet, respectively. Electrical conductivity measurements were performed at 298 K and a relative humidity of 45%. The electrical conductivity was obtained by an ST2742B resistivity meter with a four-point probe.

***S1.2.3 XAFS data processing***

The acquired EXAFS data were processed according to the standard procedures using the Athena and Artemis software packages.[S3] The EXAFS spectra were obtained by subtracting the post-edge background from the overall absorption and then normalizing concerning the edge-jump step. Subsequently, the χ(k) data were Fourier transformed to real space to separate the EXAFS contributions from different coordination shells. To obtain the quantitative structural parameters around central atoms, least-squares curve parameter fitting was performed using the ARTEMIS.

**S1.3 CO_2_RR performance evaluation**

The electrocatalytic reduction of CO_2_ was conducted in an H-type cell with two compartments separated by a Nafion-117 membrane and tested by an electrochemical workstation (CHI 760E). Each compartment contained CO_2_-saturated KHCO_3_ electrolyte (0.1 M, 30 mL). The linear sweep voltammograms and electrolysis were performed in a three-electrode system using Pt foil as the counter electrode and Ag/AgCl electrode as the reference electrode. The working electrode was prepared as follows: 2 mg of catalyst and 20 μL of 5 wt% Nafion were dispersed in a mixed solution of isopropanol (0.15 mL) and H_2_O (0.05 mL). The mixed ink was sonicated for 10 min. Then 5 μL of the catalyst ink was dropped onto a glassy carbon (GC) disk with a diameter of 5 mm to form the working electrode. All gas phase products were detected by gas chromatography (GC, Shimadzu) with TCD and FID detectors. All liquid products were detected by ^1^H NMR spectroscopy with water signal suppression. 0.5 mL of electrolyte was mixed with 0.1 mL of D_2_O and dimethyl sulfoxide solution (DMSO, 0.25 mmol/L), wherein DMSO was used as an internal standard. Before electrolysis, pure CO_2_ gas or Ar gas was bubbled into a 0.1 M KHCO_3_ aqueous solution with stirring for 30 min to obtain CO_2_ or Ar-saturated KHCO_3_ electrolyte. During the whole electrochemical test, CO_2_ was kept purging in the electrolyte. The Faradaic efficiency of the different products was calculated by the equation:

$$FE = \frac{\alpha\times n \times F}{i \times t}$$

(Where *α* is the number of electrons transferred, *n* is the total amount of the gas product (in moles), *F* is the Faraday constant, *i* is current, *t* is the running time)

The turnover frequency (TOF) was calculated by the equation:

$$TOF = \frac{i \times FE}{N \times F \times n_{tot}}$$

Where *i* is the current, FE is the Faradaic efficiency for the product, N is the number of electrons in the half-reaction (N = 12 for the CO_2_ to C_2_H_4_ and C_2_H_5_OH conversion), F is the Faraday constant, and n_tot_ is the total moles of catalyst used in the electrolysis.

**S1.4 DFT computational details**

All the calculations are performed in the framework of the density functional theory with the projector augmented plane-wave method, as implemented in the Vienna ab initio simulation package. [S4, S5] The generalized gradient approximation proposed by Perdew-Burke-Ernzerhof (PBE) is selected for the exchange-correlation potential. [S6] The cut-off energy for the plane wave is set to 450 eV. The energy criterion is set to 10^−5^ eV in the iterative solution of the Kohn-Sham equation. All the structures are relaxed until the residual forces on the atoms have declined to less than 0.02 eV/Å. To avoid interlaminar interactions, a vacuum spacing of 20 Å is applied perpendicular to the slab.

# S2 Figures and Tables


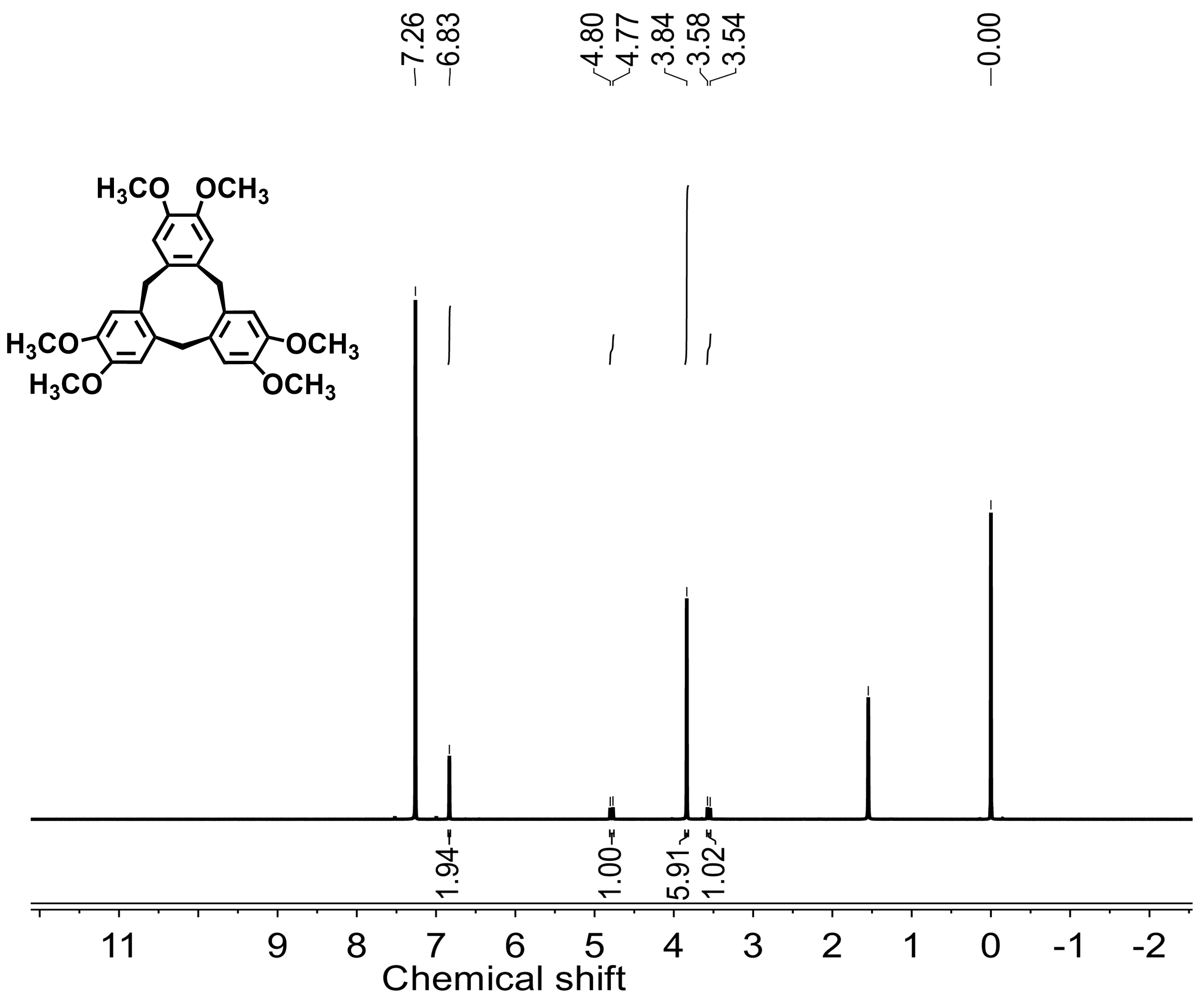


**Fig. S1** ^1^H NMR spectrum of HMCC (CDCl_3_, 400 MHz)


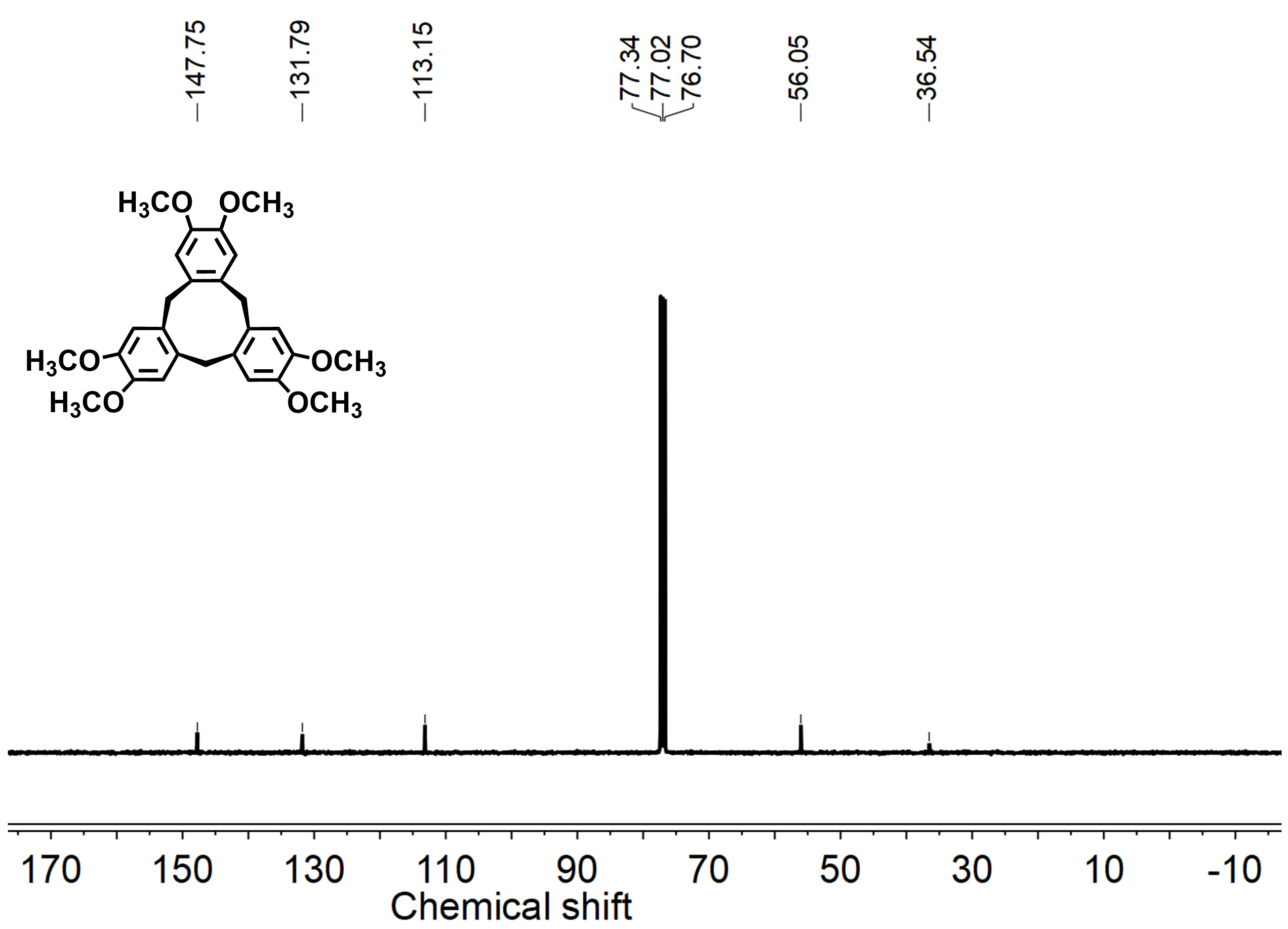


Fig. S2 ^13^C NMR spectrum of HMCC (CDCl_3_, 400 MHz)


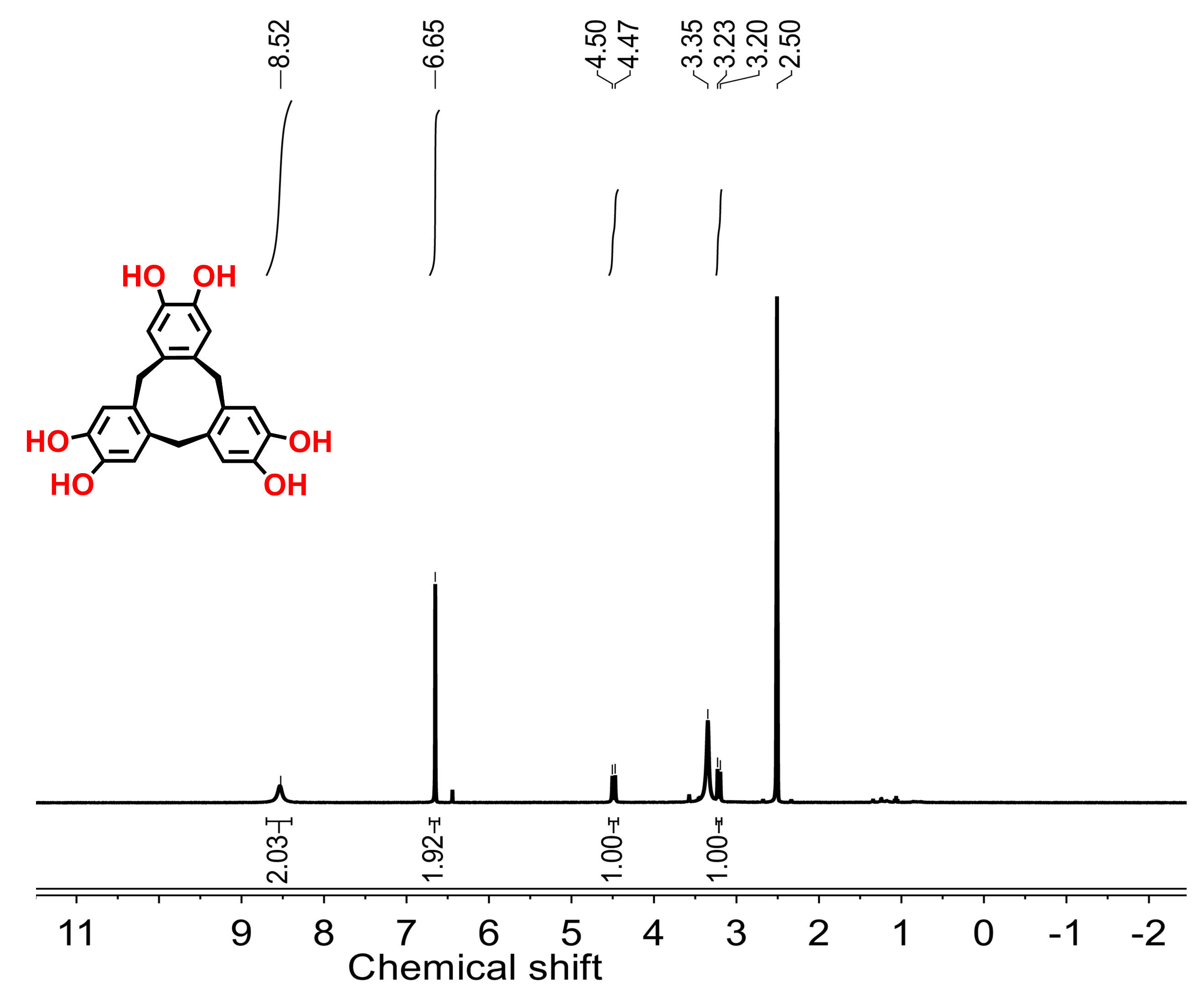


**Fig. S3** ^1^H NMR spectrum of HHCC (DMSO-*d*_6_, 400 MHz)


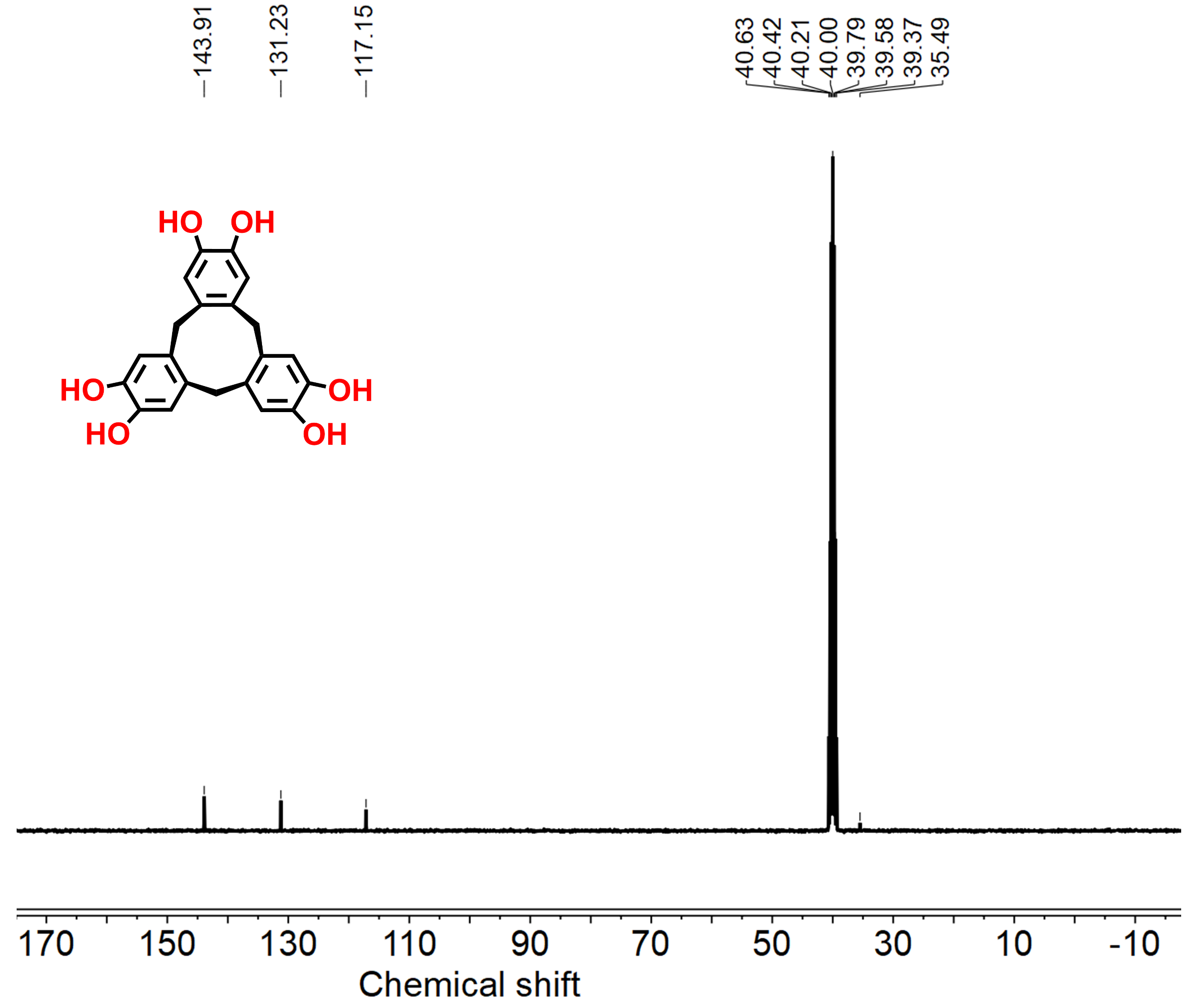


**Fig. S4** ^13^C NMR spectrum of HHCC (DMSO-*d*_6_, 400 MHz)


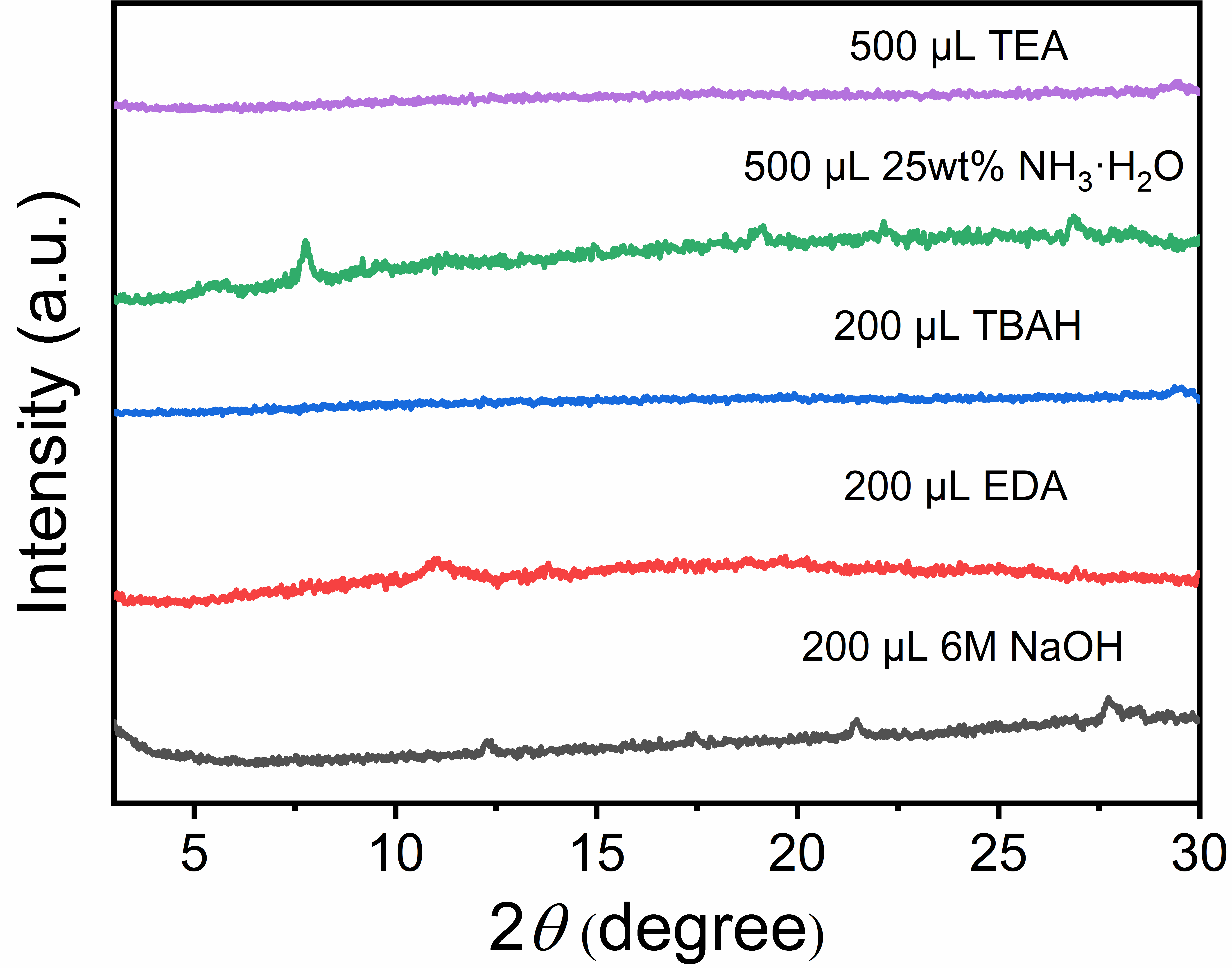


**Fig. S5** PXRD patterns of 2D-Cu-HOF synthesized in H_2_O/MeOH with the addition of different bases


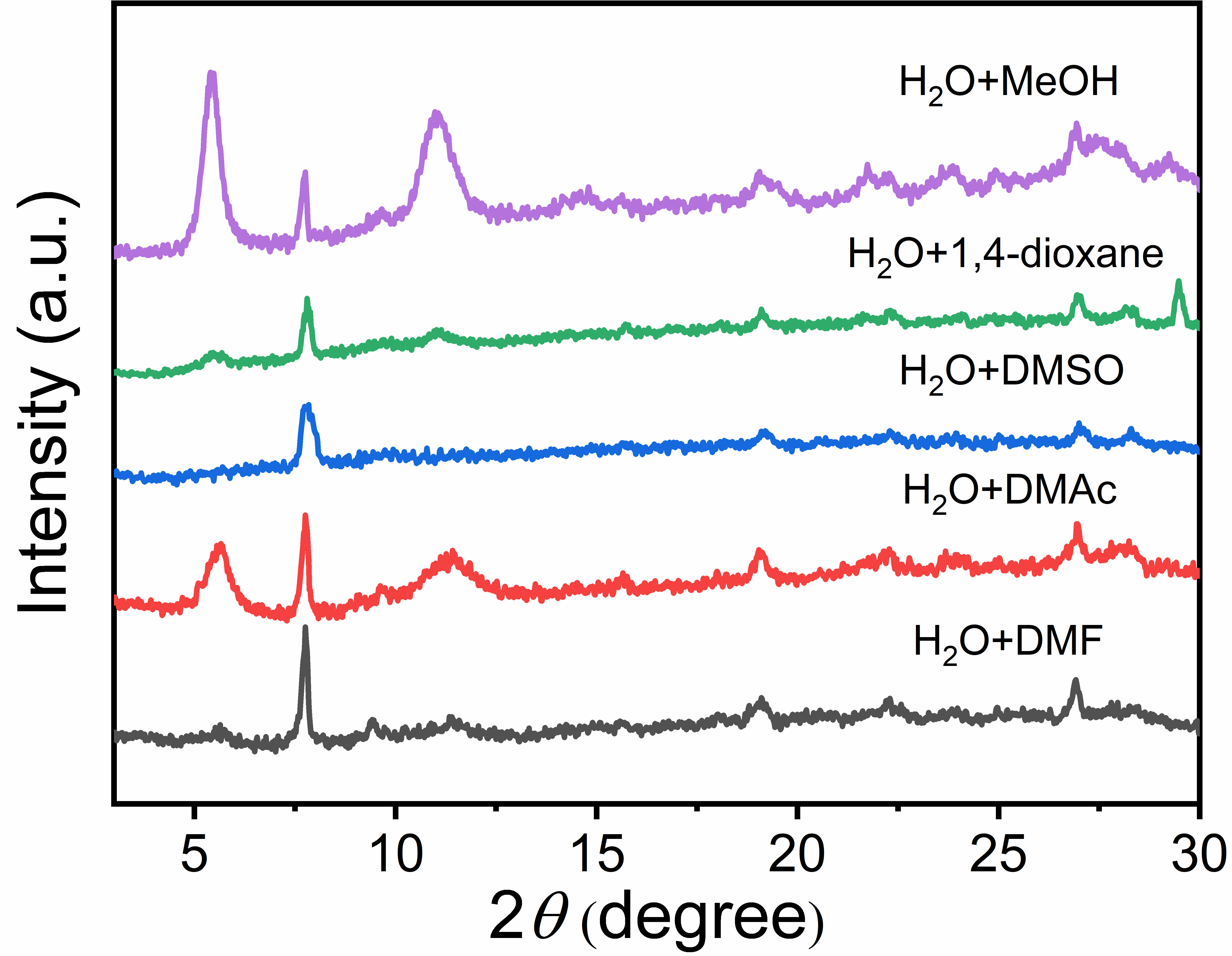


**Fig. S6** PXRD patterns of 2D-Cu-HOF synthesized with different solvents


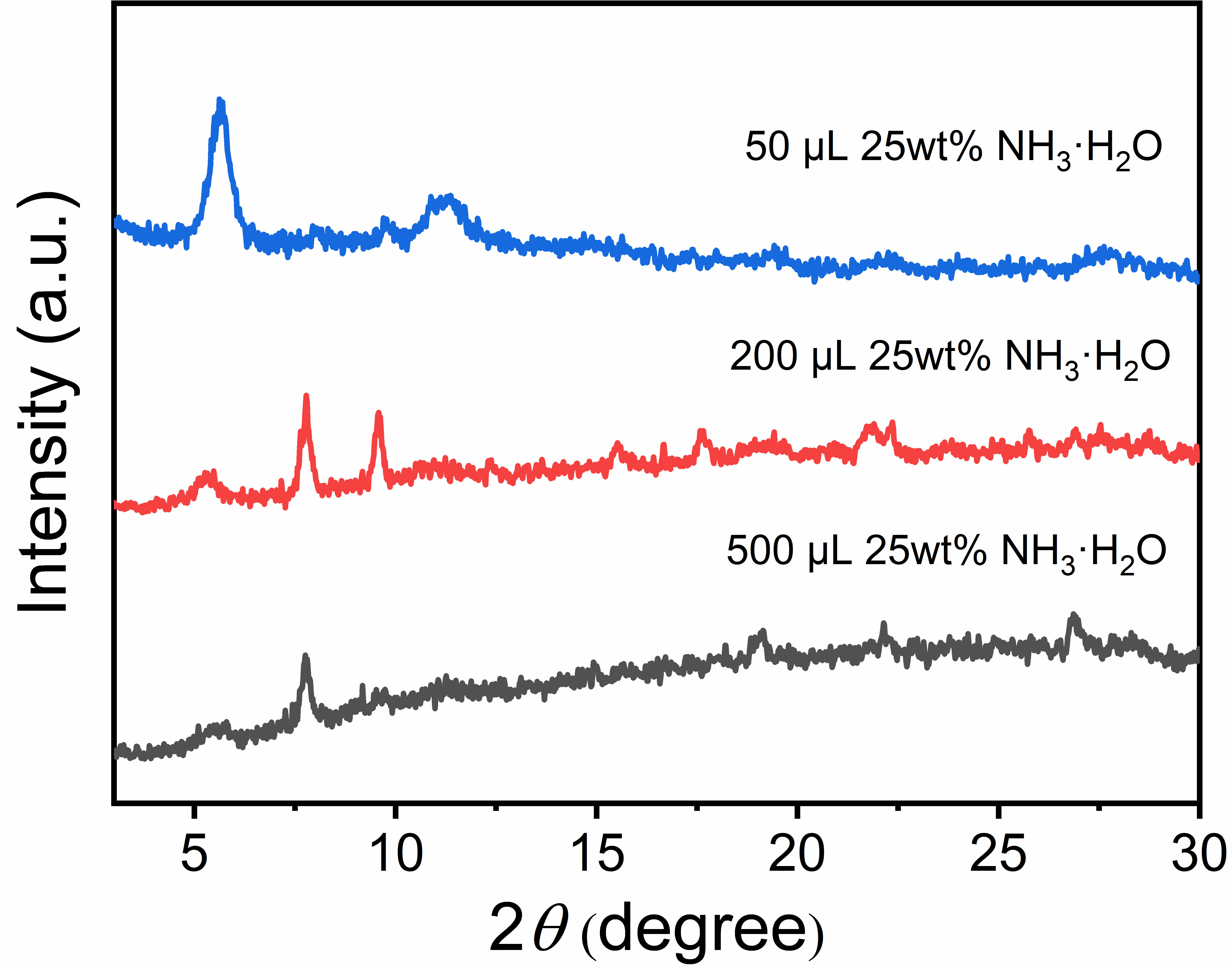


**Fig. S7** PXRD patterns of 2D-Cu-HOF synthesized in H_2_O/MeOH with the addition of different amounts of 25 wt% NH_3_·H_2_O





**Fig. S8** PXRD patterns of 2D-Cu-HOF synthesized with different ratios of H_2_O/MeOH


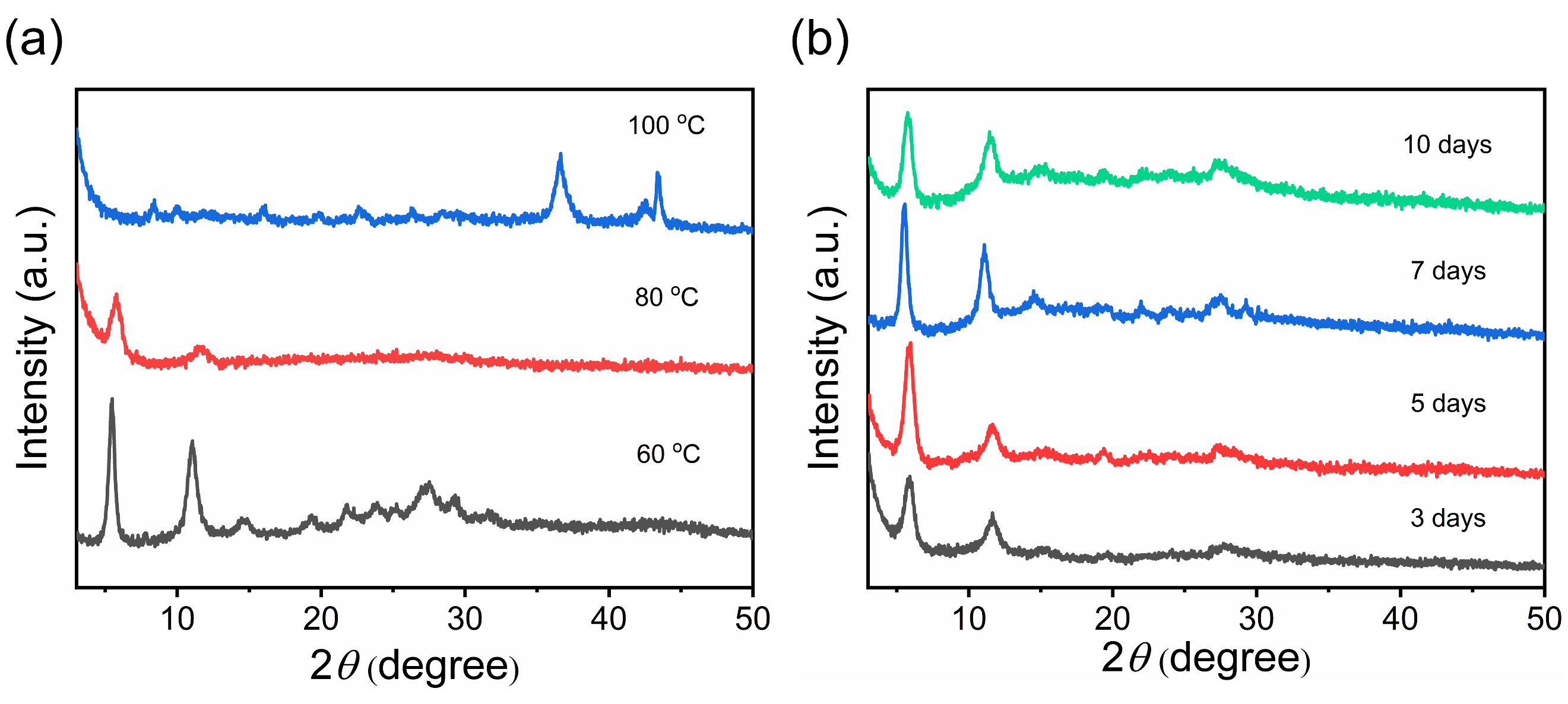


**Fig. S9** PXRD patterns of 2D-Cu-HOF synthesized with different reaction temperature (**a**) and time (**b**)


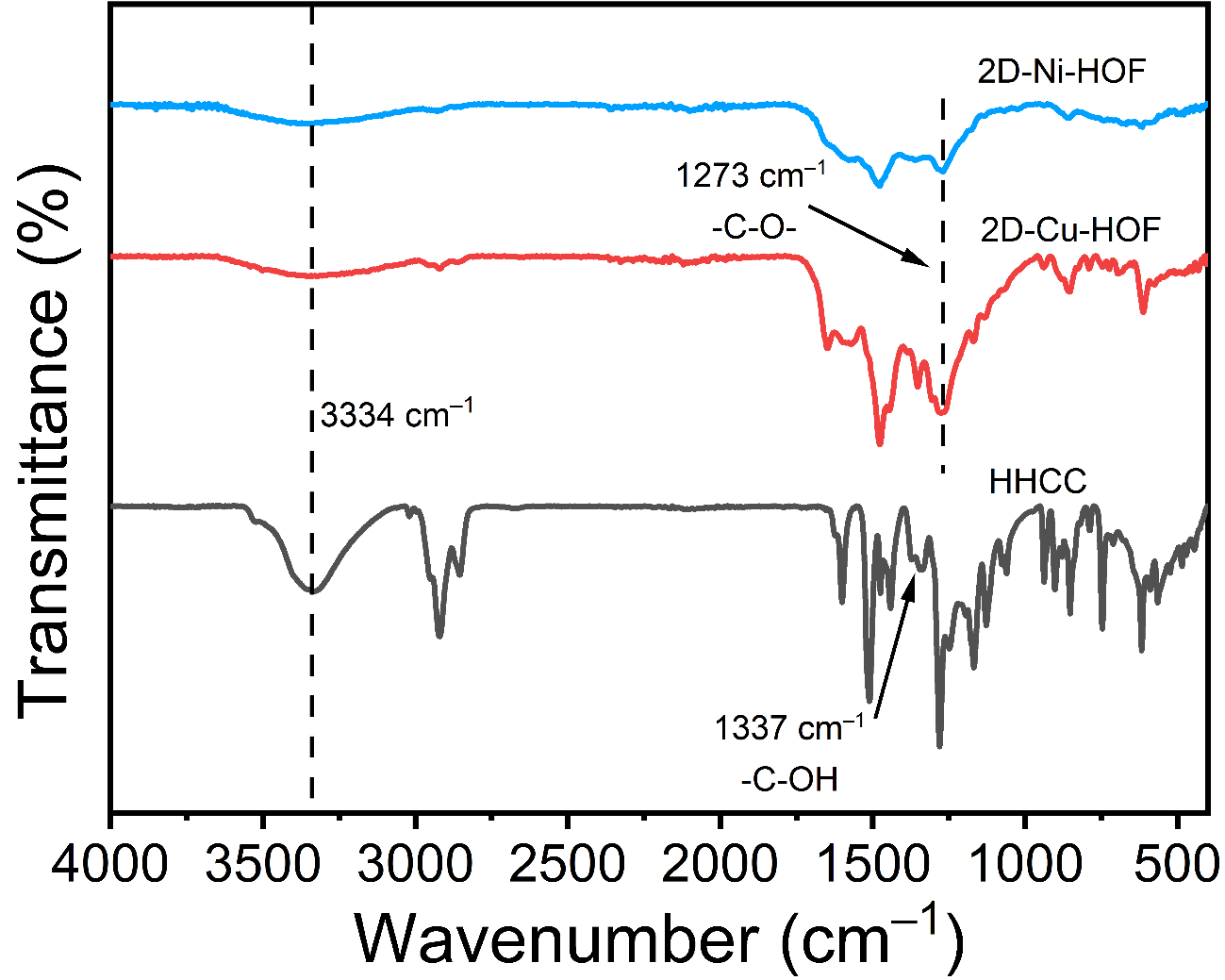


**Fig. S10** FT-IR spectra of HHCC (black), 2D-Cu-HOF (red) and 2D-Ni-HOF (blue)


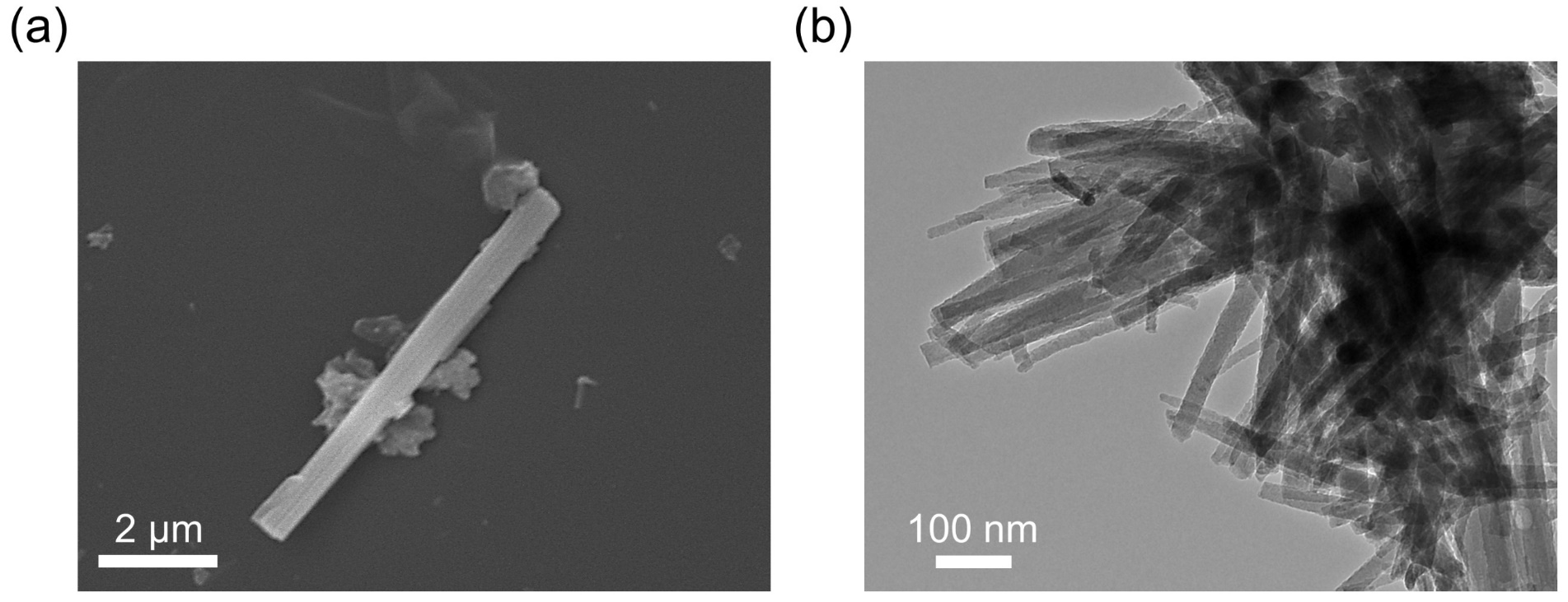


**Fig. S11** SEM (**a**) and TEM (**b**) images of 2D-Cu-HOF


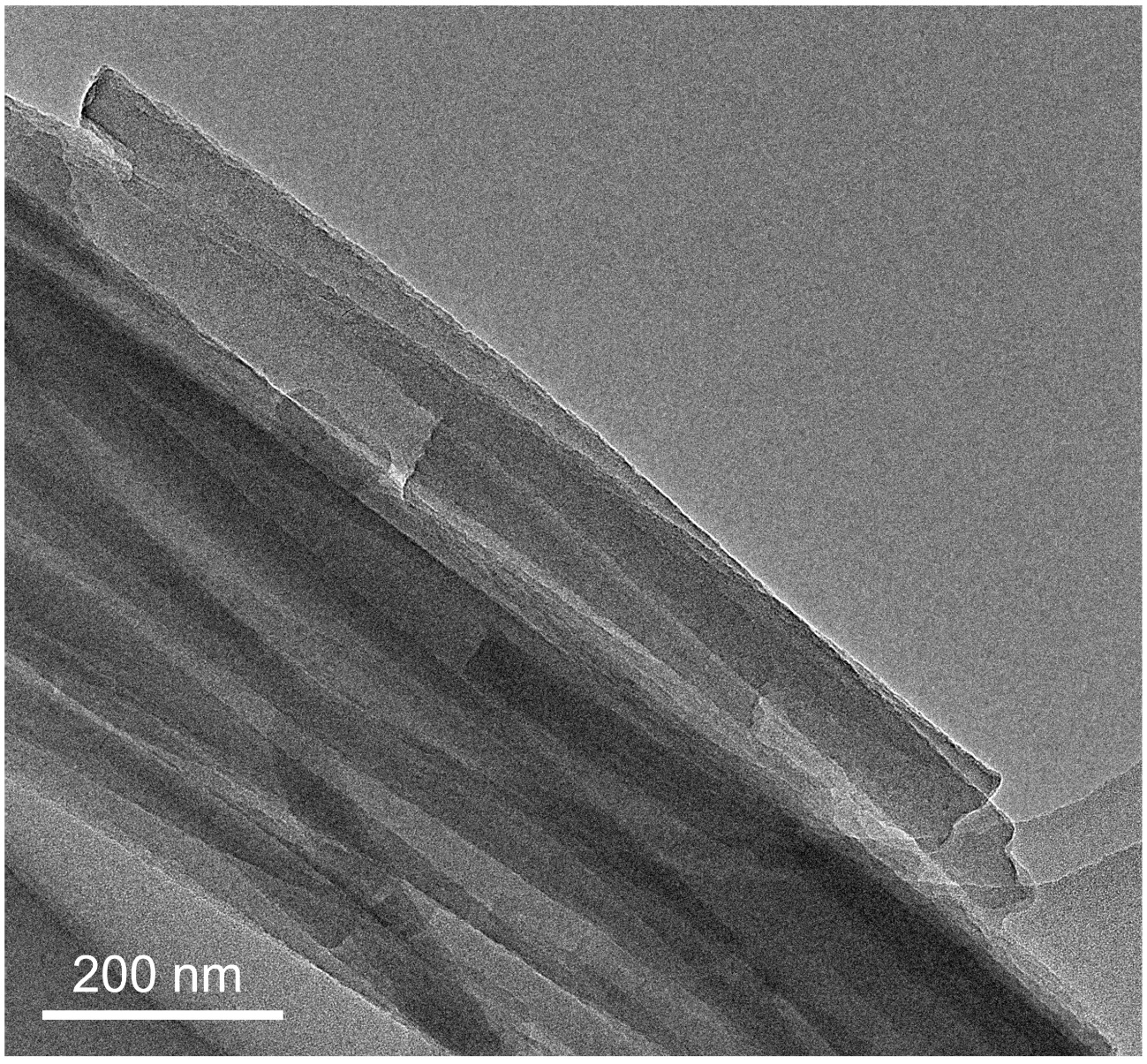


**Fig. S12** TEM image of 2D-Ni-HOF


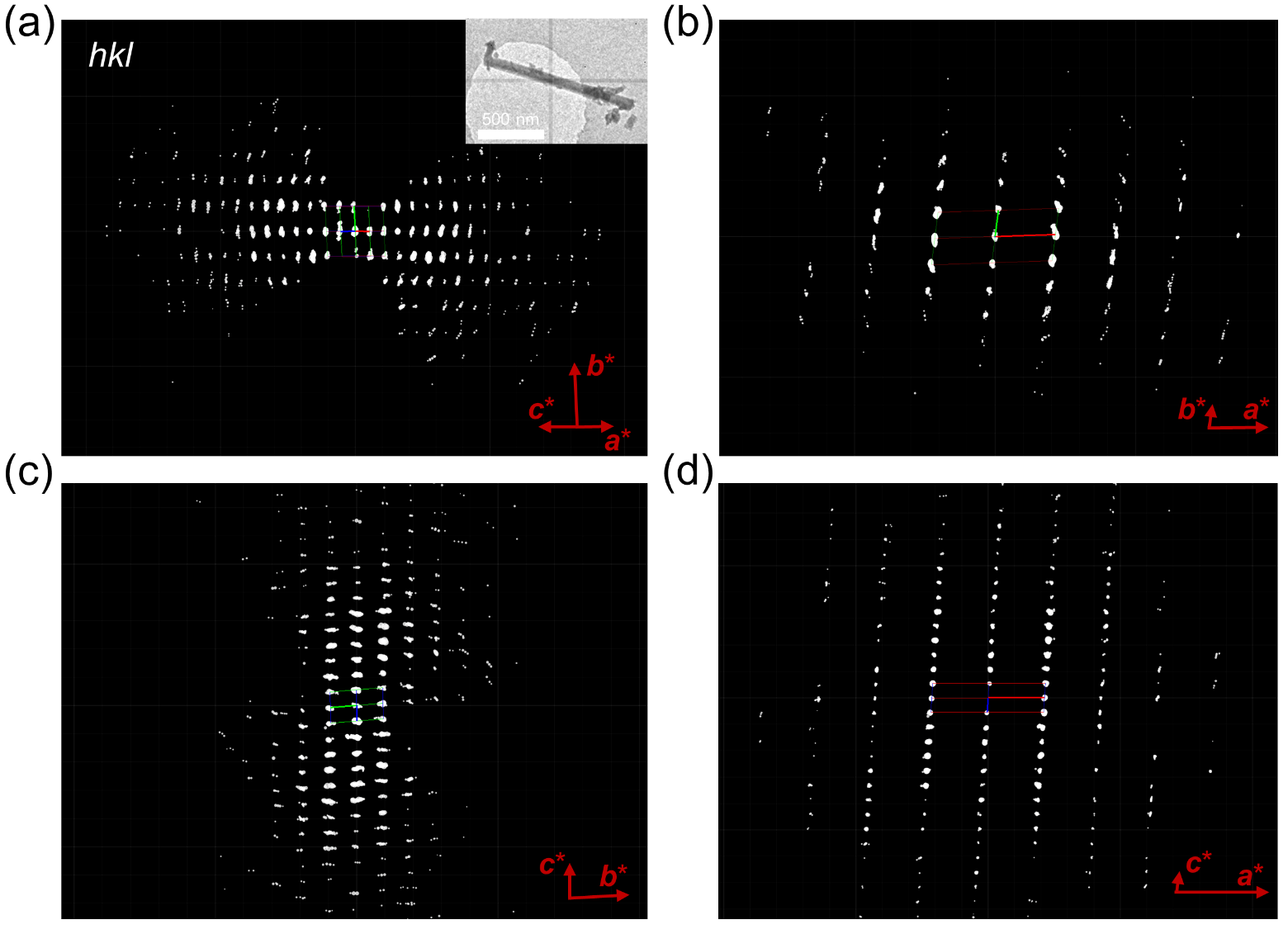


**Fig. S13** 3D cRED data for 2D-Cu-HOF. (**a**) Overview of the 3D reciprocal lattice, (**b**) hk0 slice cut from the 3D reciprocal lattice, (**c**) 0kl slice cut from the 3D reciprocal lattice, (**d**) h0l slice cut from the 3D reciprocal lattice


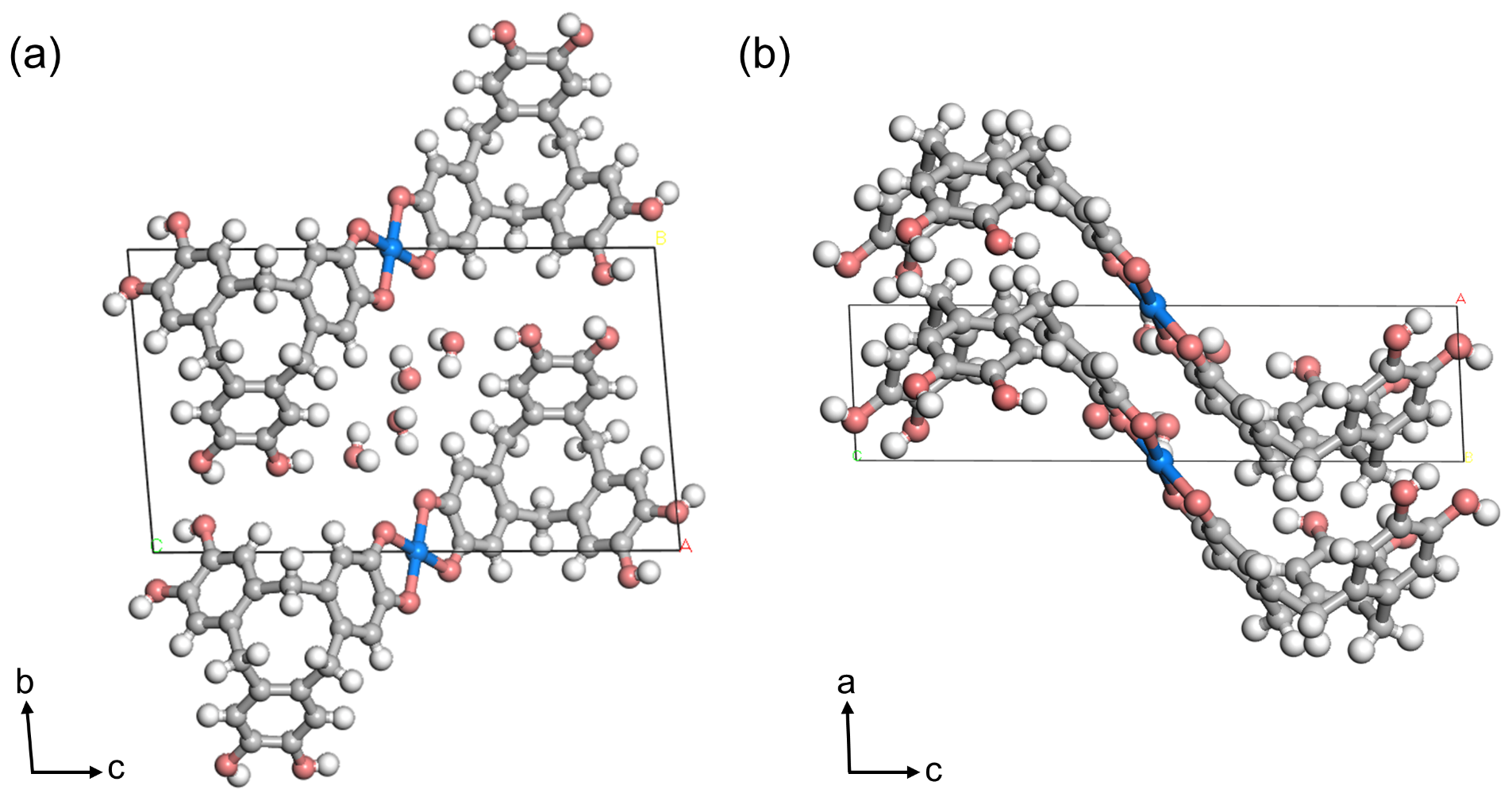


**Fig. S14** Structural model of 2D-Cu-HOF single crystal along the (**a**) *a*-axis and (**b**) *b*-axis direction


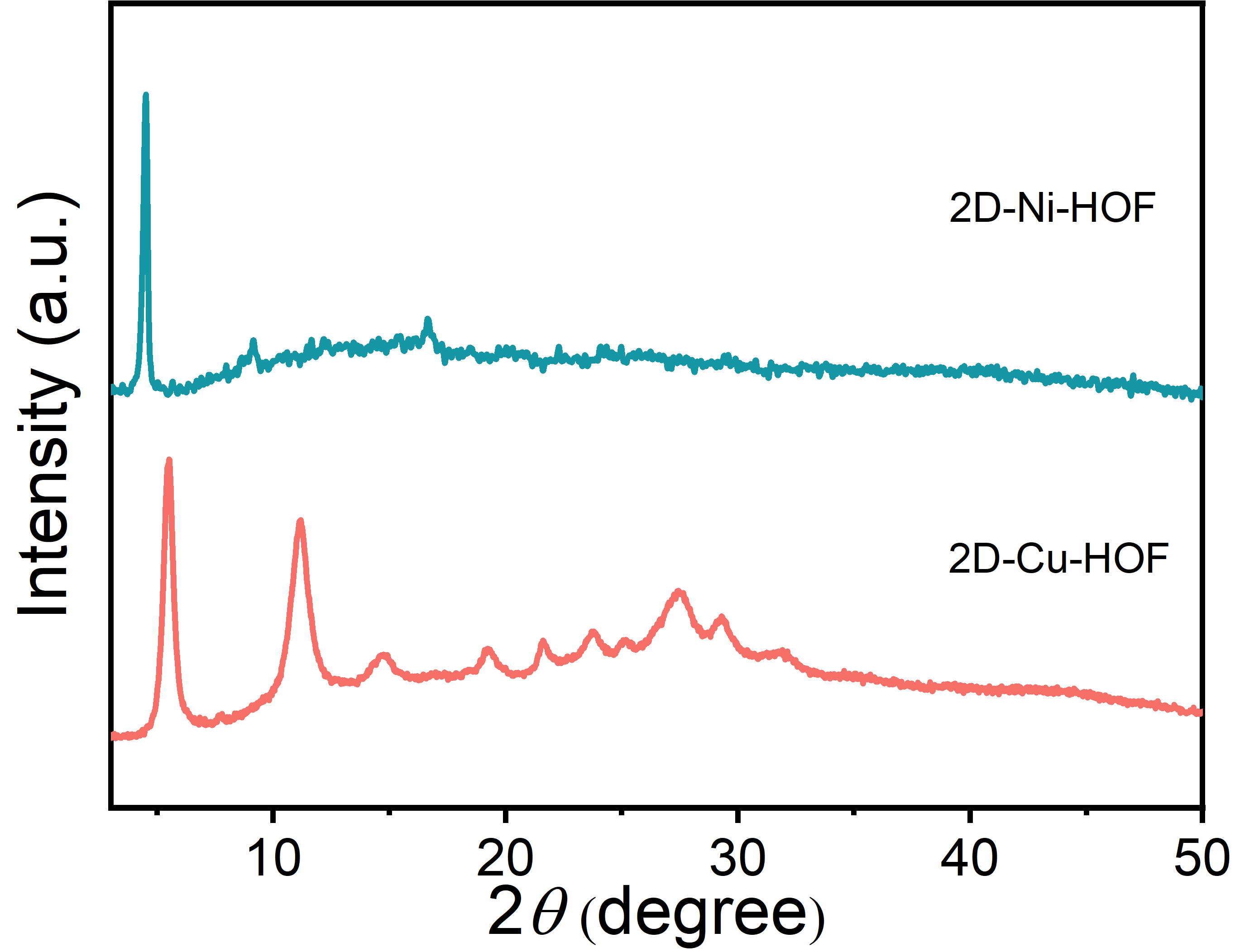


**Fig. S15** PXRD patterns of 2D-Cu-HOF and 2D-Ni-HOF


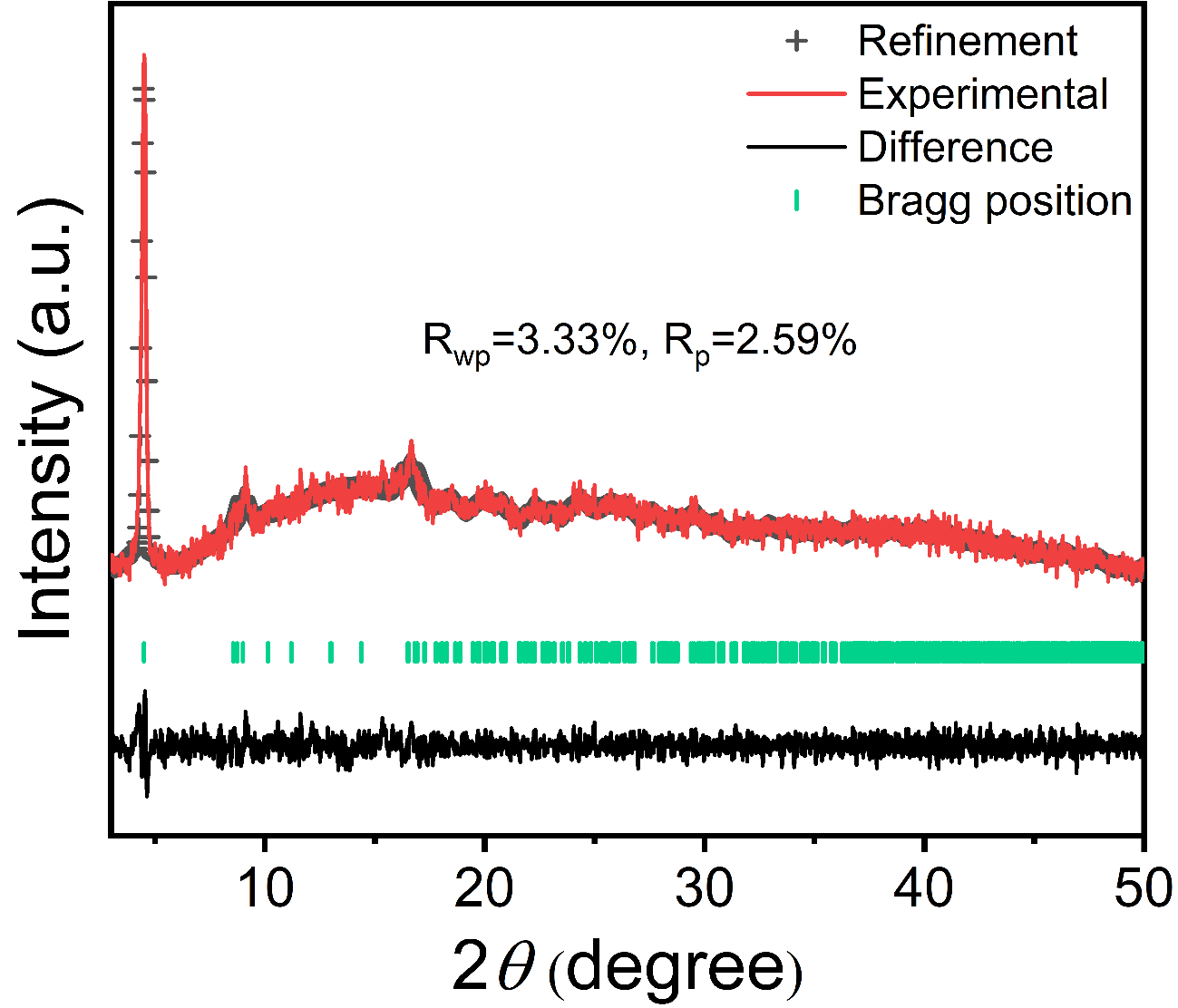


**Fig. S16** Experimental and Pawley refined PXRD patterns of 2D-Ni-HOF


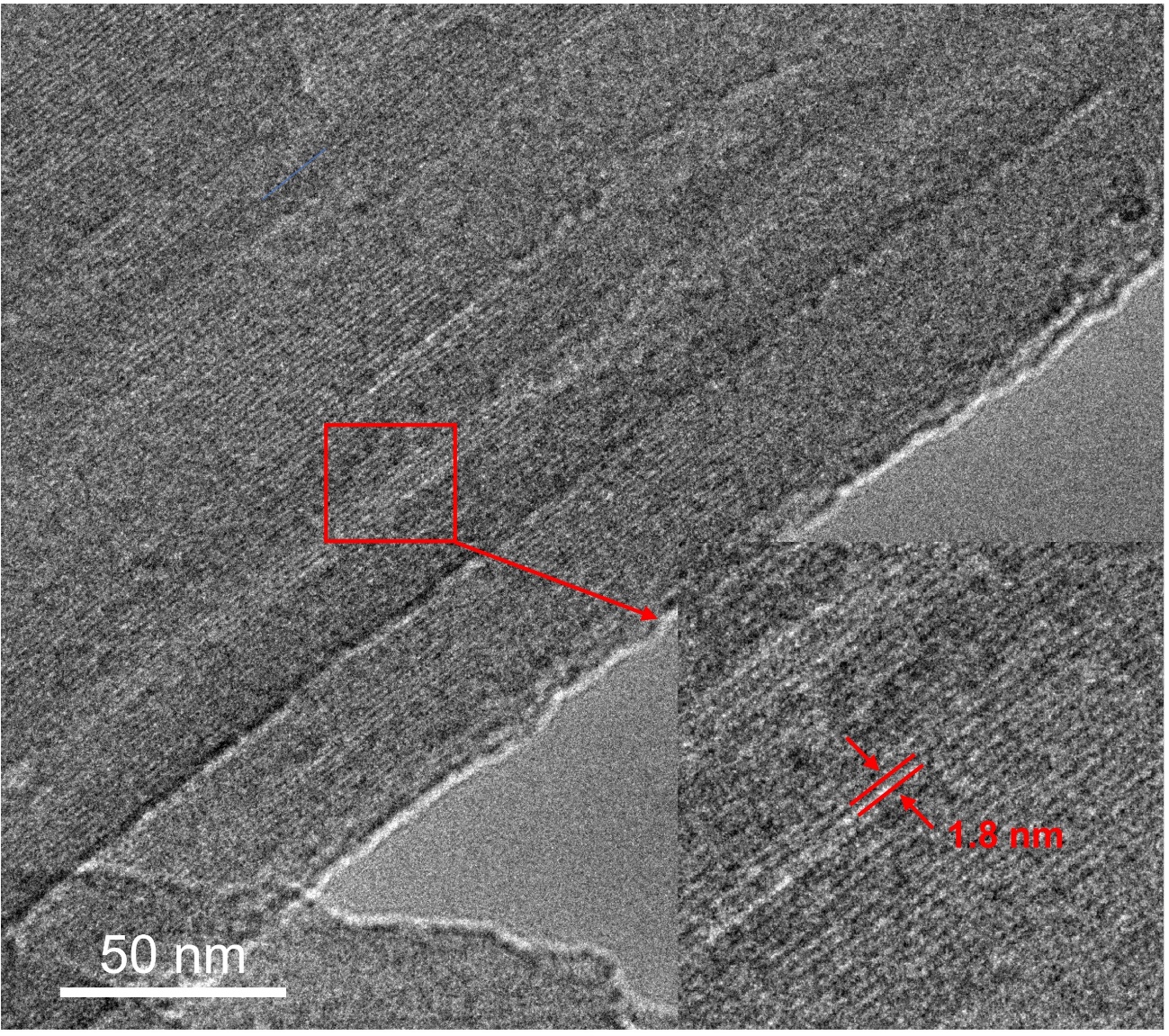


**Fig. S17** HR-TEM image of 2D-Ni-HOF


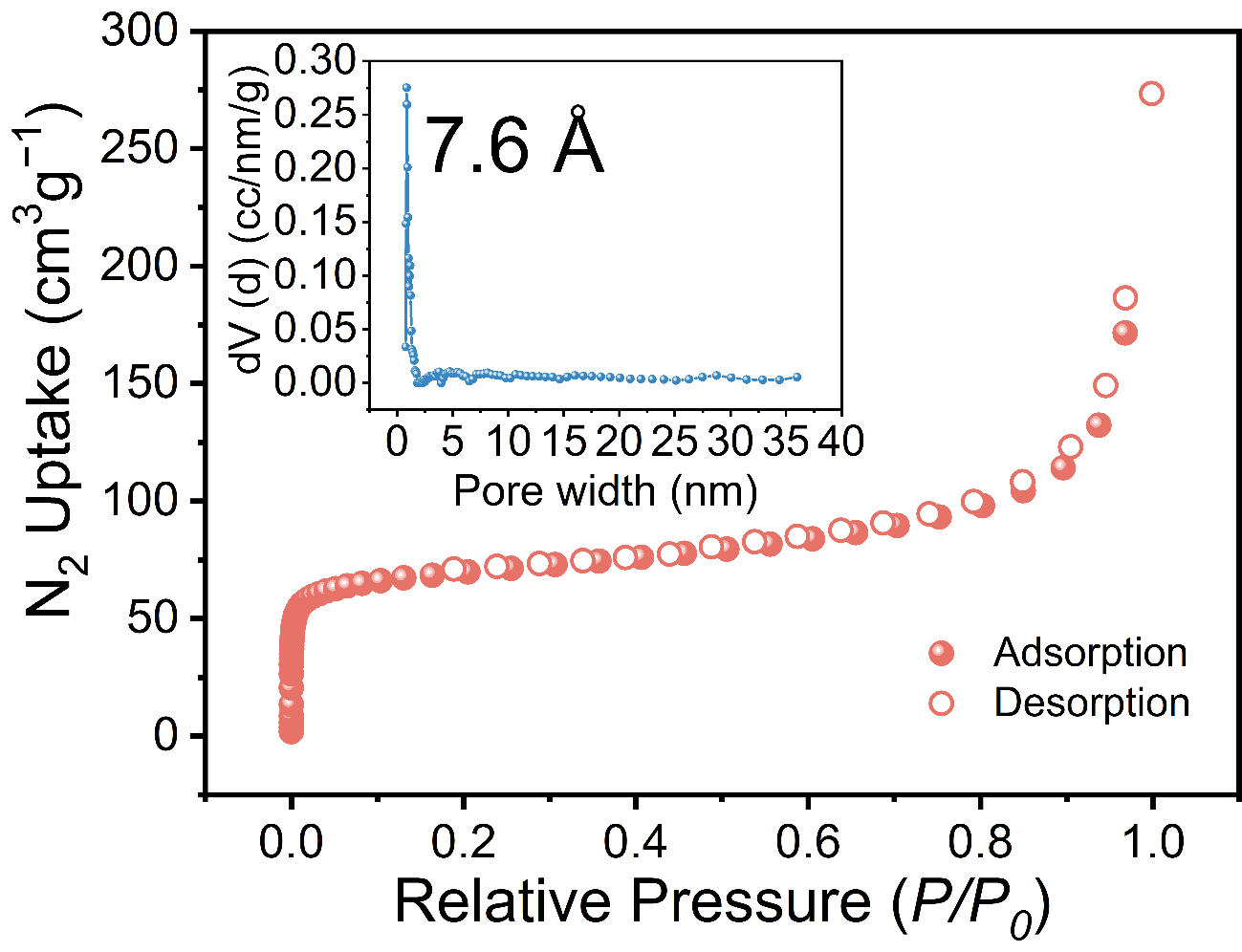


**Fig. S18** N_2_ sorption isotherm of 2D-Cu-HOF at 77 K, insert: the pore width distribution


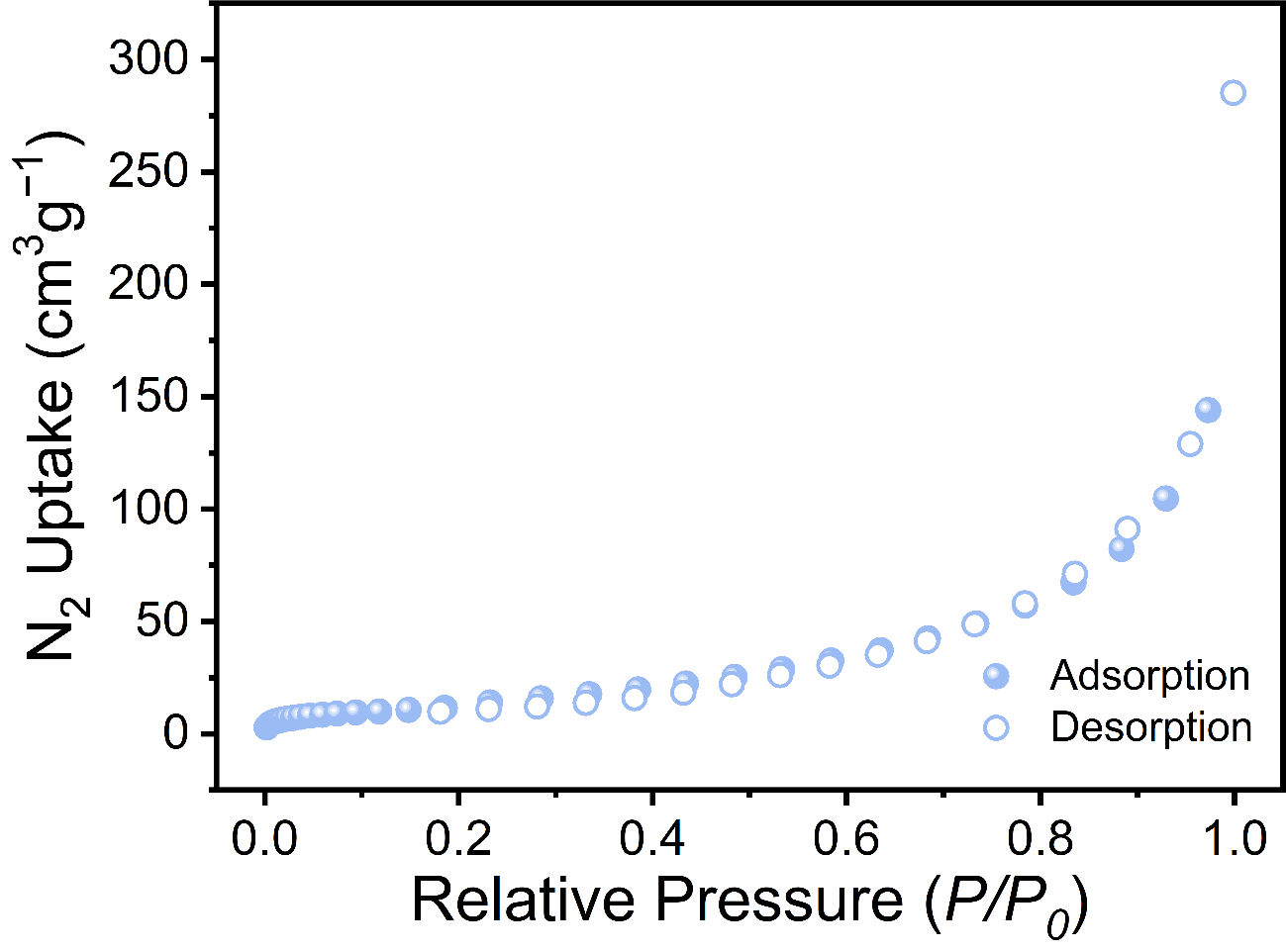


**Fig. S19** N_2_ sorption isotherm of 2D-Ni-HOF at 77 K


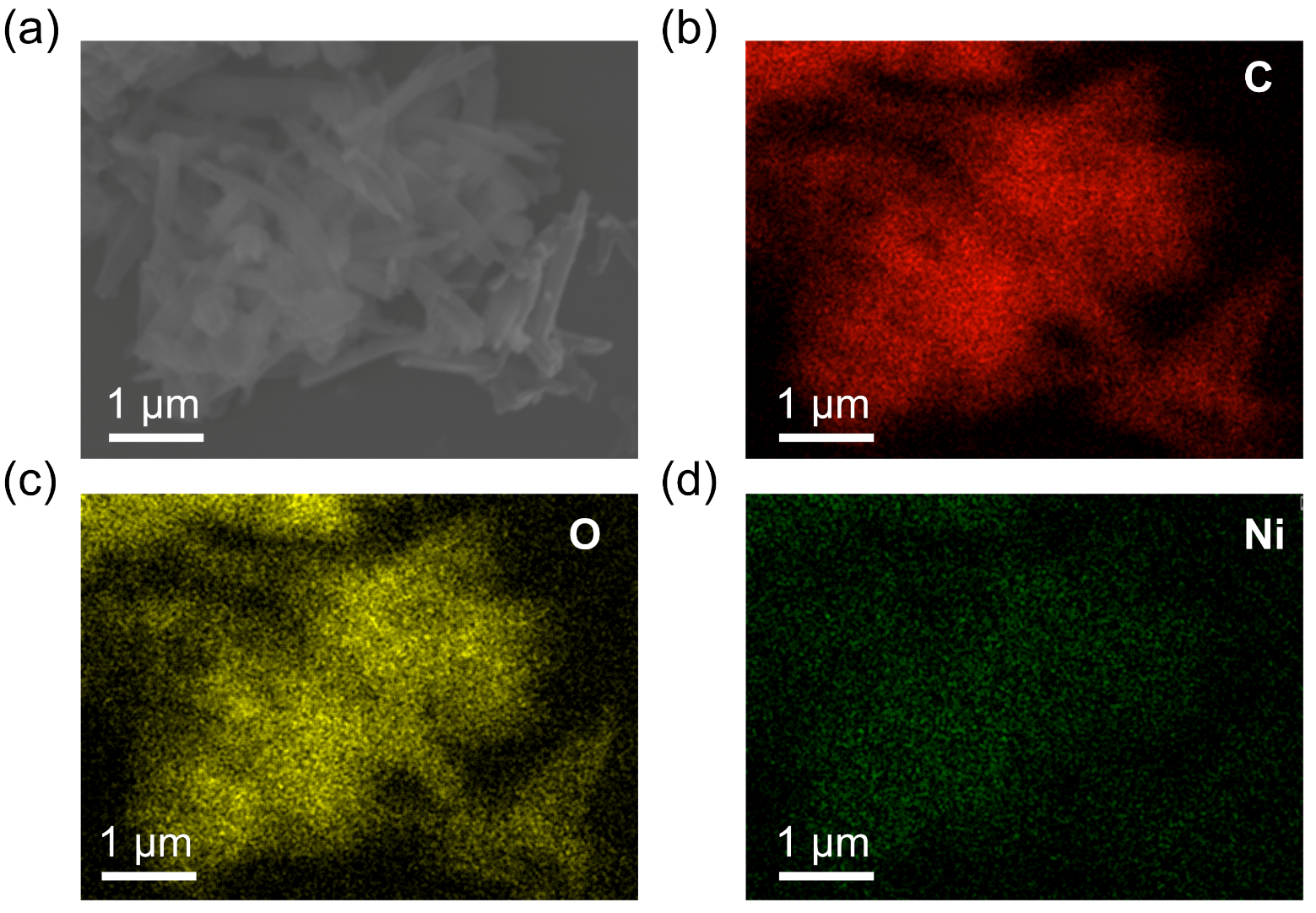


**Fig. S20** EDS elemental mapping of 2D-Ni-HOF


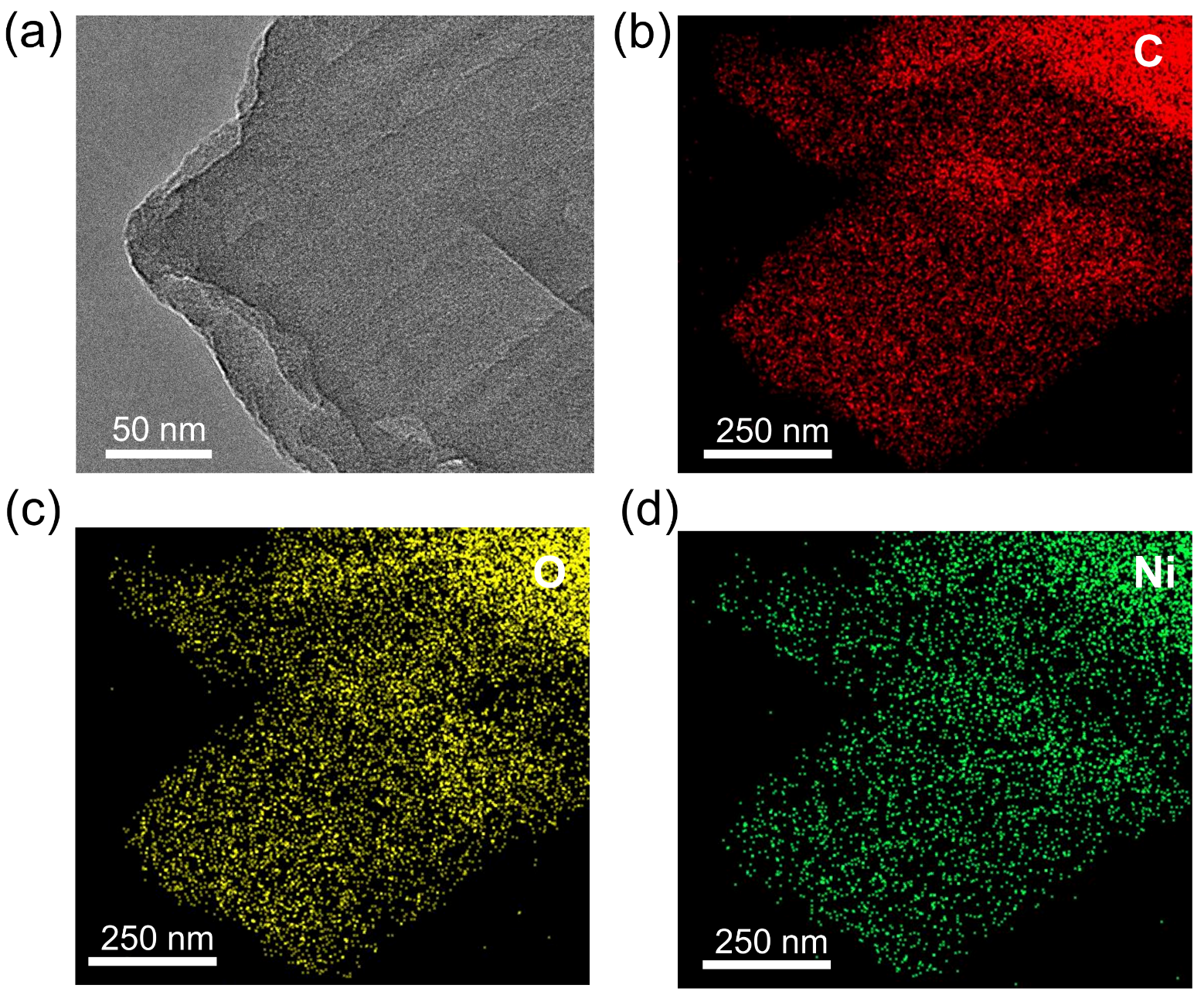


**Fig. S21** TEM elemental mapping of 2D-Ni-HOF


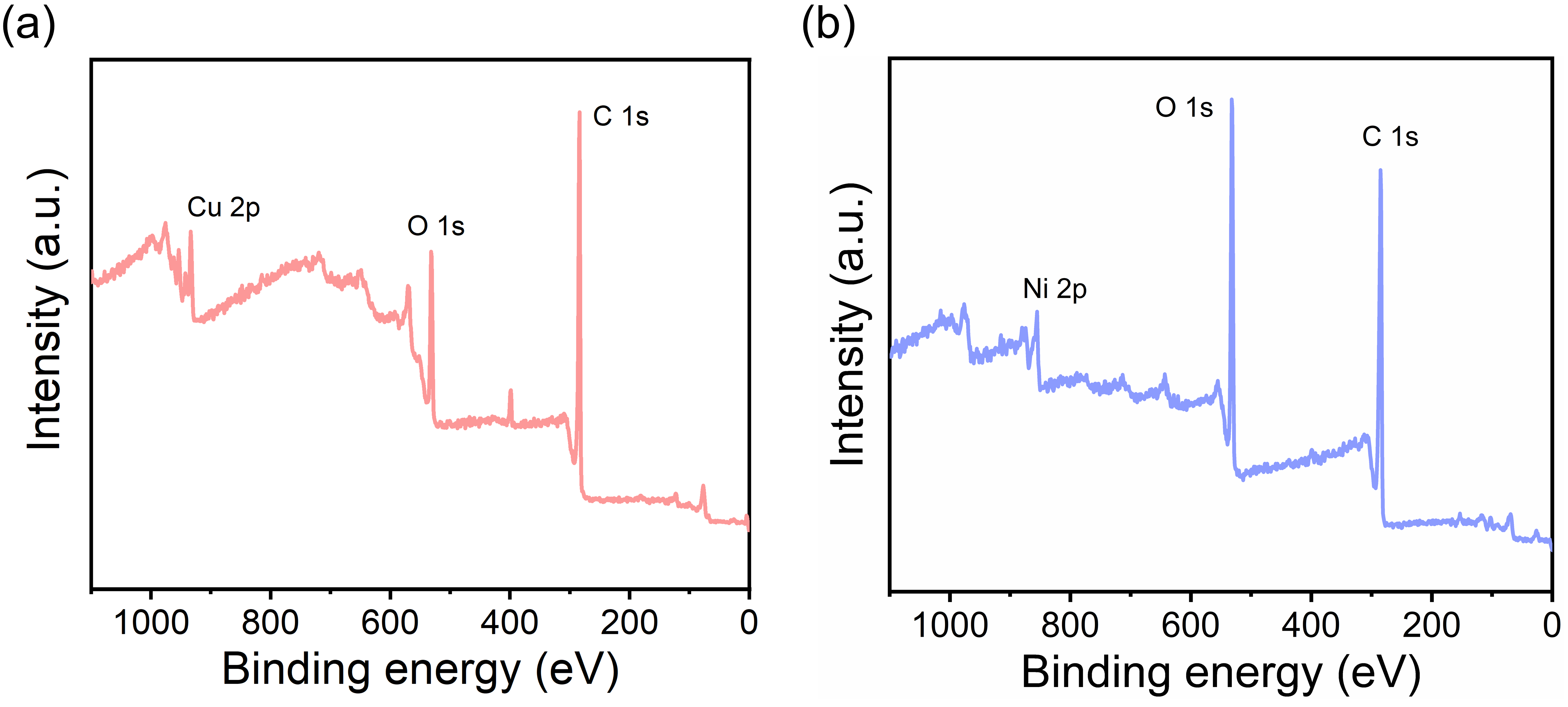


**Fig. S22** XPS survey spectra of (**a**) 2D-Cu-HOF and (**b**) 2D-Ni-HOF


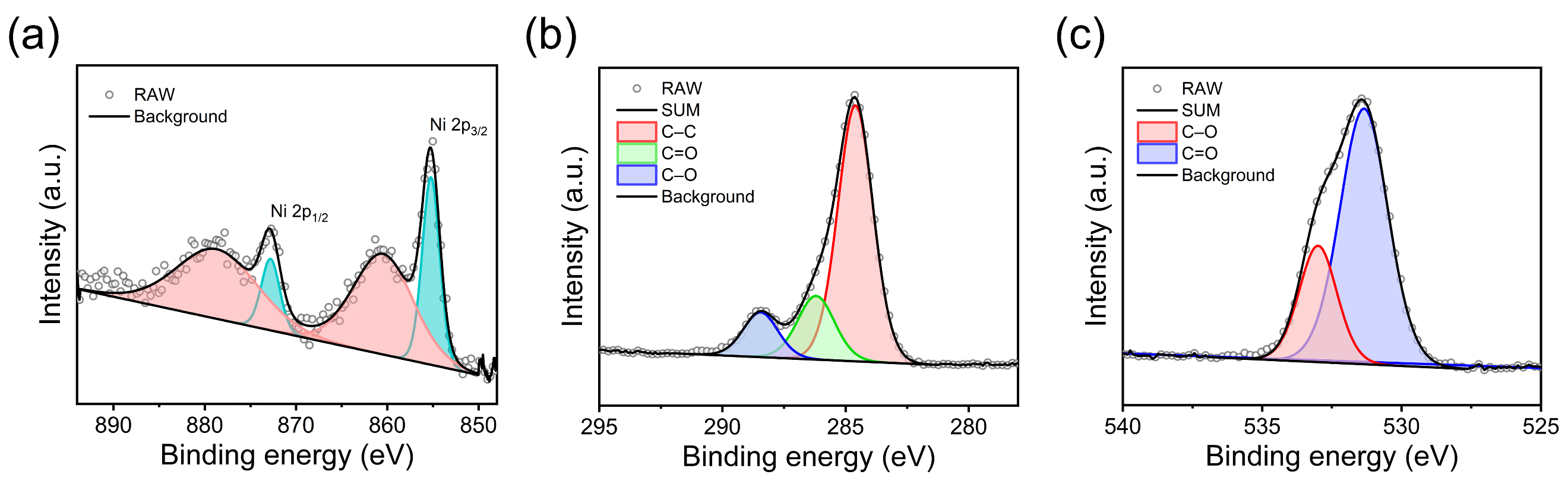


**Fig. S23** High-resolution XPS (**a**) Ni 2p, (**b**) C 1s, and (**c**) O 1s spectra of 2D-Ni-HOF


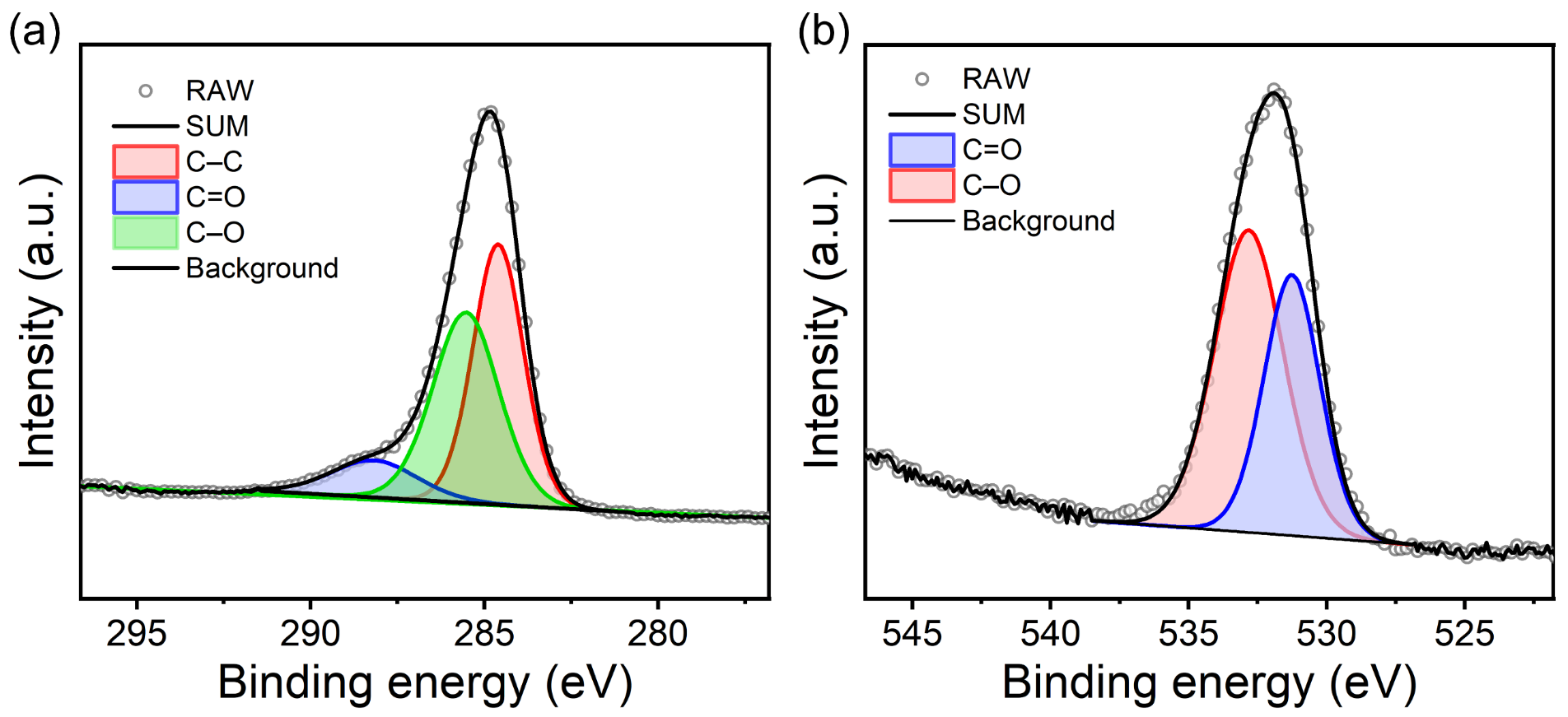


**Fig. S24** High-resolution XPS (**a**) C 1s and (**b**) O 1s spectra of 2D-Cu-HOF


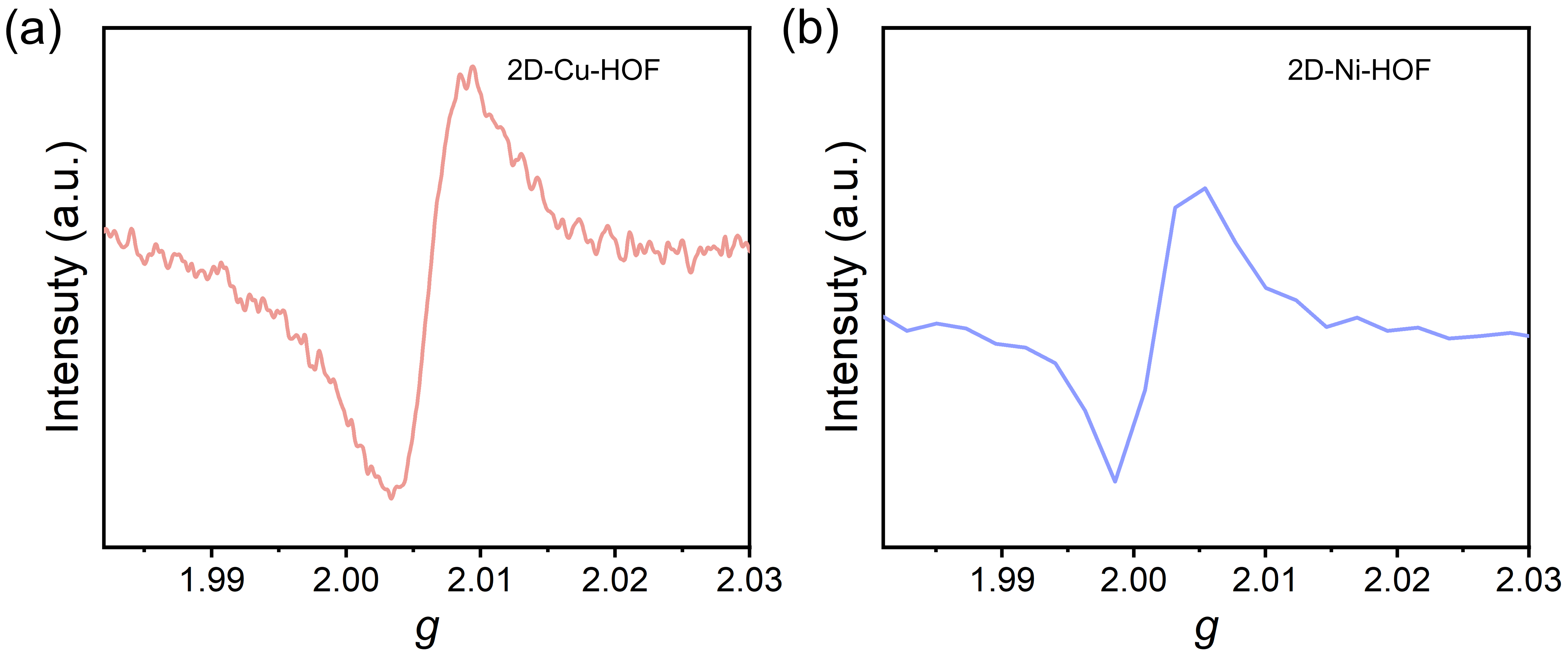


**Fig. S25** EPR spectra of (**a**) 2D-Cu-HOF and (**b**) 2D-Ni-HOF


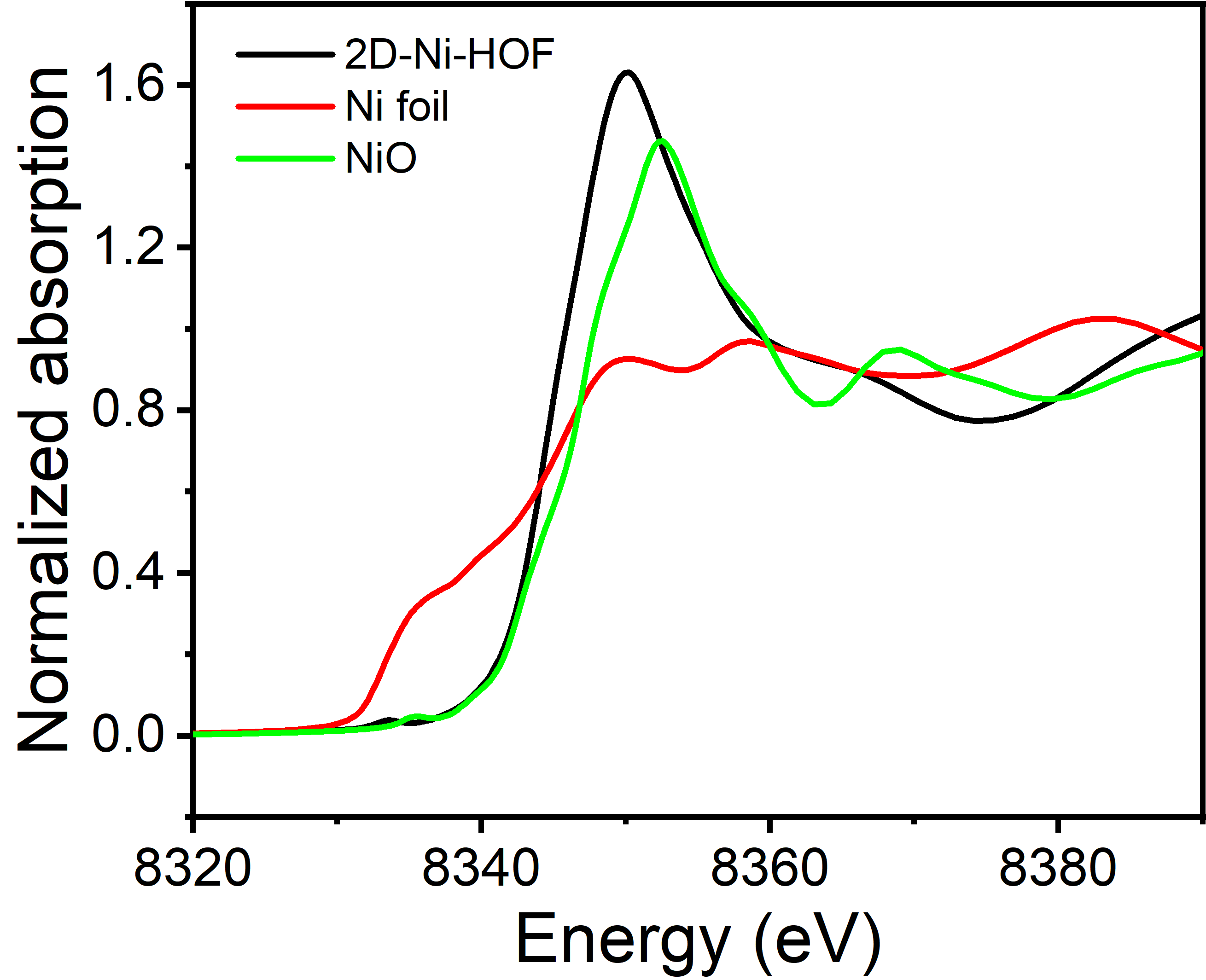


**Fig. S26** Ni *K*-edge XANES spectra of 2D-Ni-HOF


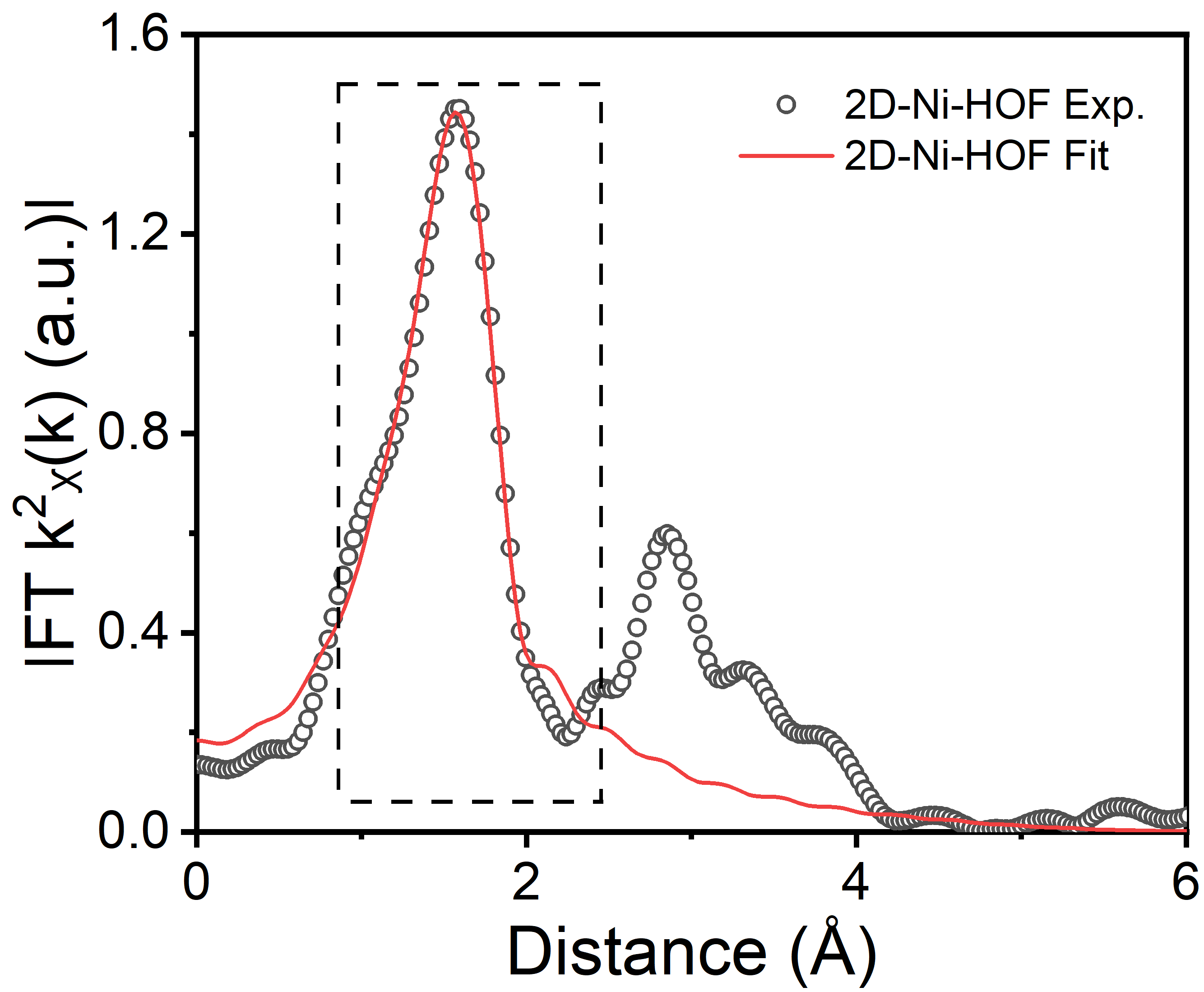


**Fig. S27** Ni *K*-edge EXAFS fitting of 2D-Ni-HOF


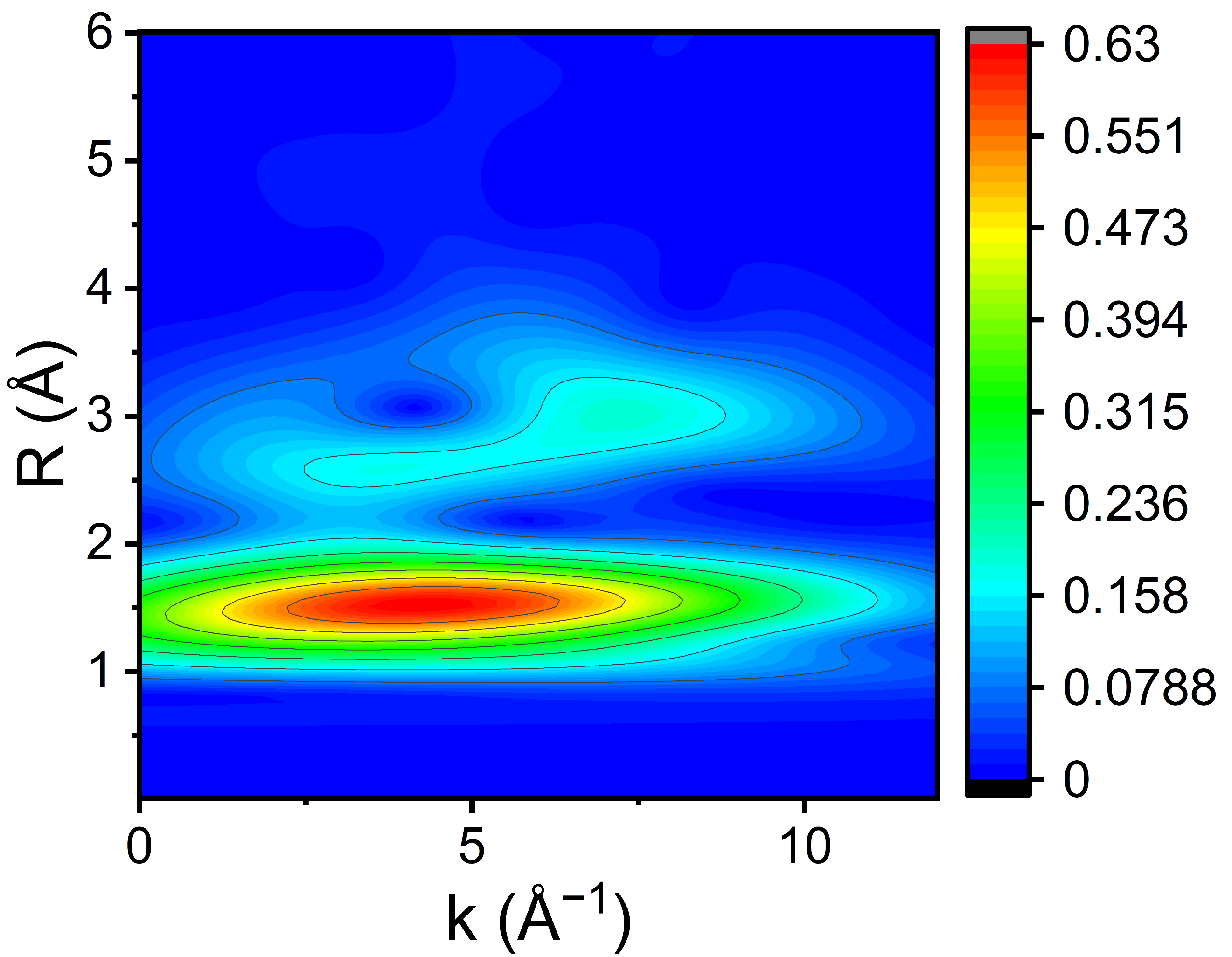


**Fig. S28** Ni *K*-edge wavelet transform of 2D-Ni-HOF


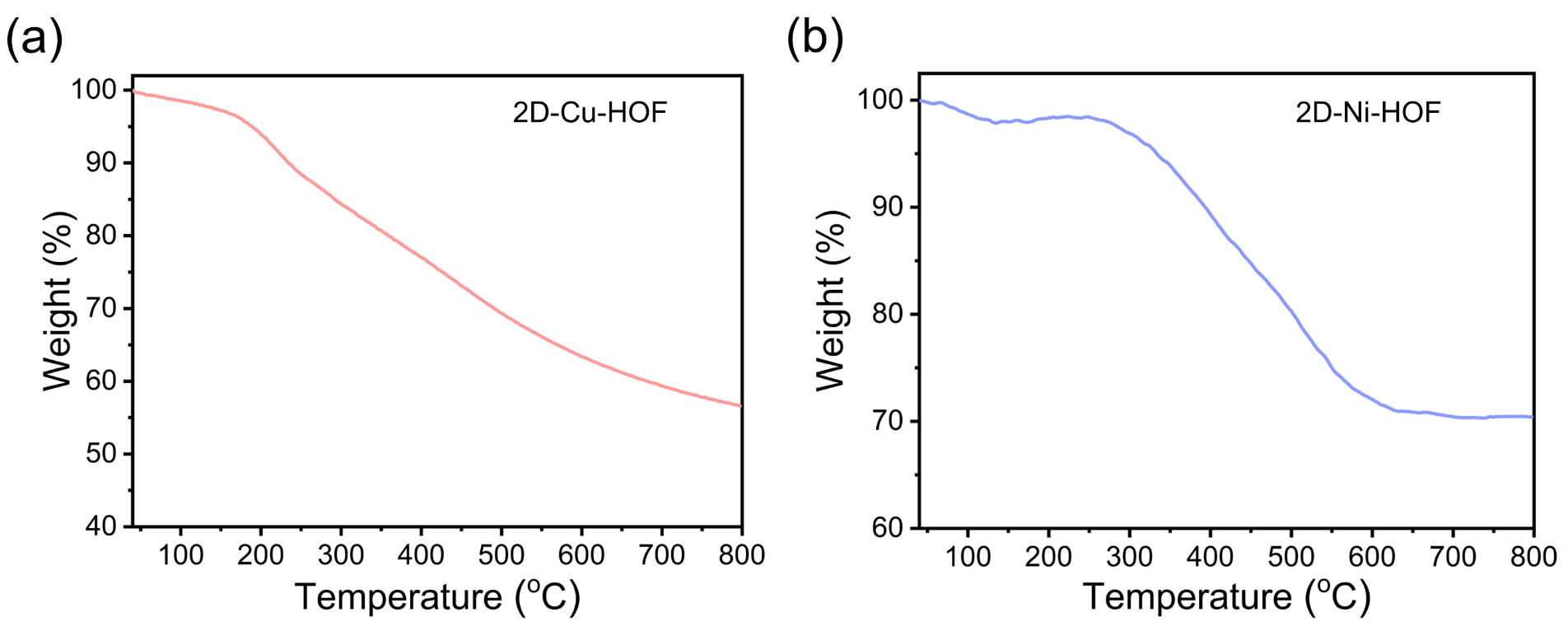


**Fig. S29** TGA curves of (**a**) 2D-Cu-HOF and (**b**) 2D-Ni-HOF under N_2_ atmosphere


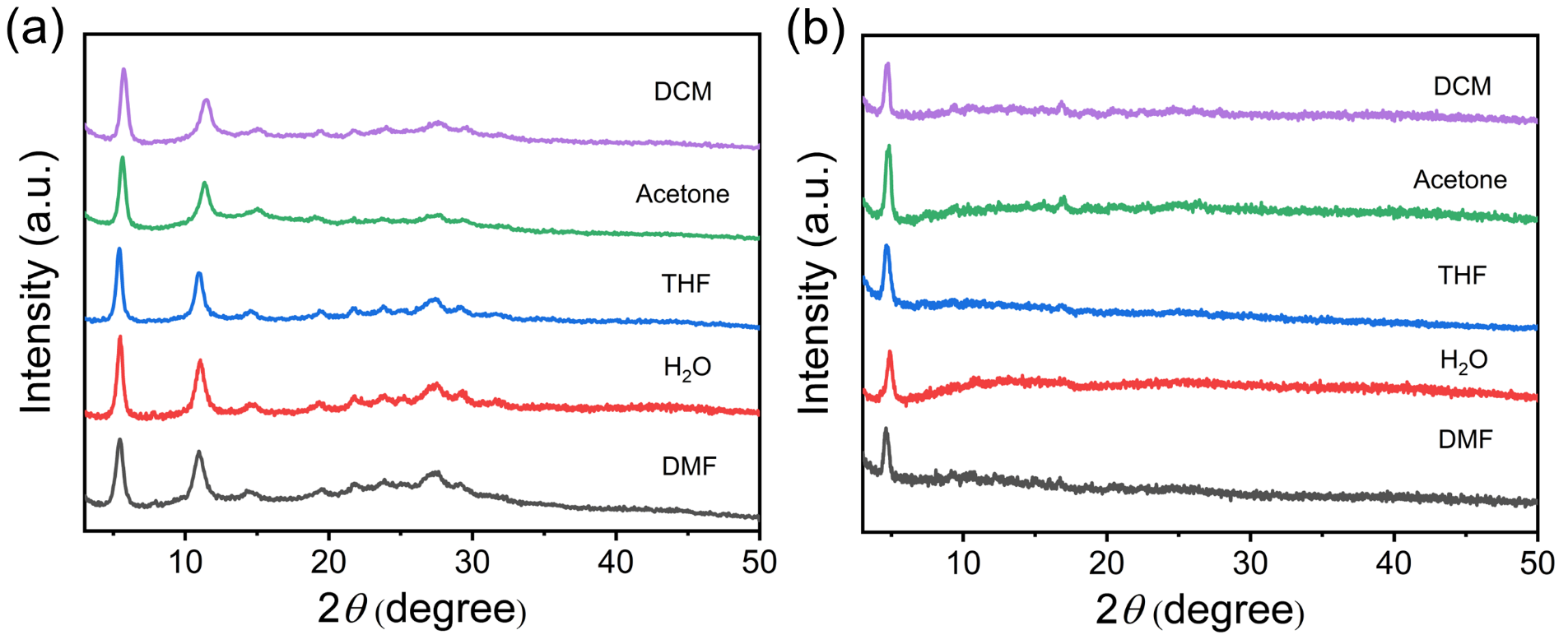


**Fig. S30** Comparison of PXRD patterns of (**a**) 2D-Cu-HOF and (**b**) 2D-Ni-HOF after immersing in different solvents for three days


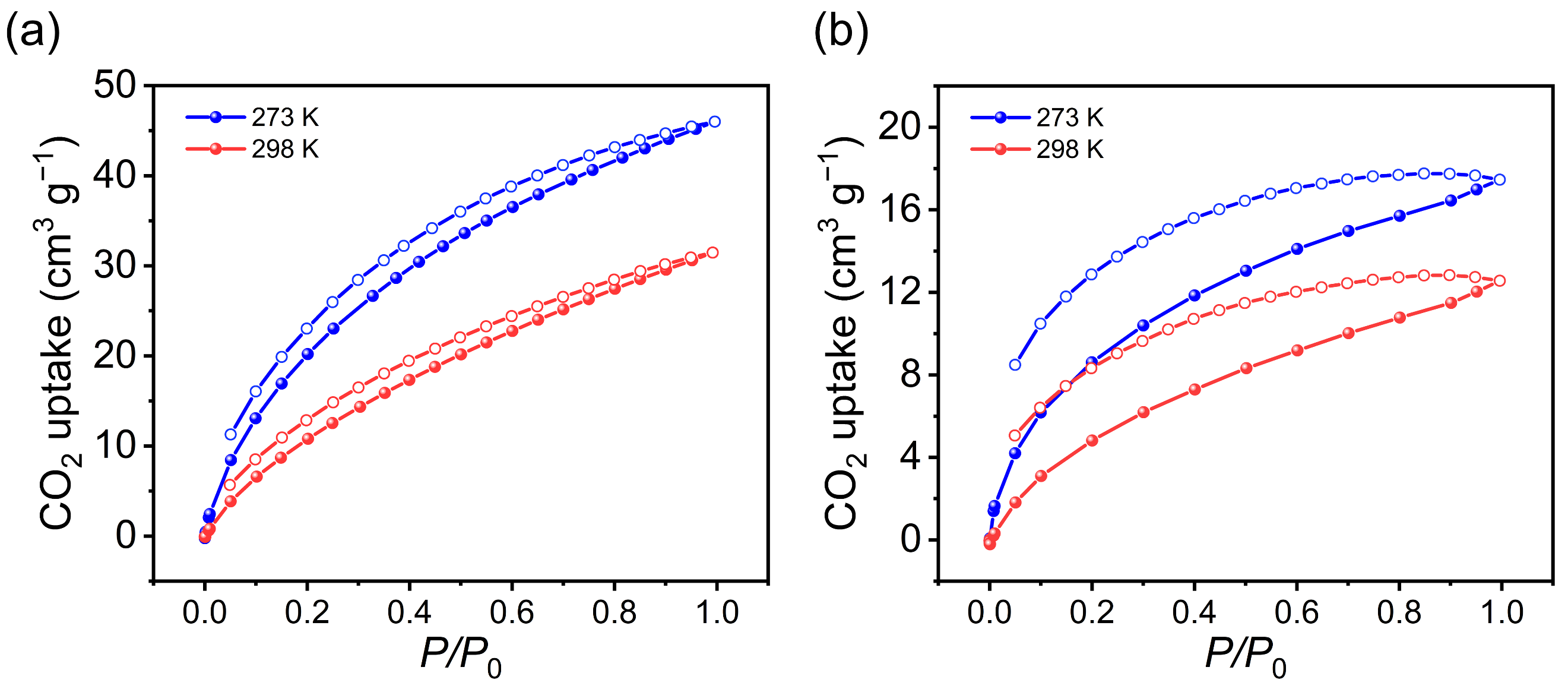


**Fig. S31** CO_2_ adsorption isotherms of (**a**) 2D-Cu-HOF and (**b**) 2D-Ni-HOF at 273 K (blue) and 298 K (red)


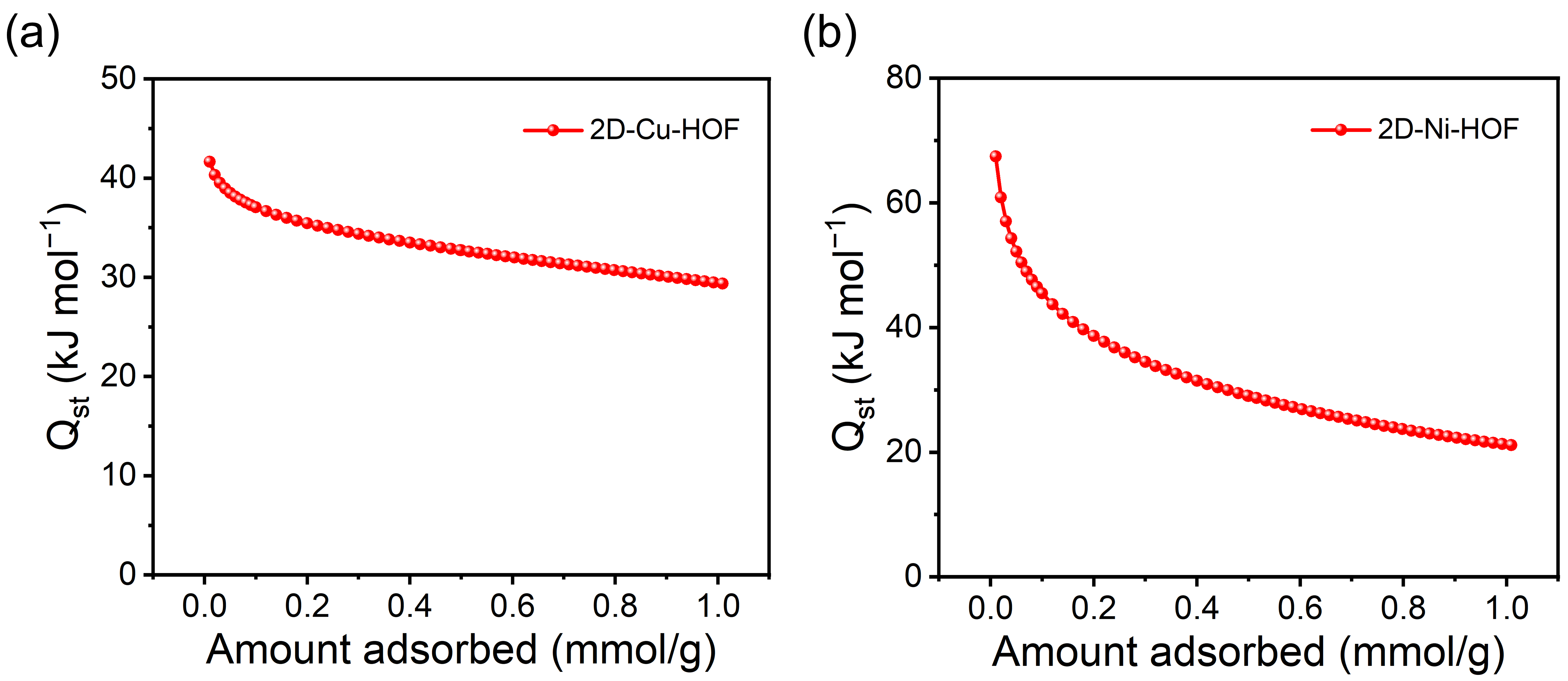


**Fig. S32** Q_st_ of (**a**) 2D-Cu-HOF and (**b**) 2D-Ni-HOF calculated from the CO_2_ adsorption isotherms by the Clausius–Clapeyron approach


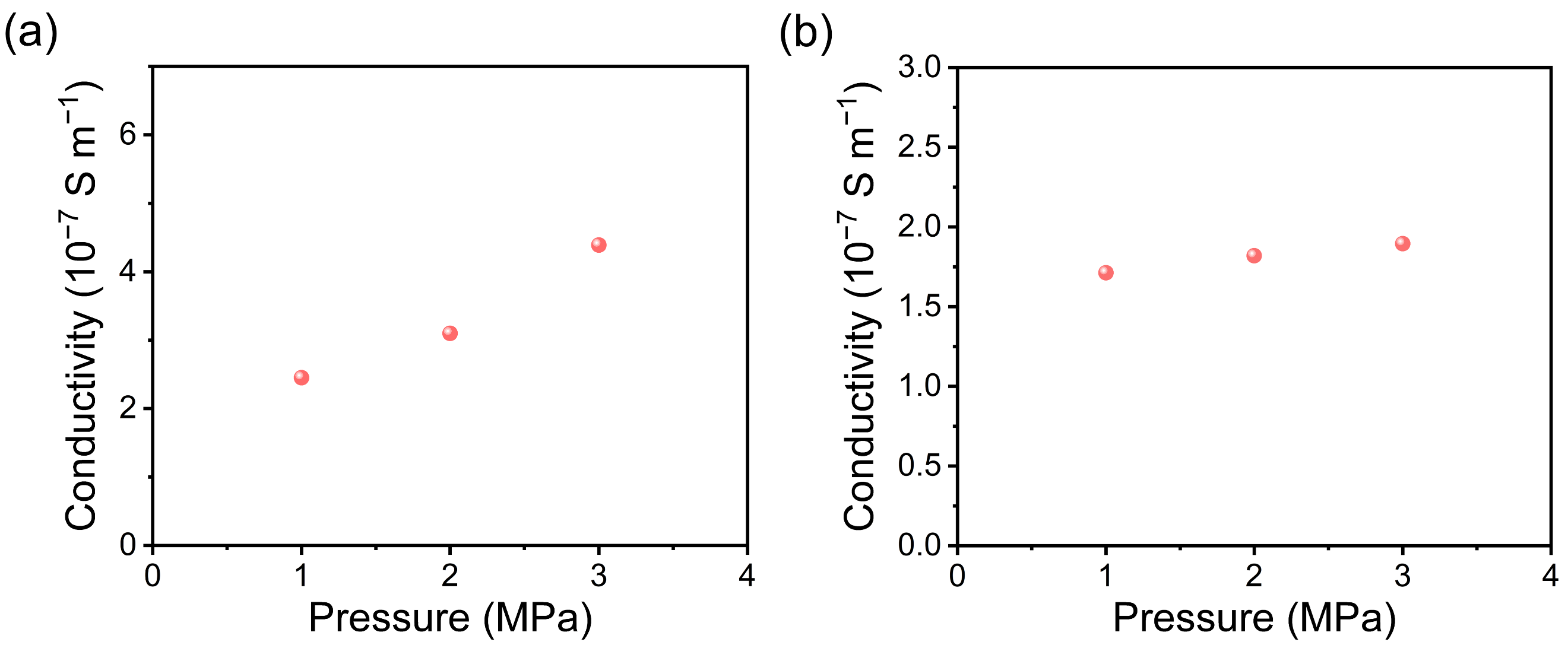


**Fig. S33** The conductivity of (**a**) 2D-Cu-HOF pellet and (**b**) 2D-Ni-HOF pellet under different pressed pressure at room temperature


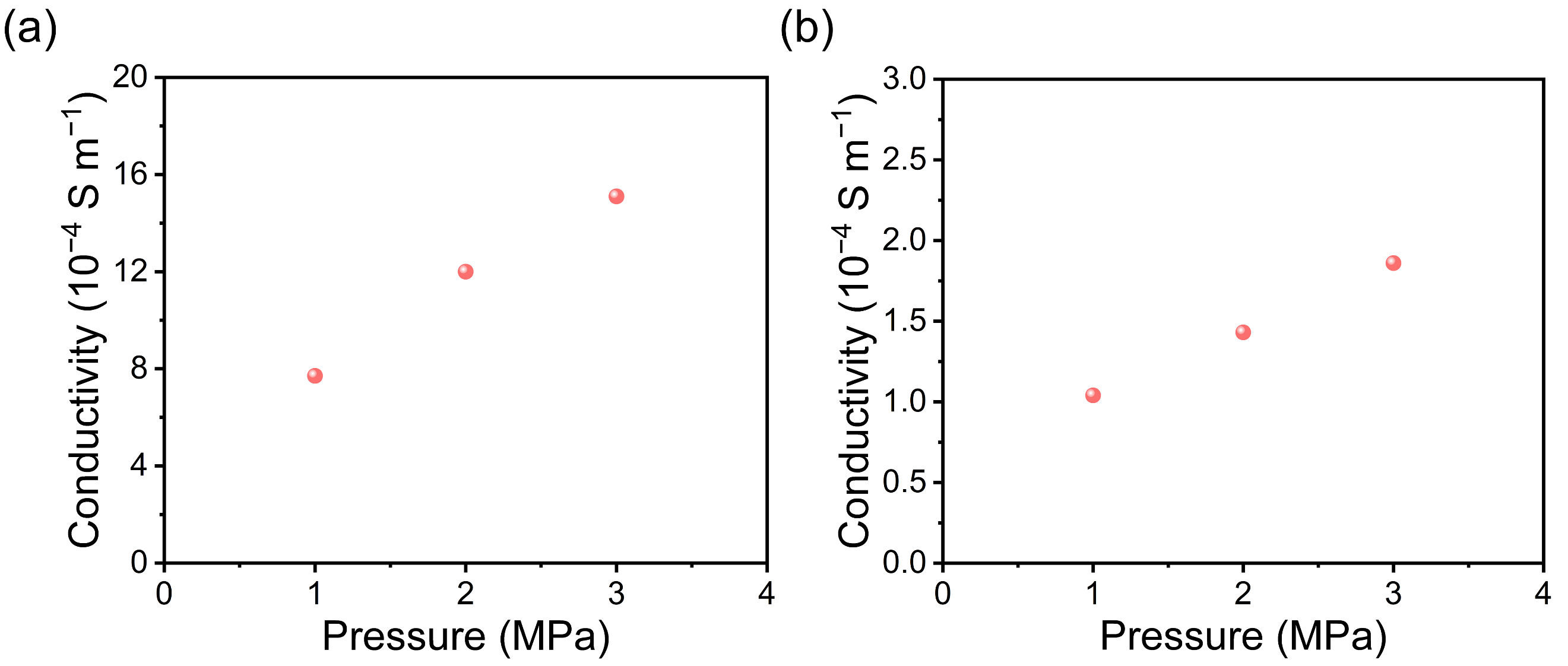


**Fig. S34** The conductivity of iodine-doped (**a**) 2D-Cu-HOF pellet and (**b**) 2D-Ni-HOF pellet under different pressed pressure at room temperature


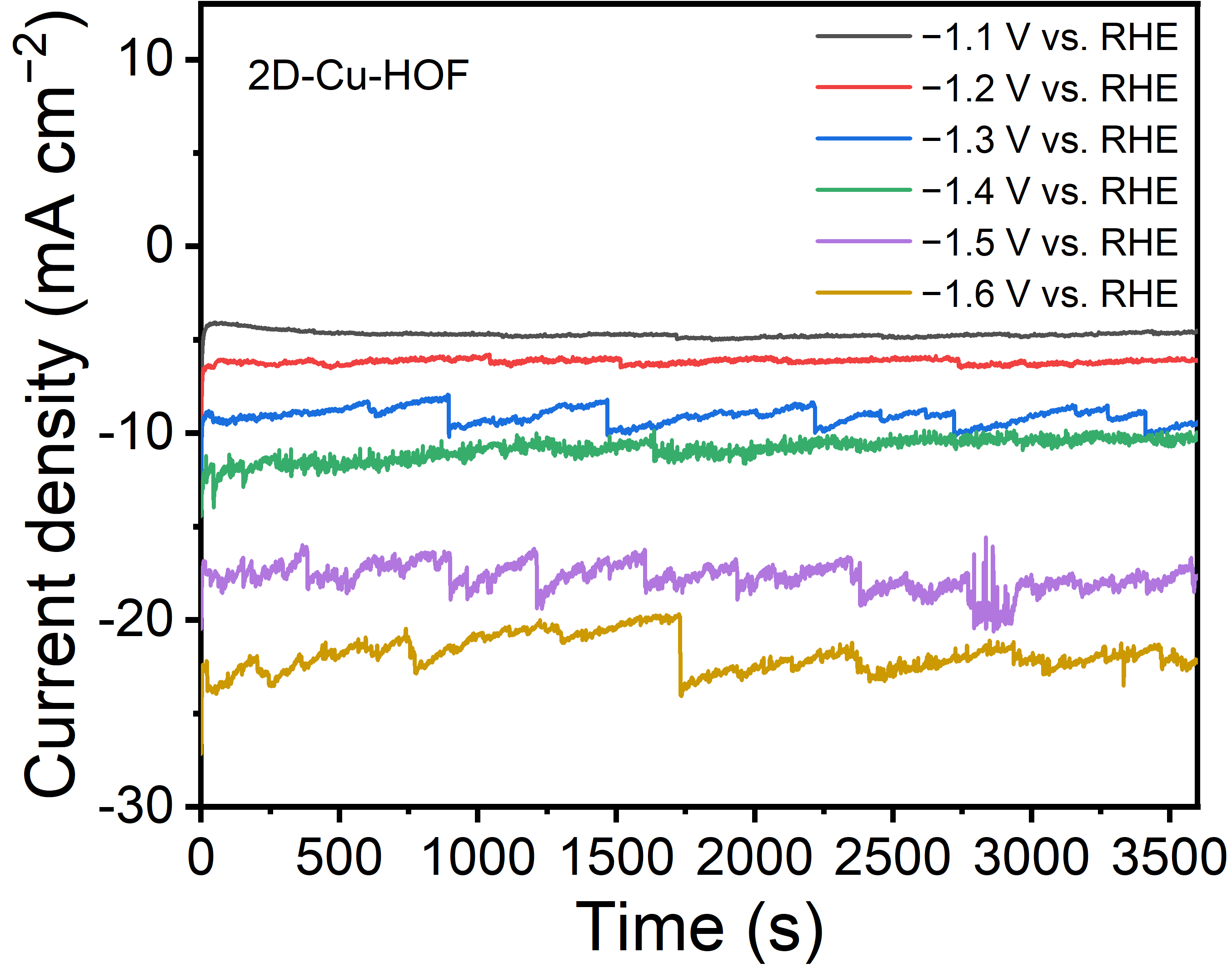


**Fig. S35** Chronoamperometry of the 2D-Cu-HOF


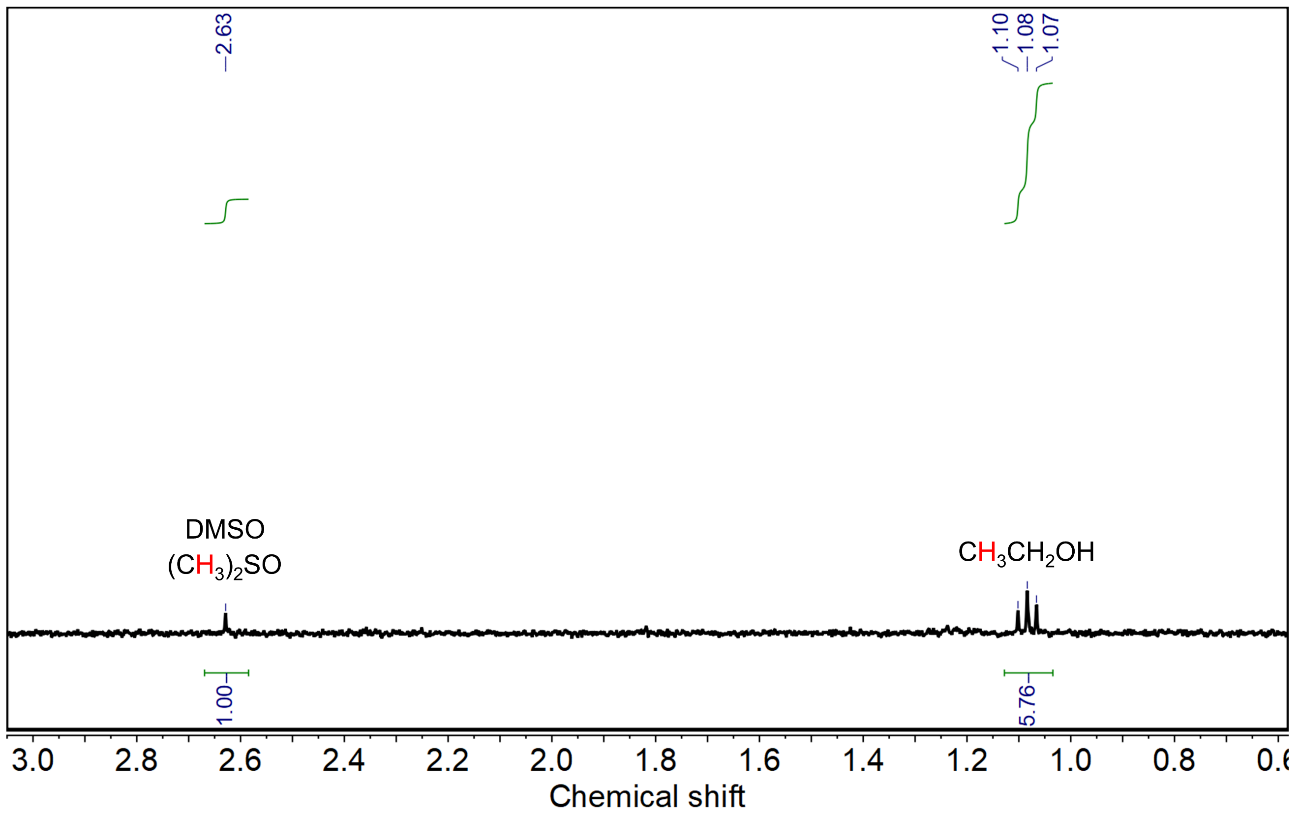


**Fig. S36** ^1^H NMR spectrum of the liquid phase products as catalyzed by 2D-Cu-HOF, wherein DMSO was used as an internal standard under H_2_O signal suppression


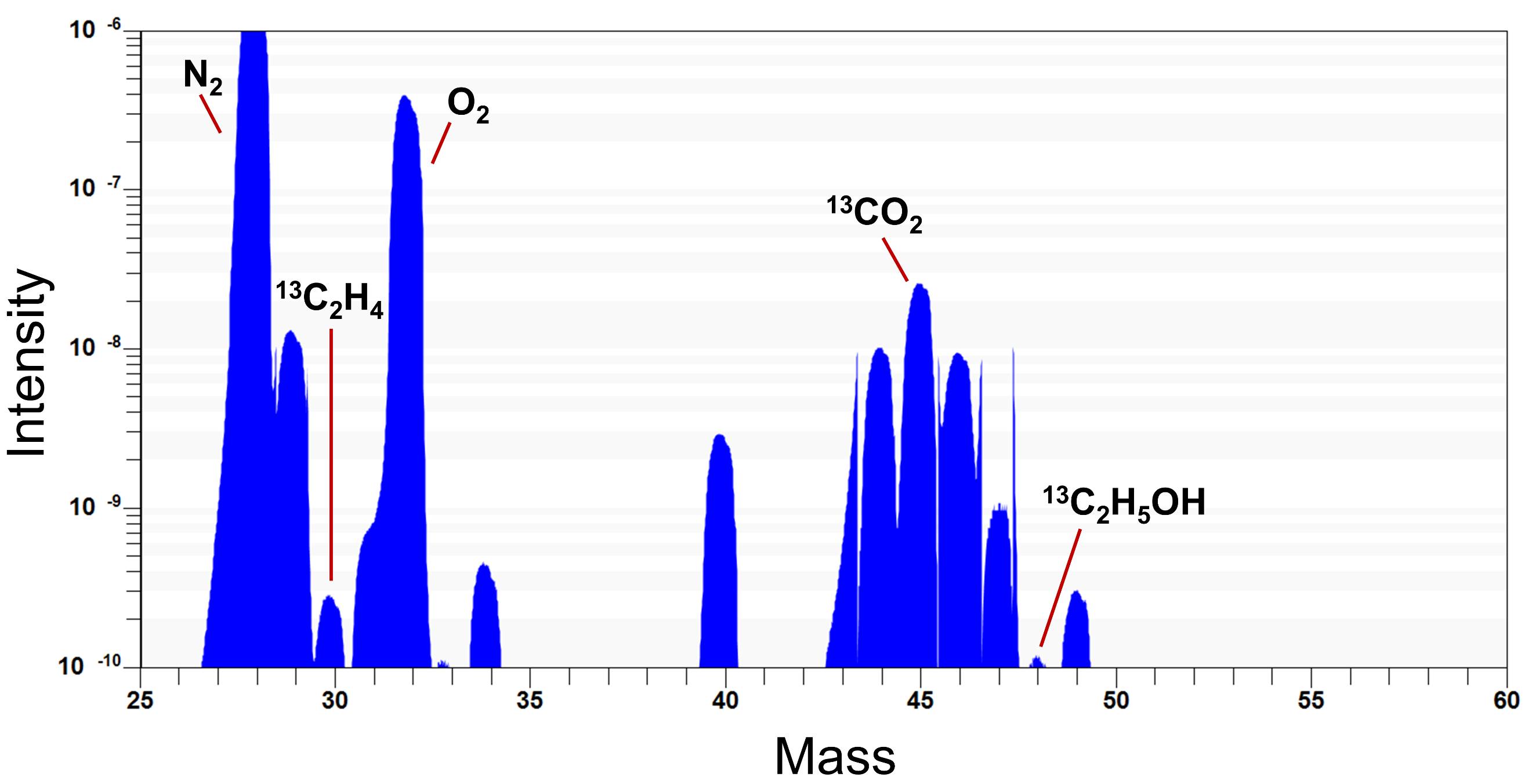


**Fig. S37** Mass spectrum of the CO_2_RR electrolysis products with 2D-Cu-HOF by using ^13^C labeling CO_2_


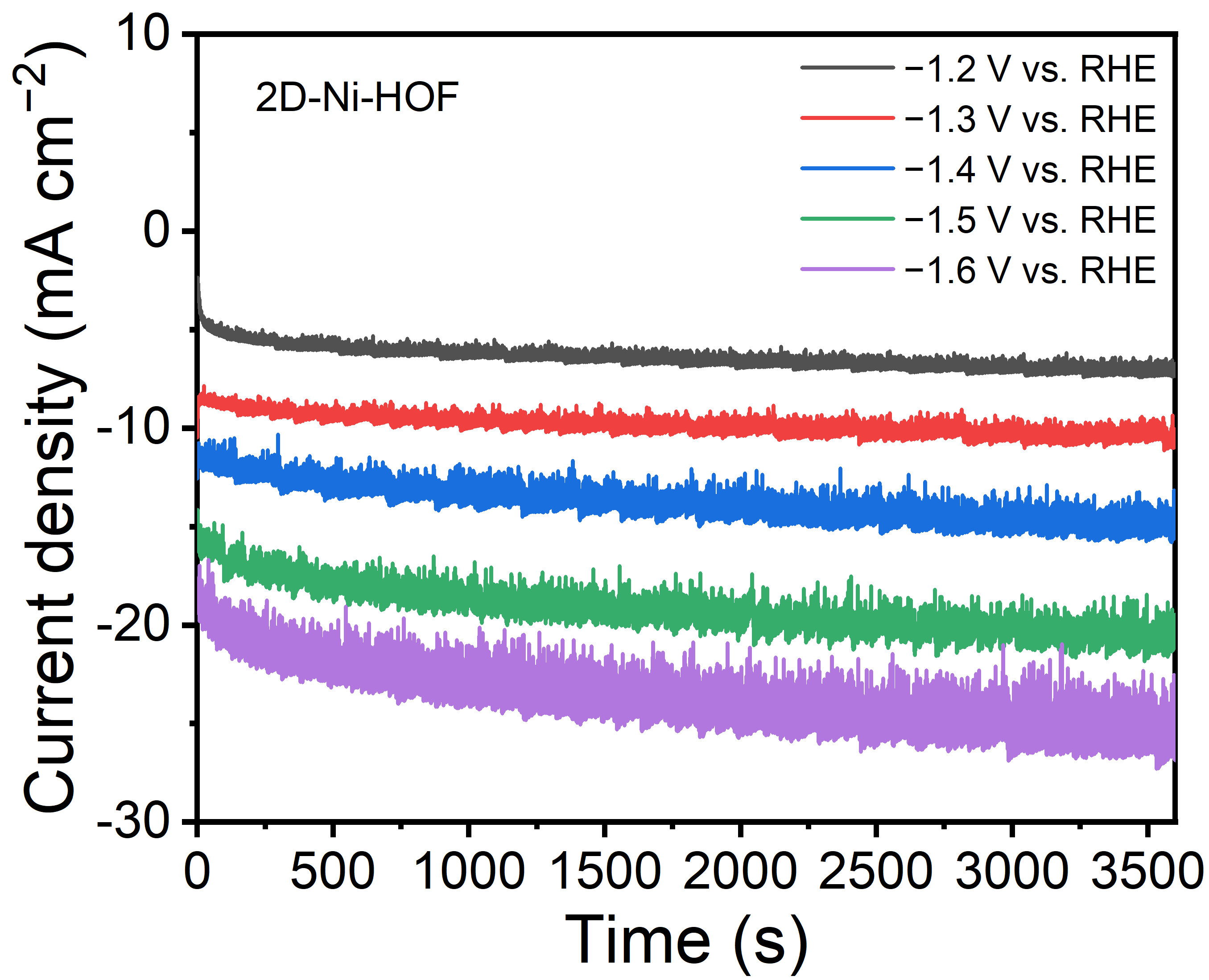


**Fig. S38** Chronoamperometry of the 2D-Ni-HOF


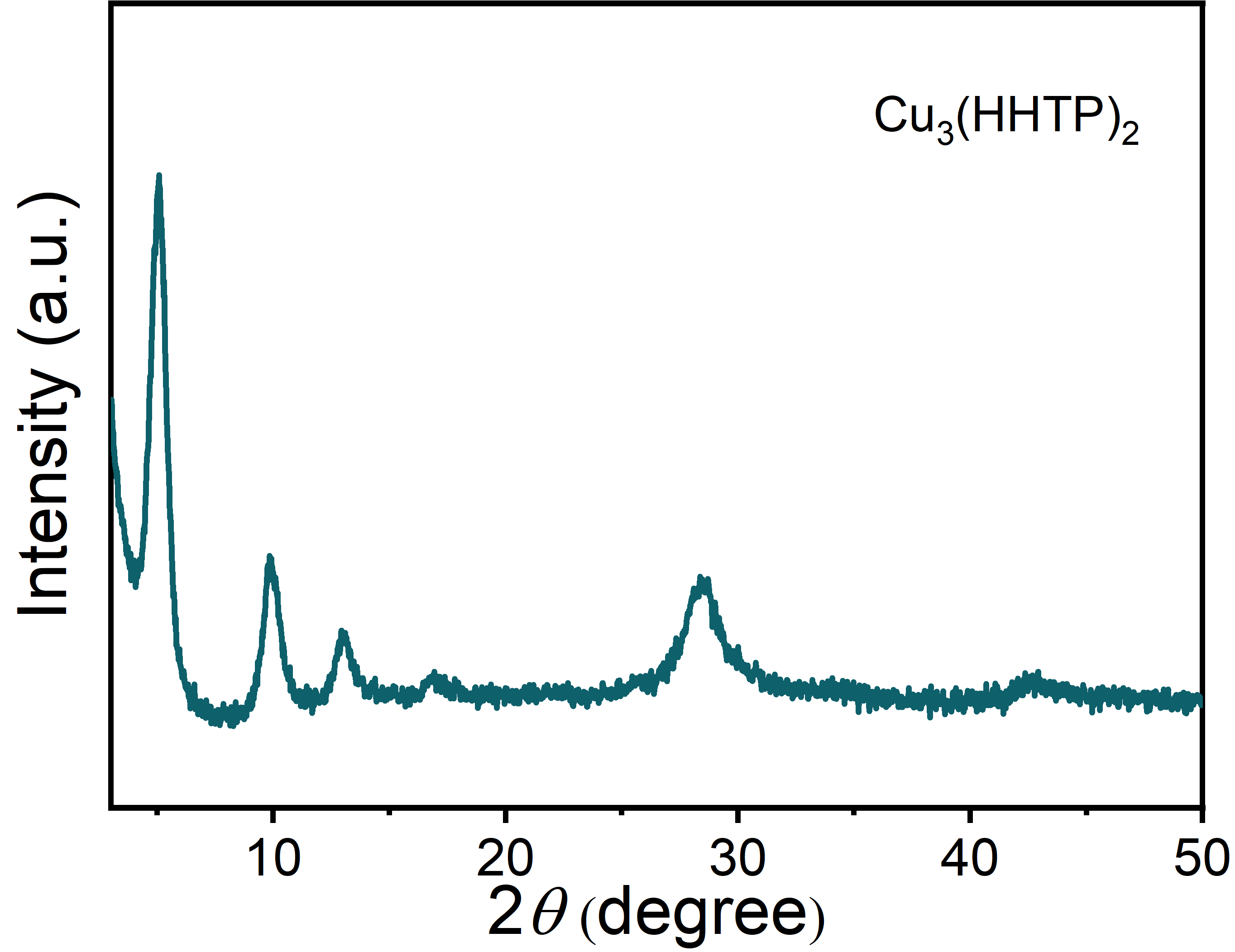


**Fig. S39** PXRD pattern of Cu_3_(HHTP)_2_


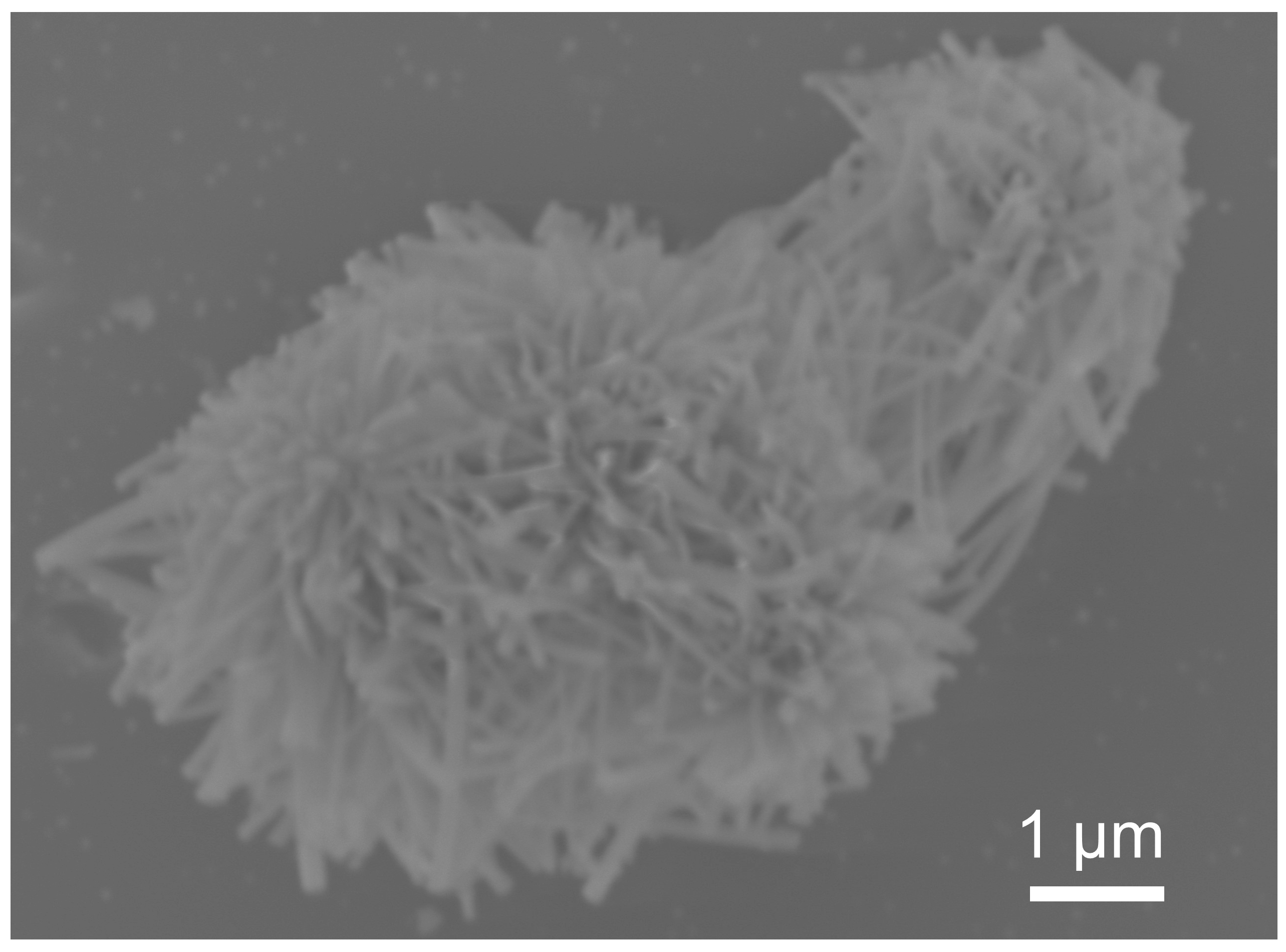


**Fig. S40** SEM image of Cu_3_(HHTP)_2_


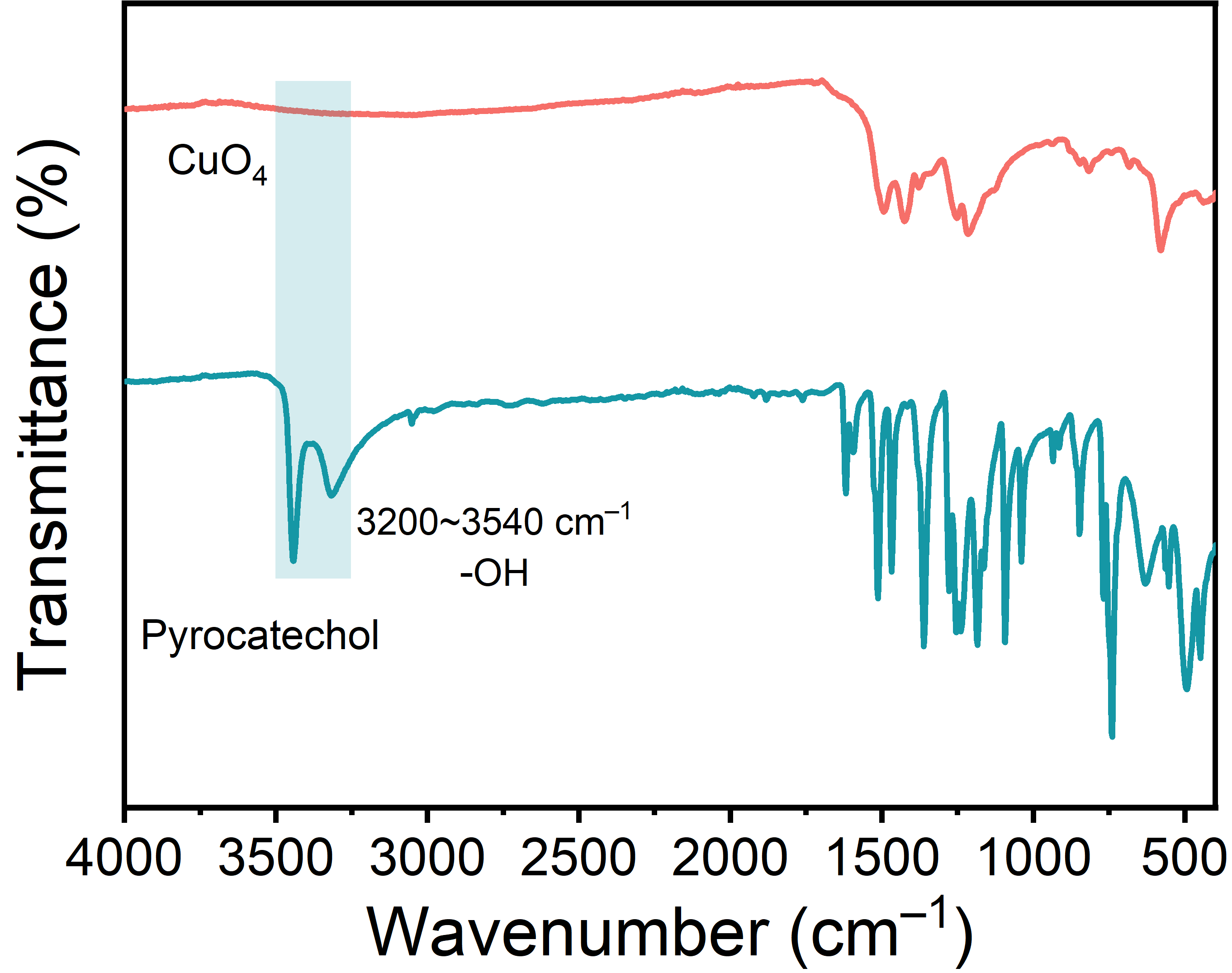


**Fig. S41** FT-IR spectra of pyrocatechol and copper catecholate (CuO_4_)


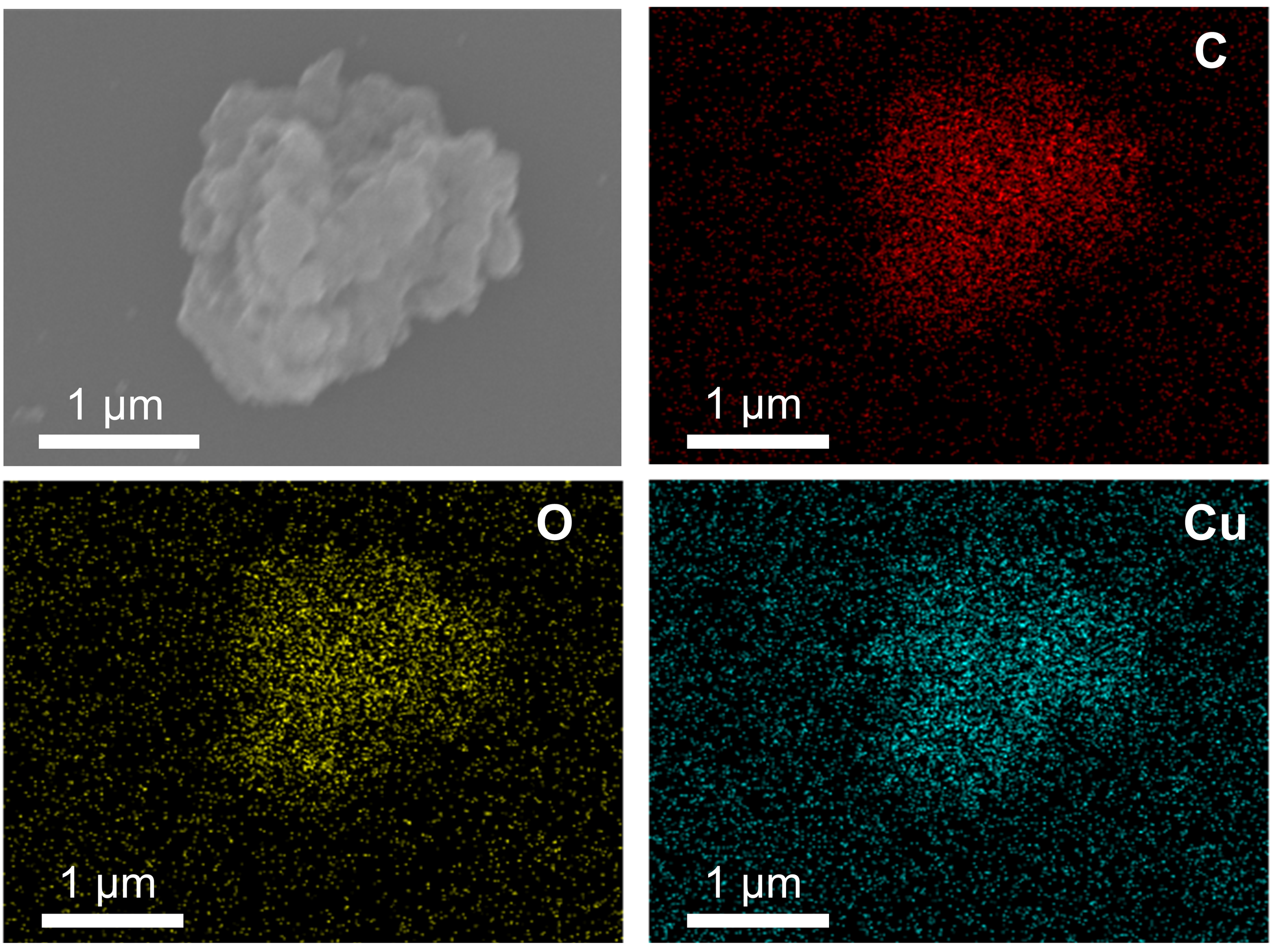


**Fig. S42** EDS elemental mapping of CuO_4_@PPy


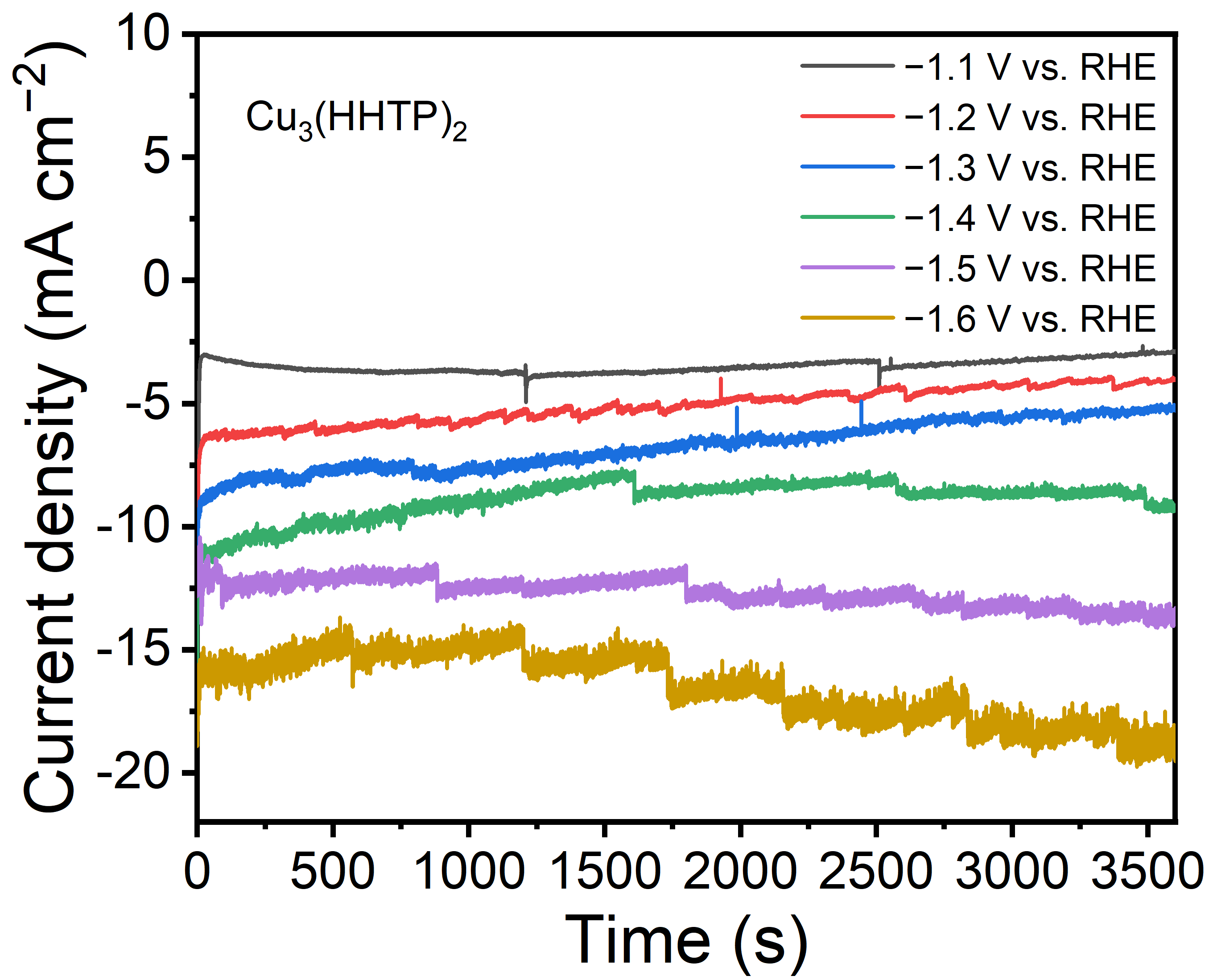


**Fig. S43** Chronoamperometry of the Cu_3_(HHTP)_2_


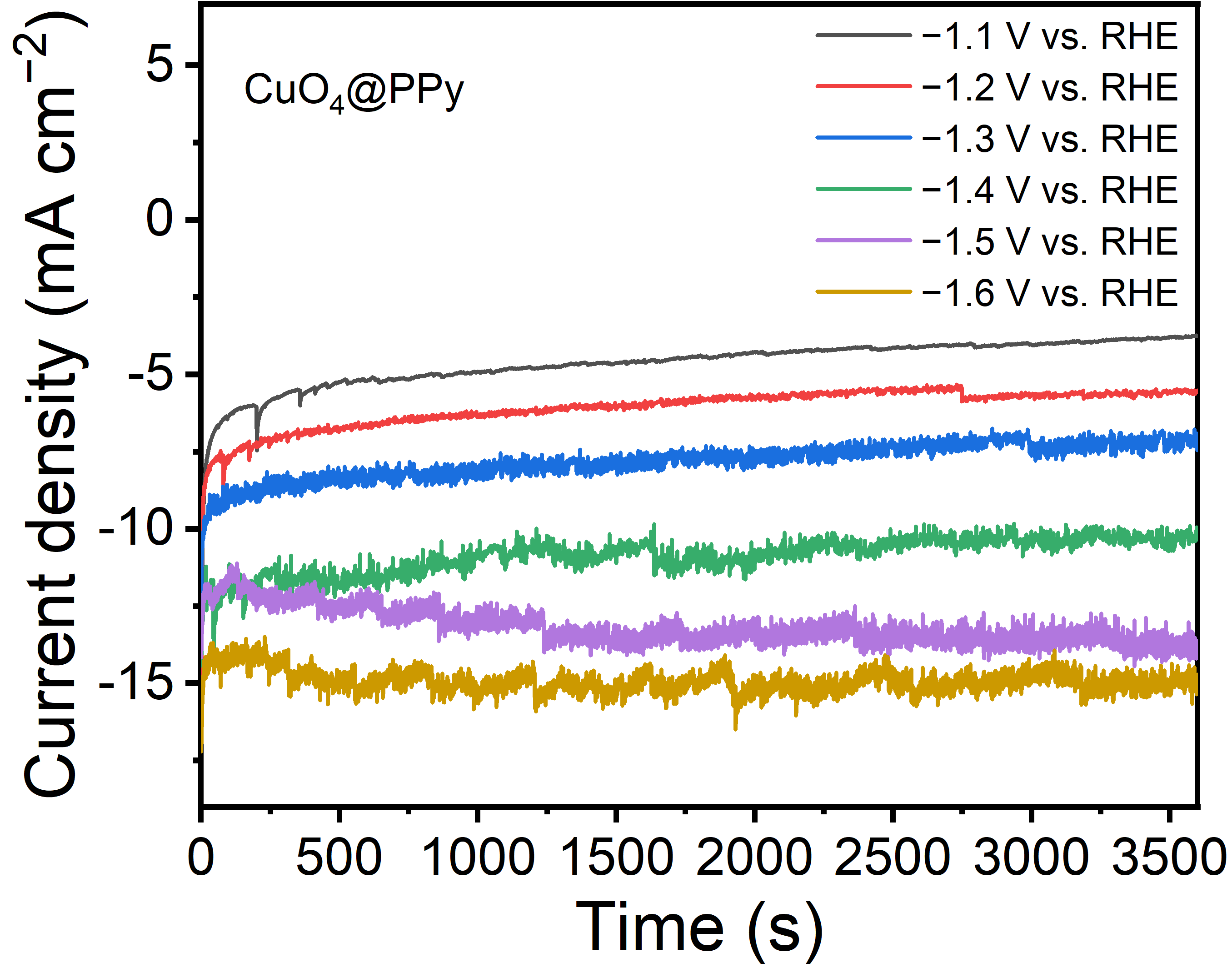


**Fig. S44** Chronoamperometry of the CuO_4_@PPy


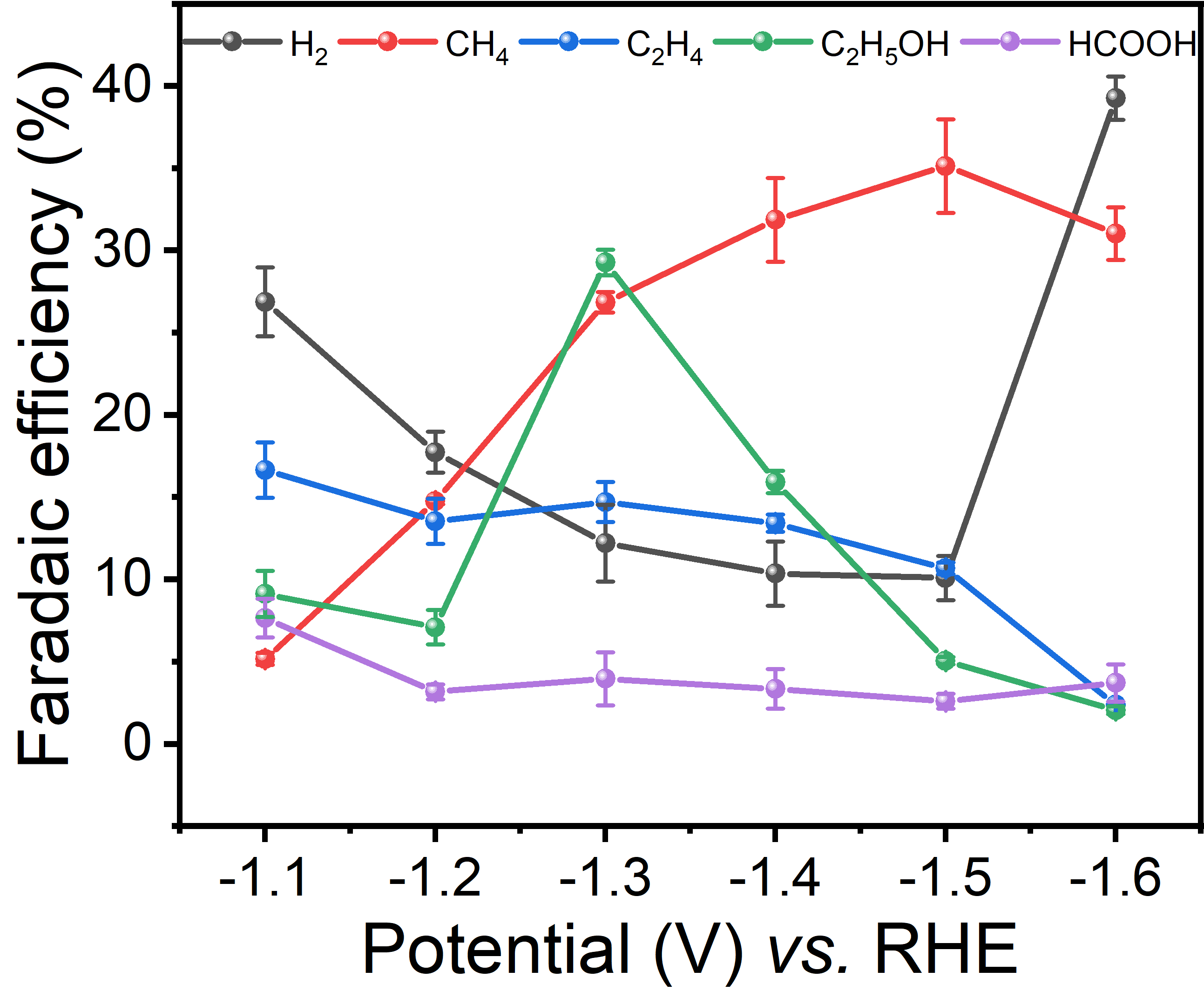


**Fig. S45** FE of different CO_2_RR products of Cu_3_(HHTP)_2_ at various potentials


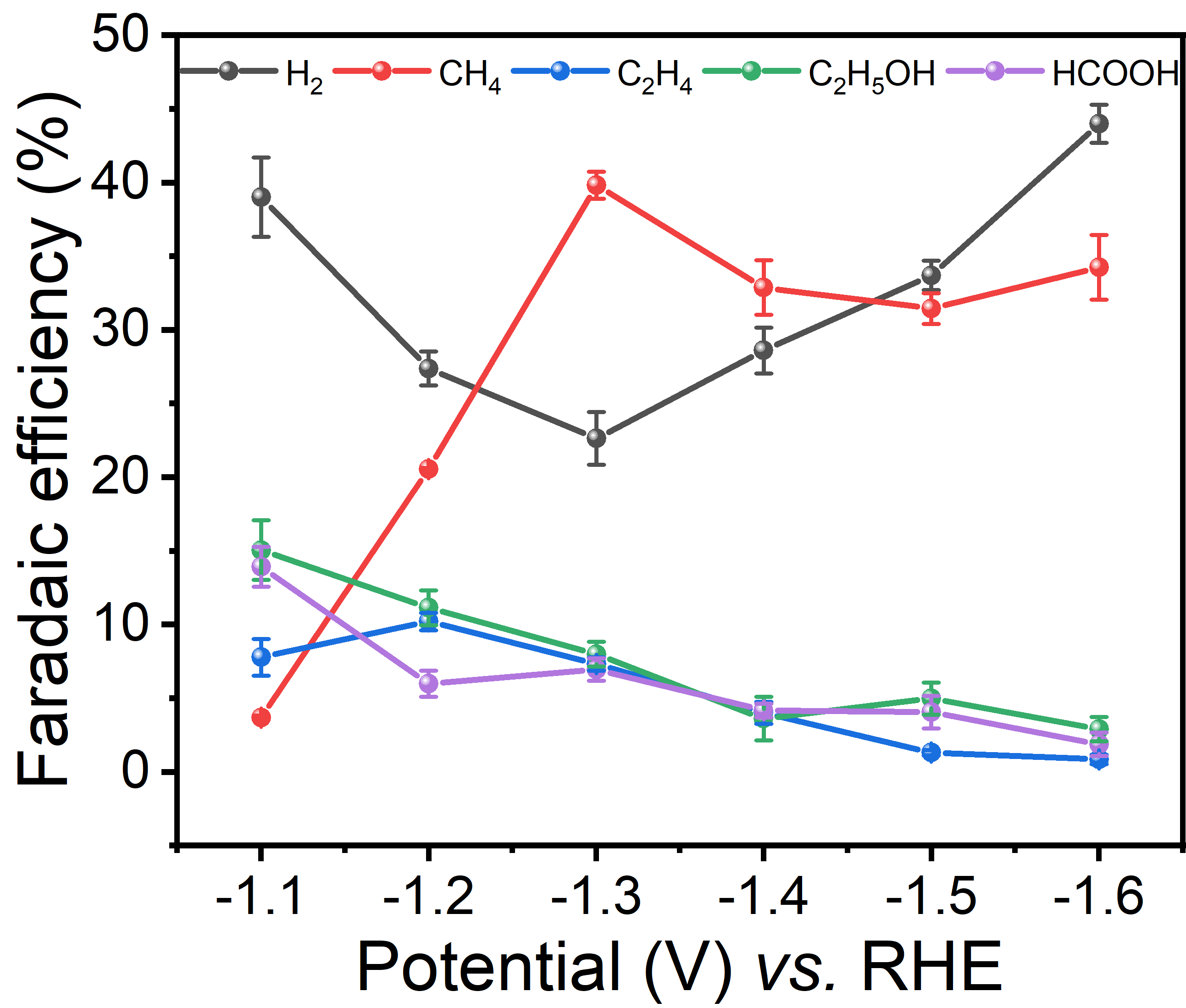


**Fig. S46** FE of different CO_2_RR products of CuO_4_@PPy at various potentials


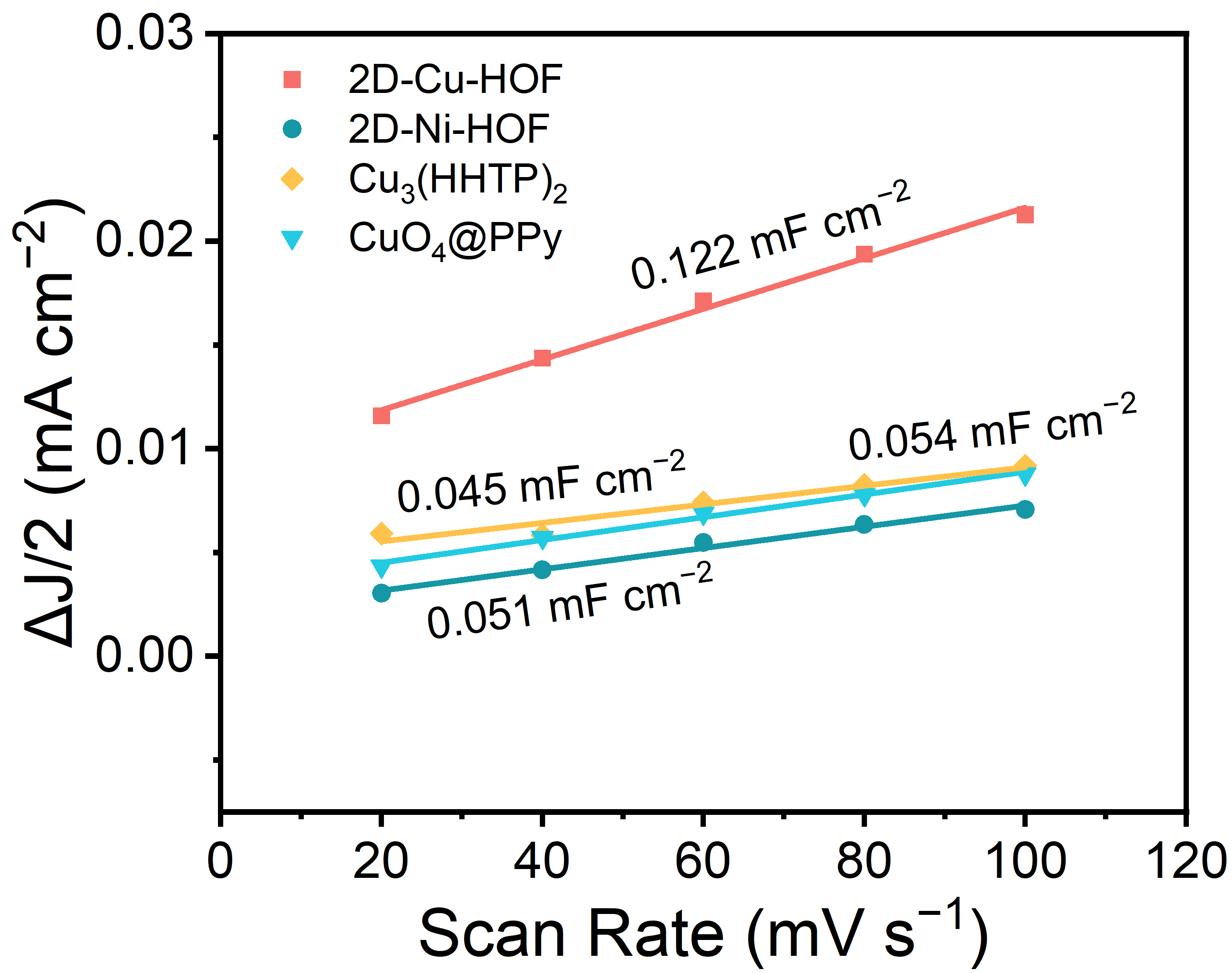


**Fig. S47** ECSAs of 2D-Cu-HOF, 2D-Ni-HOF, Cu_3_(HHTP)_2_, and CuO_4_@PPy


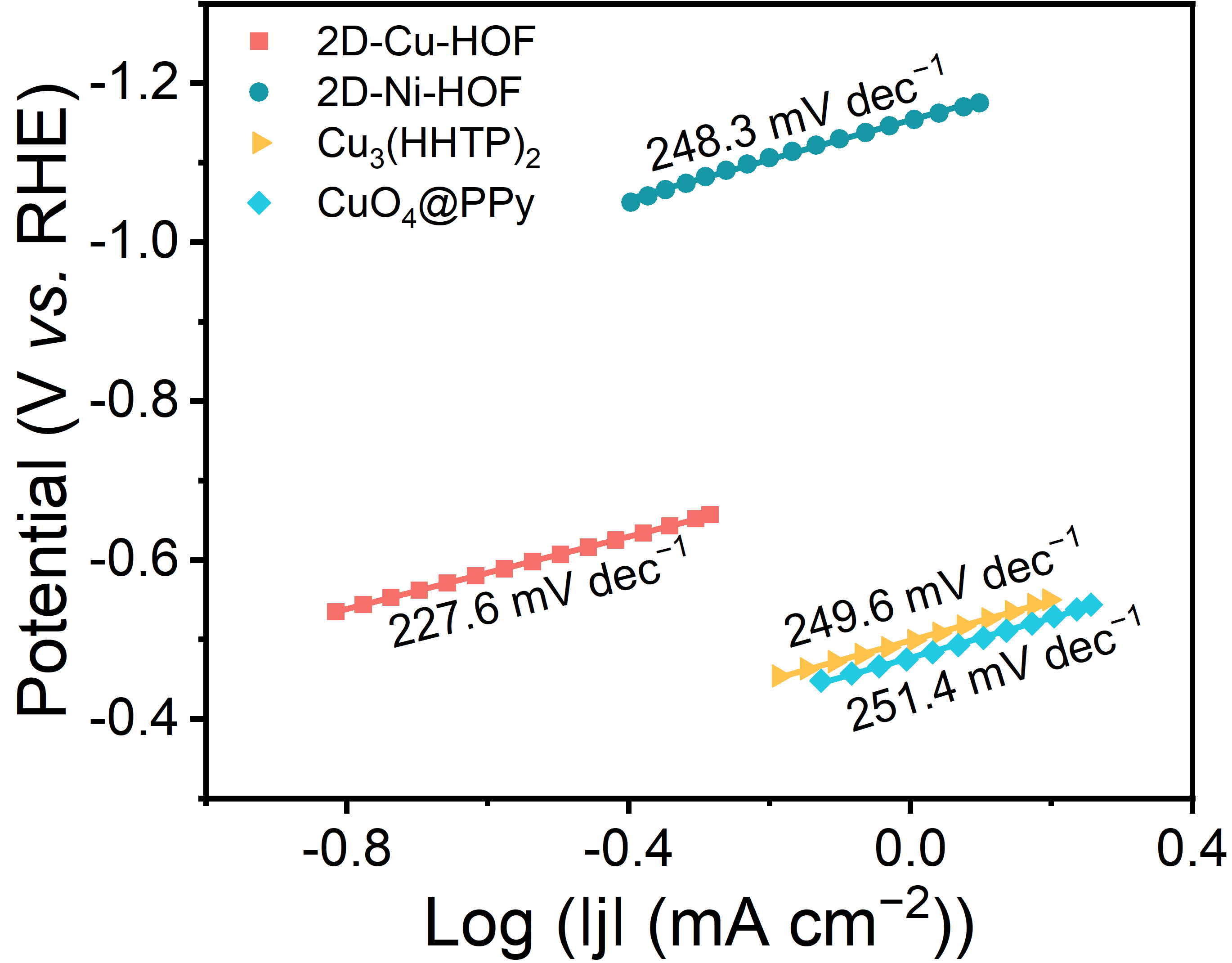


**Fig. S48** Tafel slopes of 2D-Cu-HOF, 2D-Ni-HOF, Cu_3_(HHTP)_2_, and CuO_4_@PPy


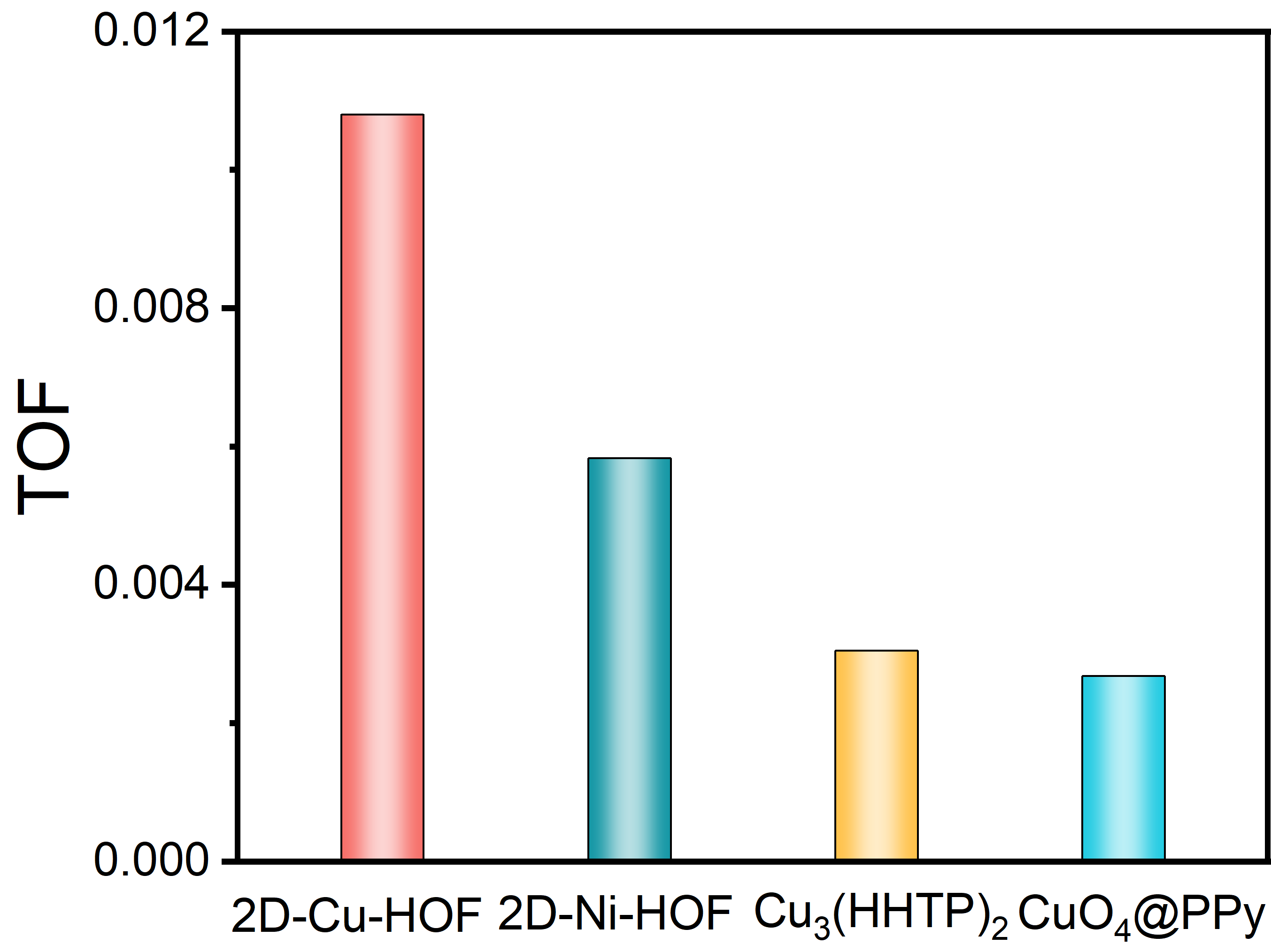


**Fig. S49** TOFs of C_2_ products for 2D-Cu-HOF, 2D-Ni-HOF, Cu_3_(HHTP)_2_, and CuO_4_@PPy at the optimal reaction potential


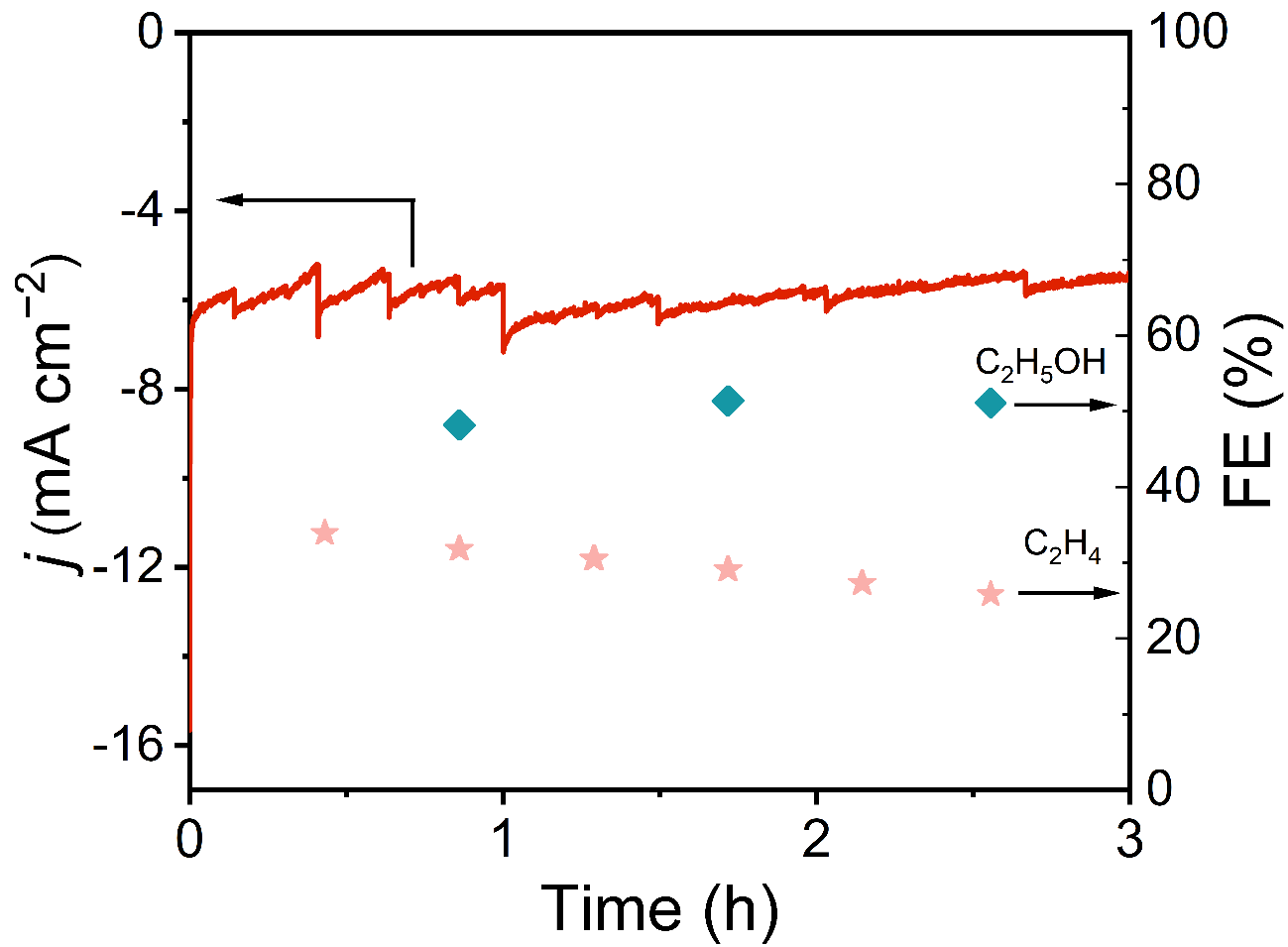


**Fig. S50** FE(C_2_H_5_OH), FE(C_2_H_4_), and current density of 2D-Cu-HOF at −1.2 V vs*.* RHE for 3 hours


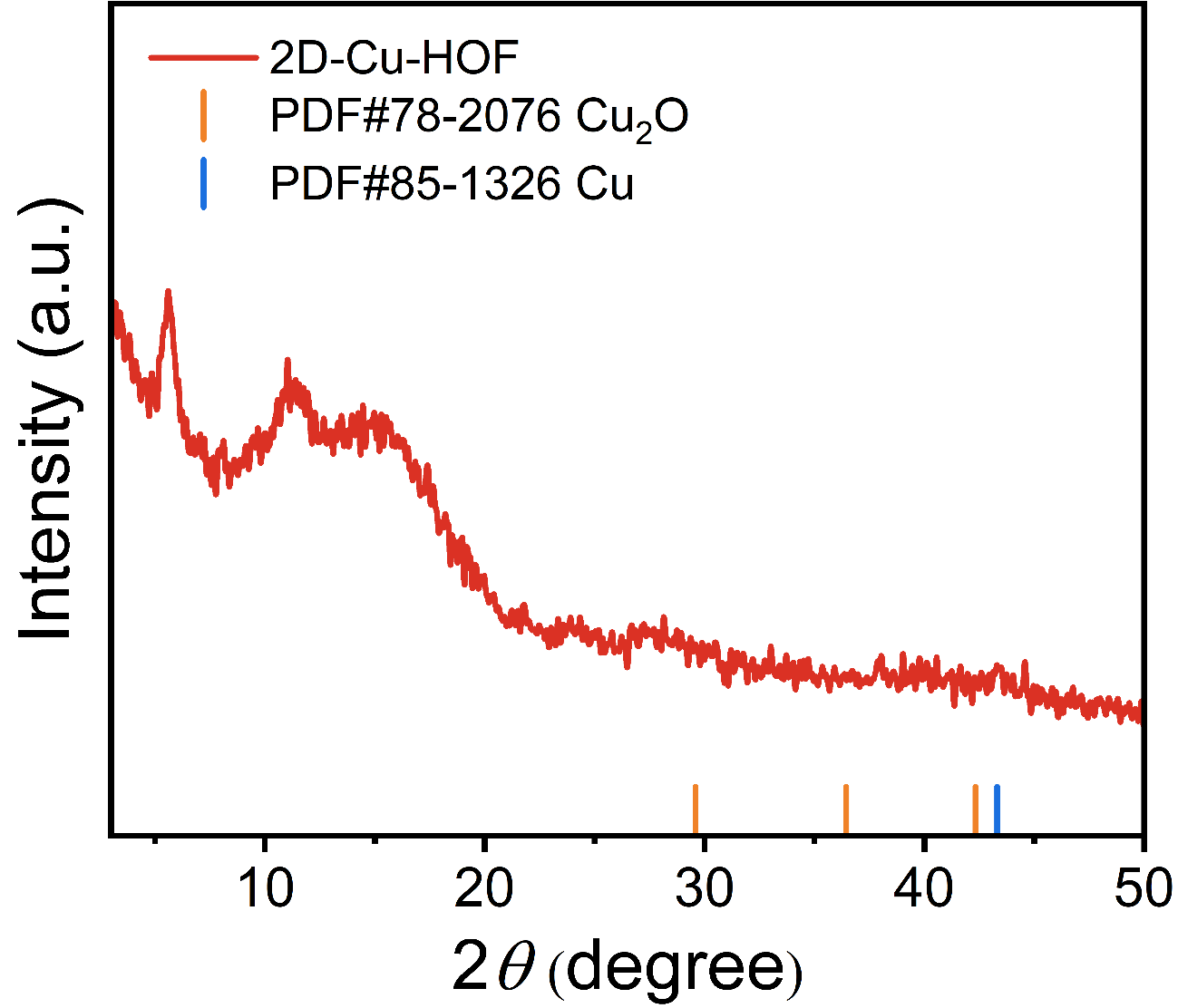


**Fig. S51** PXRD pattern of 2D-Cu-HOF after CO_2_RR


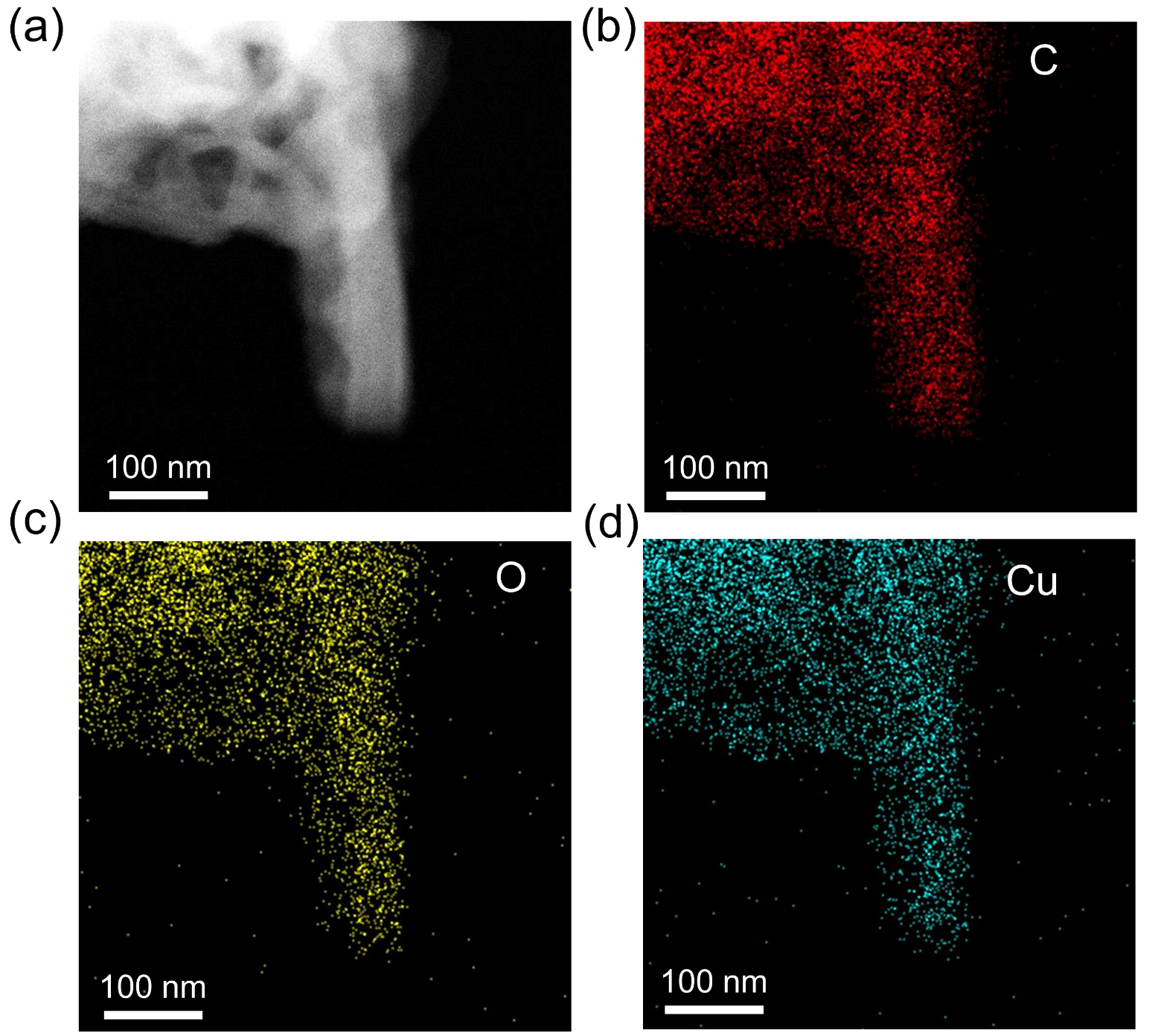


**Fig. S52** TEM elemental mapping of 2D-Cu-HOF after CO_2_RR


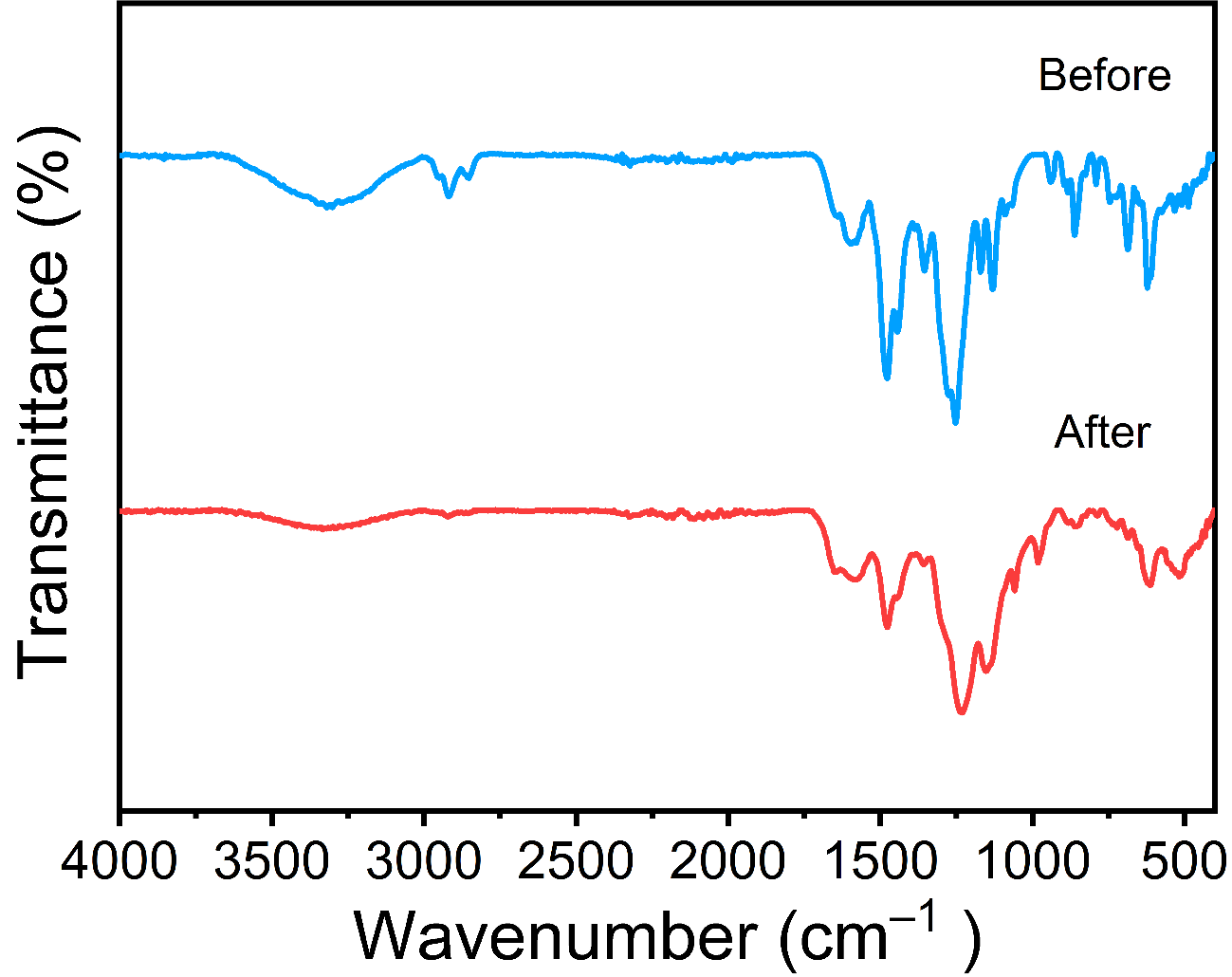


**Fig. S53** FT-IR spectra of 2D-Cu-HOF before (red) and after (blue) CO_2_RR


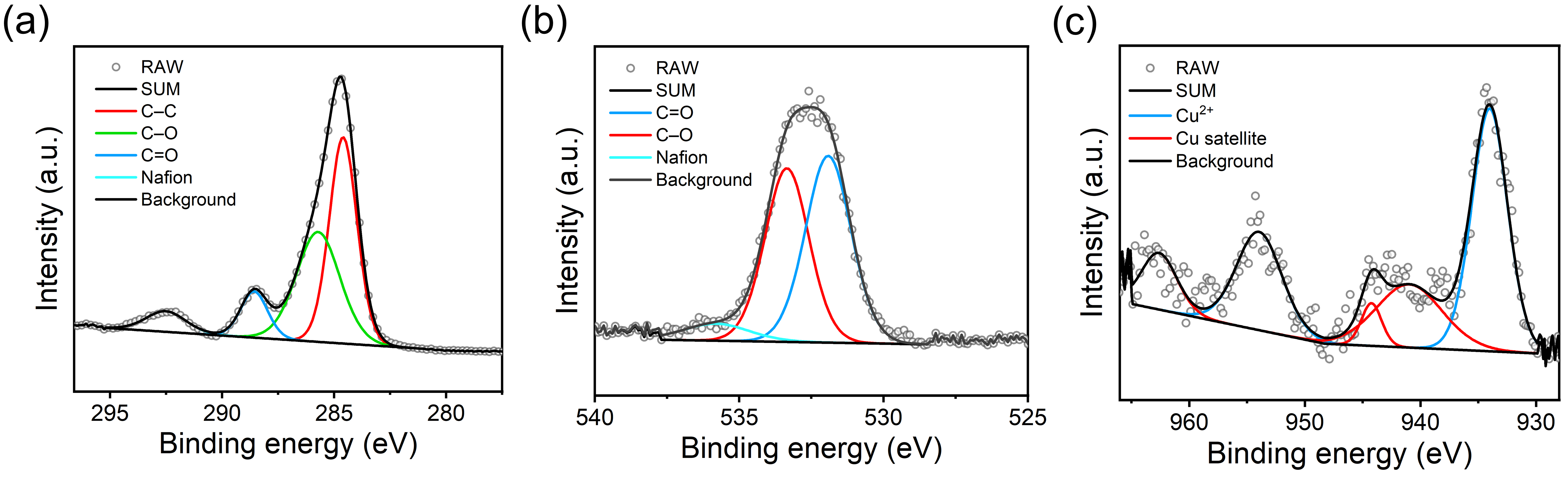


**Fig. S54** High-resolution XPS C 1s (a), O 1s (b) and Cu 2p (c) spectra of 2D-Cu-HOF after CO_2_RR


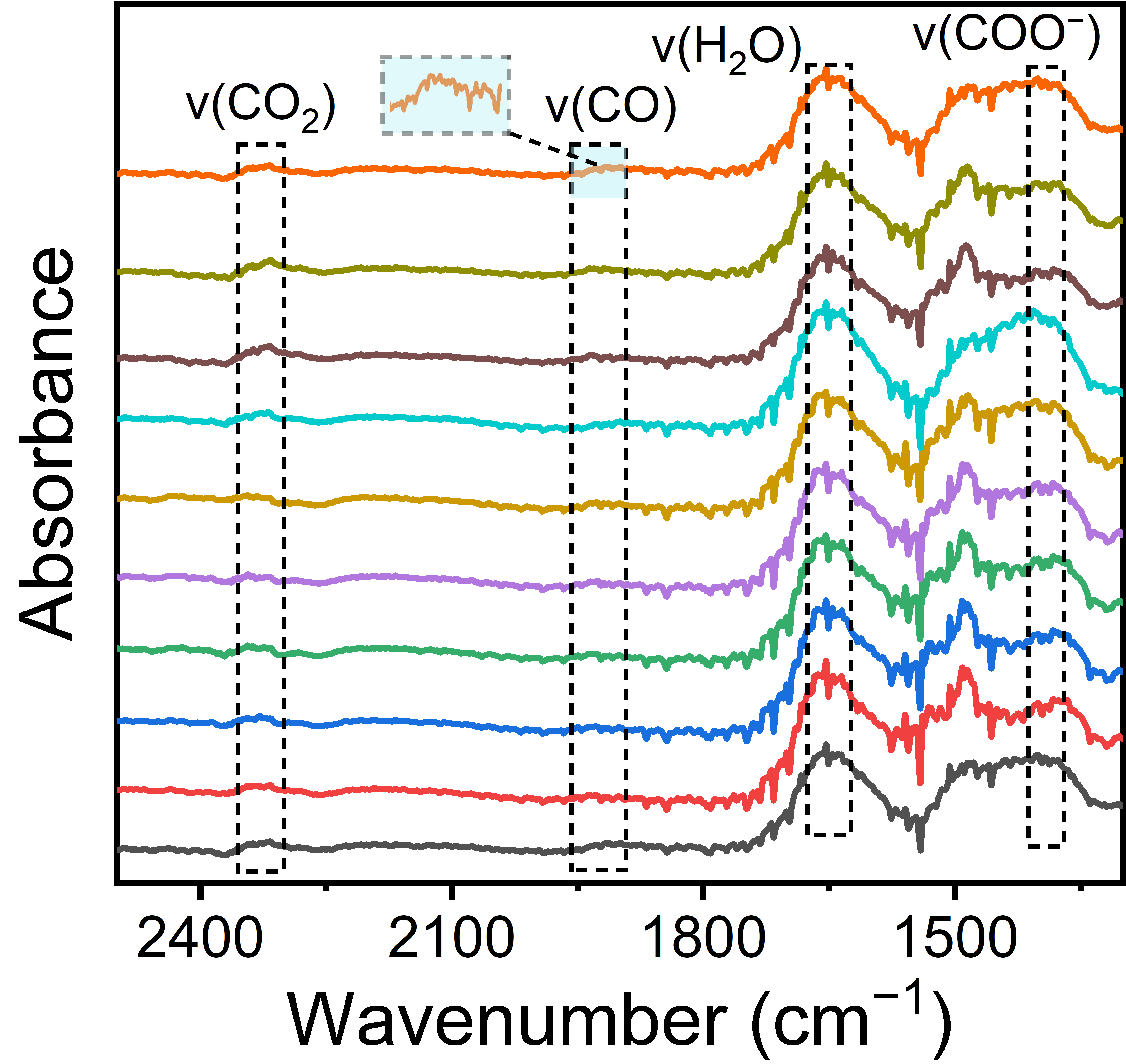


**Fig. S55** Operando ATR-FTIR spectra of 2D-Cu-HOF in CO_2_-saturated 0.1 M KHCO_3_ electrolyte at the potential from 0 to −1.8 V vs. RHE (from bottom to top)


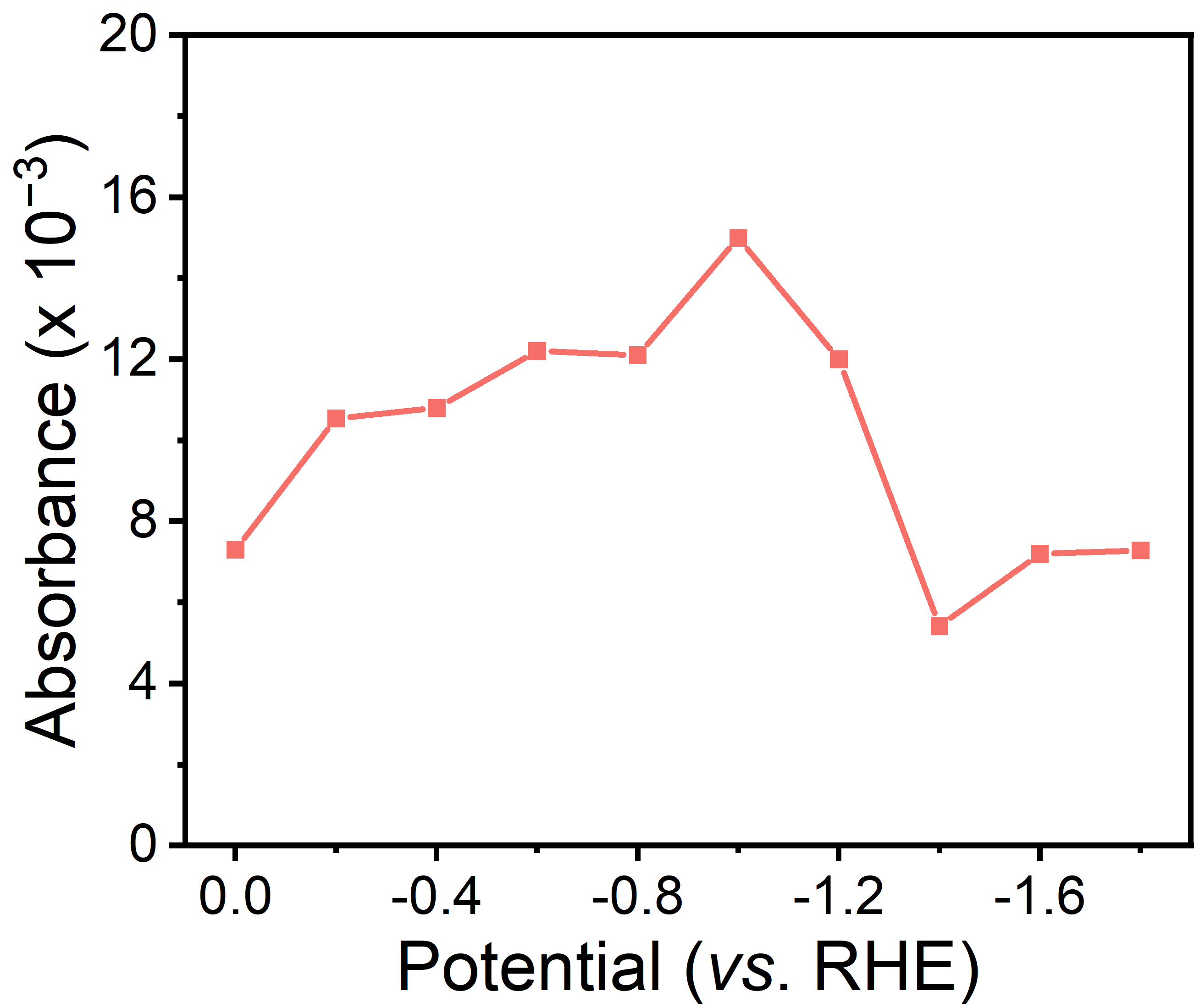


**Fig. S56** The absorbance of *OCCHO of 2D-Cu-HOF in the operando ATR-FTIR spectra at the potential from 0 to −1.8 V vs. RHE


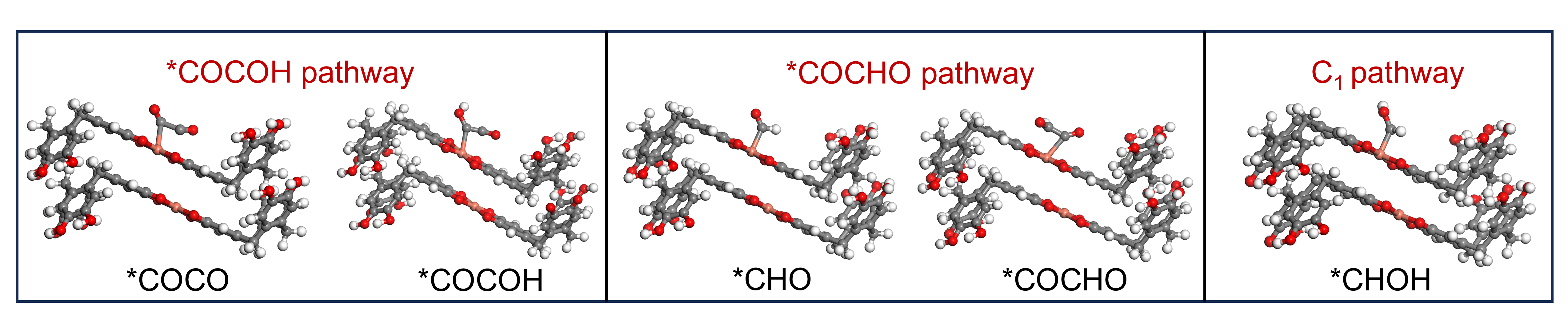


**Fig. S57** The intermediate structures at the surface of 2D-Cu-HOF involved in the *COCOH pathway, *COCHO pathway, and C_1_ pathway as calculated by DFT. The asterisk (*) represents chemisorbed species

**Table S1** cRED data collection and crystallographic details of 2D-Cu-HOF

| Chemical formula | C_42_H_40_CuO_16_ |
| --- | --- |
| Formula weight | 864.31 |
| Temperature | 77 K |
| Crystal system | triclinic |
| Space group | P-1 |
| *a* | 4.7100 Å |
| *b* | 10.640 Å |
| *c* | 18.230 Å |
| *α* | 84.900° |
| *β* | 86.500° |
| *γ* | 80.900° |
| Volume | 897.5 Å^3^ |
| Radiation | Electron (λ = 0.002508 nm) |

**Table S2** The atomic sites of 2D-Cu-HOF

| 2D-Cu-HOF | | | | | | | |
| --- | --- | --- | --- | --- | --- | --- | --- |
| Atom | x/a | y/b | z/c | Atom | x/a | y/b | z/c |
| Cu1 | 0.1000 | 0.0000 | 0.5000 | C16 | 0.3372 | 0.2314 | 0.0739 |
| O2 | 0.7406 | 0.1253 | 0.0007 | C17 | 0.1574 | 0.2279 | 0.1363 |
| O3 | 0.7297 | -0.063 | 0.4418 | C18 | 0.3835 | 0.2692 | 0.4002 |
| O4 | 0.7737 | 0.1667 | 0.4761 | O19 | 0.5942 | 0.7105 | 0.2512 |
| C5 | 0.5520 | 0.0262 | 0.4105 | C20 | 0.3456 | 0.0156 | 0.3581 |
| C6 | 0.5575 | 0.1308 | 0.0611 | C21 | -0.0034 | 0.3712 | 0.3151 |
| O7 | 0.7964 | -0.0862 | 0.1008 | C22 | -0.053 | 0.3530 | 0.1493 |
| C8 | 0.0042 | 0.1060 | 0.2575 | C23 | 0.4337 | 0.6248 | 0.2320 |
| C9 | 0.5768 | 0.1550 | 0.4316 | C24 | 0.1021 | 0.4510 | 0.1823 |
| C10 | 0.1838 | 0.1199 | 0.1875 | C25 | 0.3928 | 0.6215 | 0.1562 |
| C11 | 0.1930 | 0.2495 | 0.3460 | C26 | 0.2288 | 0.5362 | 0.1305 |
| C12 | 0.1780 | 0.1264 | 0.3238 | C27 | 0.2960 | 0.5418 | 0.2827 |
| O13 | 0.5236 | 0.7004 | 0.1051 | C28 | 0.1368 | 0.4517 | 0.2573 |
| C14 | 0.4041 | 0.0127 | 0.1737 | O29 | 0.7100 | 0.6885 | 0.3979 |
| C15 | 0.5903 | 0.0232 | 0.1114 | O30 | 0.7790 | 0.4314 | 0.5132 |

**Table S3** The deconvolution of high-resolution XPS spectrum for 2D-Cu-HOF (FWHM: full width at half maxima)

| **Peak identification** | **Position (eV)** | **FWHM** | **Area (%)** |
| --- | --- | --- | --- |
| Cu 2p_3/2_ | 934.3 | 3.59 | 44.43 |
| Cu 2p_1/2_ | 954.3 | 4.19 | 22.75 |
| Cu(II) satellite | 941.8 | 5.40 | 16.81 |
| Cu(II) satellite | 944.3 | 2.80 | 5.50 |
| Cu(II) satellite | 962.5 | 4.01 | 10.52 |

**Table S4** The deconvolution of high-resolution XPS spectrum for 2D-Ni-HOF (FWHM: full width at half maxima)

| **Peak identification** | **Position (eV)** | **FWHM** | **Area (%)** |
| --- | --- | --- | --- |
| Ni 2p_3/2_ | 855.2 | 2.26 | 20.20 |
| Ni 2p_1/2_ | 872.8 | 2.38 | 8.09 |
| Ni(II) satellite | 860.5 | 7.73 | 36.99 |
| Ni(II) satellite | 878.7 | 10.5 | 34.72 |

**Table S5** EXAFS fitting parameters for 2D-Cu-HOF

| **Sample** | **Bond** | **CN** | **r (Å)** | **σ^2^** | **Δ*E*_0_** | ***R* factor** |
| --- | --- | --- | --- | --- | --- | --- |
| 2D-Cu-HOF | Cu**–**O | 4 | 1.93±0.03 | 0.007 | 3.55 | 0.0083 |
|  | Cu**–**C | 4 | 2.52±0.14 | 0.00059 | 0.56 |  |

CN: coordination numbers; r: bond distance; σ^2^: Debye-Waller factors; Δ*E*_0_: the inner potential correction. *R* factor: goodness of fit. Ѕ_0_^2^ was set to 0.85; Data ranges: 3 ≤ k ≤ 12.3 Å^-1^, 1.0 ≤ R ≤ 3.0 Å.

**Table S6** EXAFS fitting parameters for 2D-Ni-HOF

| **Sample** | **Bond** | **CN** | **r (Å)** | **σ^2^** | **Δ*E*_0_** | ***R* factor** |
| --- | --- | --- | --- | --- | --- | --- |
| 2D-Ni-HOF | Ni–O | 3.84 | 2.04±0.07 | 0.009 | -4.43 | 0.008 |

CN: coordination numbers; R: bond distance; σ^2^: Debye-Waller factors; Δ*E*_0_: the inner potential correction. *R* factor: goodness of fit. Ѕ_0_^2^ was set to 0.87; Data ranges: 3 ≤ k ≤ 12.3 Å^-1^, 1.0 ≤ R ≤ 2.5 Å.

**Table S7** Comparison of the CO_2_RR products and selectivity with other reported Cu-based frameworks

| **Catalysts** | **Products** | **Selectivity (%)** | **Potential**  **(V vs. RHE)** | **Refs.** |
| --- | --- | --- | --- | --- |
| 2D-Cu-HOF | C_2+_ | 82.1 | −1.2V | *This work* |
| Cu-MMT | C_2+_ | 73.75 | −1.15 | *Nano Lett.* 2024, *24*, 1553. |
| def-Cu-HHTP-TFPN-1 | C_2_H_4_ | 54 | −1.2 | *J. Mater. Chem. A*, 2024, *12*, 24549. |
| MIL-53(Cu) | C_2+_ | 55.5 | −1.19 | *J. Am. Chem. Soc.* 2023, *145*, 21442. |
| 2D-*vc*-MOF(Cu) | CH_4_ | 65 | −1.4 | *Angew. Chem. Int. Ed.* 2023*, 62*, e202217958. |
| Ag@BIF-104NSs(Cu) | C_2_H_4_ | 21.43 | −1.2 | *Adv. Energy Mater.* 2023, *13*, 2300088. |
| KB@Cu_3_(HITP)_2_ | C_2_H_4_ | 70 | −1.37 | *Nat. Commun.* 2021, *12*, 6823. |
| HATNA-Cu-MOF | CH_4_ | 78 | −1.5 | *Angew. Chem. Int. Ed.* 2021, *60*, 14473. |
| Cu-THQ | CO | 91 | −0.45 | *Adv. Mater.* 2021, *33*, 2004393. |
| PcCu-Cu-O | C_2_H_4_ | 50 | −1.2 | *J. Am. Chem. Soc.* 2021, *143*, 7242. |

Note. The above Faradaic selectivity is measured in H-cells.

**Table S8** Comparison of the CO_2_RR products and selectivity with other reported Ni-based frameworks

| **Catalysts** | **Products** | **Selectivity (%)** | **Potential**  **(V vs. RHE)** | **Refs.** |
| --- | --- | --- | --- | --- |
| 2D-Ni-HOF | C_2_H_5_OH | 35.6 | −1.3 | *This work* |
| NiPc-Salen(Co)_2_-COF | CO | 97.2 | −1.0 | *Angew. Chem. Int. Ed.* 2024, *63*, e202319472. |
| Ni SAC-1000 | CO | 98.2 | −0.8 | *J. Mater. Chem. A* 2024, *12*, 11090. |
| Ni_50_/Zn_50_ | CO/HCO_2_H | 69/10 | −0.6 | *ACS Appl. Mater. Interfaces* 2024, *16*, 34010. |
| Ni_0.1_Zn_0.9_/ZIF-8 | CO | 34.7 | −1.2 | *Adv. Mater.* 2023, *35*, 2208224. |
| Ni/HNC | CO | 97.2 | −0.7 | *Nano Res.* 2023, *16*, 8970. |
| MWCNT-Por-COF-Ni | CO | 56.4 | −0.6 | *Appl. Catal., B* 2022, *303*, 120897. |
| TPPDA-NiPor-COF | CO | 76 | −0.9 | *Inorg. Chem. Front.* 2022, *9*, 3217. |
| NiPc-COF | CO | 99.1 | −0.9 | *Small* 2020, *16*, 2005254. |
| Ni-Pc-Cu-O | CO | 56 | −0.74 | *J. Am. Chem. Soc.* 2020, *142*, 21656. |

Note. The above Faradaic selectivity is measured in H-cells.

**Table S9** Elemental content of Cu in Cu_3_(HHTP)_2_ and CuO_4_@PPy (Cu was analyzed by ICP-MS)

| **Cu (wt.%)** | **Cu_3_(HHTP)_2_** | **CuO_4_@PPy** |
| --- | --- | --- |
| Calcd. | 23.18 | 11.36 |
| Found | 23.10 | 12.05 |

**Table S10** Elemental analysis of copper catecholate (CuO_4_)

|  | **C (wt.%)** | **H (wt.%)** |
| --- | --- | --- |
| Calcd. | 51.52 | 2.88 |
| Found | 50.74 | 2.71 |

**Table S11** The deconvolution of high-resolution XPS spectrum for 2D-Cu-HOF after CO_2_RR (FWHM: full width at half maxima)

| **Peak identification** | **Position (eV)** | **FWHM** | **Area (%)** |
| --- | --- | --- | --- |
| Cu 2p_3/2_ | 934.0 | 3.65 | 42.98 |
| Cu 2p_1/2_ | 954.0 | 4.65 | 22.18 |
| Cu(II) satellite | 941.0 | 6.70 | 20.55 |
| Cu(II) satellite | 944.2 | 2.06 | 4.26 |
| Cu(II) satellite | 962.6 | 3.65 | 10.03 |

## Supplementary References

1. P.T. Lee, J.E. Thomson, A. Karina, C. Salter, C. Johnston et al., Selective electrochemical determination of cysteine with a cyclotricatechylene modified carbon electrode. Analyst **140**, 236-242 (2015). <https://doi.org/10.1039/C4AN01835D>
2. G.T. Illa, S. Hazra, P. Satha, C.S. Purohit, Hydrogen-bonded molecular capsules: probing the role of water molecules in capsule formation in modified cyclotricatechylene. CrystEngComm **19**, 4759-4765 (2017). <https://doi.org/10.1039/C7CE01075C>
3. B. Ravel, M. Newville, ATHENA, ARTEMIS, HEPHAESTUS: Data analysis for X-ray absorption spectroscopy using IFEFFIT. J. Synchrotron Radiat. **12**, 537-541 (2005). <https://doi.org/10.1107/S0909049505012719>
4. G. Kresse, D. Joubert, From ultrasoft pseudopotentials to the projector augmented-wave method. Phys. Rev. B **59**, 1758-1775 (1999). <https://doi.org/10.1103/PhysRevB.59.1758>
5. G. Kresse, J. Furthmüller, Efficiency of ab-initio total energy calculations for metals and semiconductors using a plane-wave basis set. Comput. Mater. Sci. **6**, 15-50 (1996). <https://doi.org/10.1016/0927-0256(96)00008-0>
6. J.P. Perdew, K. Burke, M. Ernzerhof, Generalized gradient approximation made simple. Phys. Rev. Lett. **77**, 3865-3868 (1996). <https://doi.org/10.1103/PhysRevLett.77.3865>
